# Purine salvage promotes treatment resistance in H3K27M-mutant diffuse midline glioma

**DOI:** 10.1186/s40170-024-00341-7

**Published:** 2024-04-09

**Authors:** Erik R. Peterson, Peter Sajjakulnukit, Andrew J. Scott, Caleb Heaslip, Anthony Andren, Kari Wilder-Romans, Weihua Zhou, Sravya Palavalasa, Navyateja Korimerla, Angelica Lin, Alexandra O’Brien, Ayesha Kothari, Zitong Zhao, Li Zhang, Meredith A. Morgan, Sriram Venneti, Carl Koschmann, Nada Jabado, Costas A. Lyssiotis, Maria G. Castro, Daniel R. Wahl

**Affiliations:** 1 Doctoral Program in Cancer Biology, University of Michigan, Ann Arbor, MI USA; 2 Department of Radiation Oncology, University of Michigan, Ann Arbor, MI USA; 3 Rogel Cancer Center, University of Michigan, Ann Arbor, MI USA; 4 Massachusetts College of Pharmacy and Health Sciences, Boston, MA USA; 5 Department of Molecular and Integrative Physiology, University of Michigan, Ann Arbor, MI USA; 6 Department of Cellular and Molecular Biology, University of Michigan, Ann Arbor, MI USA; 7 Department of Pathology, University of Michigan, Ann Arbor, MI USA; 8 Department of Pediatrics, University of Michigan, Ann Arbor, MI USA; 9 Department of Pediatrics, McGill University, Montreal, Quebec Canada; 10 Department of Human Genetics, McGill University, Montreal, Quebec Canada; 11 Department of Neurosurgery, University of Michigan, Ann Arbor, MI USA; 12 Medical Science Unit I, 1301 Catherine Street, Rm 4433, Ann Arbor, MI 48109 USA

**Keywords:** Diffuse midline glioma, H3K27M, Radiation therapy resistance, Purine metabolism

## Abstract

**Background:**

Diffuse midline gliomas (DMG), including diffuse intrinsic pontine gliomas (DIPGs), are a fatal form of brain cancer. These tumors often carry a driver mutation on histone H3 converting lysine 27 to methionine (H3K27M). DMG-H3K27M are characterized by altered metabolism and resistance to standard of care radiation (RT) but how the H3K27M mediates the metabolic response to radiation and consequent treatment resistance is uncertain.

**Methods:**

We performed metabolomics on irradiated and untreated H3K27M isogenic DMG cell lines and observed an H3K27M-specific enrichment for purine synthesis pathways. We profiled the expression of purine synthesis enzymes in publicly available patient data and our models, quantified purine synthesis using stable isotope tracing, and characterized the in vitro and in vivo response to *de novo* and salvage purine synthesis inhibition in combination with RT.

**Results:**

DMG-H3K27M cells activate purine metabolism in an H3K27M-specific fashion. In the absence of genotoxic treatment, H3K27M-expressing cells have higher relative activity of *de novo* synthesis and apparent lower activity of purine salvage demonstrated via stable isotope tracing of key metabolites in purine synthesis and by lower expression of hypoxanthine-guanine phosphoribosyltransferase (HGPRT), the rate-limiting enzyme of purine salvage into IMP and GMP. Inhibition of *de novo* guanylate synthesis radiosensitized DMG-H3K27M cells in vitro and in vivo*.* Irradiated H3K27M cells upregulated HGPRT expression and hypoxanthine-derived guanylate salvage but maintained high levels of guanine-derived salvage. Exogenous guanine supplementation decreased radiosensitization in cells treated with combination RT and *de novo* purine synthesis inhibition. Silencing HGPRT combined with RT markedly suppressed DMG-H3K27M tumor growth in vivo*.*

**Conclusions:**

Our results indicate that DMG-H3K27M cells rely on highly active purine synthesis, both from the *de novo* and salvage synthesis pathways. However, highly active salvage of free purine bases into mature guanylates can bypass inhibition of the *de novo* synthetic pathway. We conclude that inhibiting purine salvage may be a promising strategy to overcome treatment resistance in DMG-H3K27M tumors.

**Supplementary Information:**

The online version contains supplementary material available at 10.1186/s40170-024-00341-7.

## Background

Diffuse midline gliomas (DMG) are pediatric high-grade gliomas that arise in midline structures of the brain including the thalamus, cerebellum, and pons [1–3]. In 2016, the World Health Organization described a subtype of DMG that carries a driver missense mutation in the tail domain of Histone H3 that converts the 27^th^ residue from lysine (K) to methionine (M), termed H3K27M [1, 4]. Patients with DMG-H3K27M carry a dire prognosis with over 90% dying within 2 years of diagnosis [2]. Treatment options are limited for patients with DMG-H3K27M tumors. Surgical resection is often unfeasible due to the eloquent function of the midline tissues in which they arise [2, 3, 5]. DMG-H3K27M tumors also derive minimal benefit from systemic therapies [3, 5–7]. Radiation therapy (RT) is currently the only treatment modality that provides meaningful benefit to patients and is the current standard of care [8–12]. However, RT typically only extends patient survival by months, and tumors regrow within the high-dose radiation field [8, 12, 13]. This suggests that DMG-H3K27M tumors can effectively adapt to and resist RT, leading to regrowth. Simply increasing the RT dosage given is not feasible owing to limiting toxicity in normal tissue. Selectively increasing the sensitivity of DMG-H3K27Ms to RT could help improve patient outcomes.

Cellular metabolism controls RT efficacy in various tumor types, including adult brain malignancies [14–17]. The H3K27M mutation causes a global shift in the epigenome, leading to large-scale gene expression changes. This is characterized by global hypomethylation of the H3K27 residue in promoter regions of H3K27M cells which is accompanied by increased activating H3K27 acetylation marks [4, 18–23]. These alterations cause metabolic shifts specific for H3K27M-expressing tumors [24]. Such shifts include increased dependence on methionine and pyrimidine metabolism, along with classically cancer-co-opted pathways such as glycolysis and the TCA cycle [25–27]. How and whether these H3K27M-driven metabolic changes confer radiation resistance to DMGs is unknown.

Here, we identify purine metabolism as an H3K27M-specific metabolic vulnerability that governs the RT response. Cells can synthesize the two main classes of purines (adenylates and guanylates) *de novo* from metabolic building blocks or salvage them from pre-formed nucleobases. We find that H3K27M-expressing cells preferentially utilize *de novo* guanylate synthesis during unperturbed growth to a higher degree than H3K27M-KO cells, likely due to increased expression of the enzymes utilized in *de novo* synthesis and decreased expression of those used in salvage. Inhibitors of *de novo* guanylate synthesis sensitized DMG-H3K27M models to RT in vitro and in vivo but do not cure tumors. Unexpectedly, we find that H3K27M cells have high rates of hypoxanthine and guanine salvage after RT, likely mediating resistance to inhibition of *de novo* guanylate synthesis. Knockdown of the rate limiting enzyme in guanylate salvage, hypoxanthine-guanine phosphoribosylatransferase (HGPRT), increases RT efficacy in orthotopic H3K27M xenografts. These findings indicate that guanylate salvage inhibition may be a promising strategy to overcome RT resistance in DMG-H3K27M.

## Methods

### Cell lines and tissue culture

Patient-derived H3K27M-isogenic (H3K27M and H3K27M-KO) cell line pairs (DIPGXIII and BT245) were generous gifts from Dr. Nada Jabado (McGill University) [20] and were cultured in Tumor Stem Media (TSM) as previously described [19]. Both H3K27M-isogenic cell lines were *TP53*-mutated (DIPGXIII:K132R, BT245:R117S) [20, 28].

### DIPGXIII-GFP/LUC and DIPGXIII-GPF/LUC-shHPRT1 cell lines

DIPGXIII H3K27M-expressing cells were plated in TSM media containing polybrene transfection reagent and 10X ready-made lentiviral stock containing expression constructs for GFP and firefly luciferase (GFP/LUC) (Lenti-GF1-CMV-VSVG) or ready-made shHPRT1 viral vector stock (Santa Cruz Biotechnology Cat #: sc-40679-V). Cells were centrifuged and washed and expanded in 15cm plates 24hrs later. Successful transduction was confirmed by GFP expression or puromycin selection and loss of HGPRT protein.

### Western blotting

H3K27M-isogenic cells were lysed in RIPA buffer containing protease (cOmplete, Roche) and phosphatase inhibitors (phoSTOP, Roche) on ice with mechanical disruption at the start of incubation. Lysates were clarified and protein concentration was determined using BCA assay (ThermoFisher). SDS-PAGE was performed using at least 12.5ug protein on 4-20% Tris-Glycine gels. Proteins were semi-dry transferred onto nitrocellulose for 1.5hrs at 24V. Membranes were blocked in non-fat dry milk in TBST before incubation with primary antibody overnight at 4C. Membranes were washed with TBST before incubation with secondary antibody. West Pico Plus (ThermoFisher) ECL reagent and X-Ray film were used to measure expression as previously described [16]. Antibodies used: H3K27M (Cell Signaling, Cat#: 74829), H3K27me3 (EMD-Millipore, Cat#: 07-449), inosine monophosphate dehydrogenase 1 (IMPDH1) (Cell Signaling, Cat#: 35914), HPRT1 (Invitrogen, Cat#: PA5-22281), beta-Actin (Santa Cruz, Cat#: sc-47778), ENT1 (Abnova, Cat#: PAB2255), ENT2 (Abcam, Cat# ab181192), ENT4 (Bioss, Cat#: bs-4176R).

### Histone purification

Histone purification was performed as described previously [24]. H3K27M isogenic cells (>1.0x10^6) were suspended in 1mL hypotonic lysis buffer and lysed by mechanical shearing on a rotator at 4C. Nuclei were isolated and suspended in 0.4N H_2_SO_4_ overnight to precipitate histones. Samples were clarified and trichloroacetic acid was added dropwise to the histone-containing supernatant, which were mixed and incubated on ice. Histones were pelleted, washed with ice-cold acetone, and air-dried. Histones were resuspended in ddH2O. Protein content was quantified using a BCA assay.

### Steady-state metabolomics

H3K27M-isogenic cells (2-3x10^6 cells/biological replicate) were plated in 10cm dishes in TSM media and were grown to be small neurospheres (approximately 2 days in culture). Media was changed 2hrs before treatment. Cells were irradiated with 4Gy RT using a Philips RT250 (Kimtron Medical) at a dose rate of approximately 2 Gy/minute at the University of Michigan Rogel Comprehensive Cancer Center Experimental Irradiation Shared Resource (Ann Arbor, MI). Cells were incubated for 2hrs at 37°C. One plate from each group was analyzed by BCA assay quantify protein for standardization. After incubation, all cells were collected in individual 15mL conical tubes and pelleted. Supernatants were removed and metabolites extracted with ice-cold 80% methanol on dry ice. Pellets were resuspended and incubated for on dry ice. Samples were centrifuged to clarify the metabolite extract and supernatants were transferred to new 15mL tubes. This step was repeated twice to ensure clarity. Metabolite extracts equating to roughly 1000μg of protein were transferred to 1.5mL microcentrifuge tubes before drying in a speed vacuum centrifuge or under a nitrogen blower.

Metabolic studies of normal human astrocytes (NHA) conditioned media were performed by plating cells (3x10^6 cells/replicate) in 10cm dishes and allowing them to adhere overnight. The next day, old media was changed, and cells were given fresh, DMEM media (guanine-free) containing 10% dialyzed FBS. RT-treated groups were immediately given a 4Gy single dose of radiation as previously described and were incubated for 24hrs. Conditioned media was collected from both RT-treated and untreated controls and clarified before mixing 1:4 with ice-cold 100% methanol to bring the final methanol concentration to 80%. Samples of unconditioned media were also prepared. These samples were then dried under a nitrogen blower.

Liquid chromatography/mass spectrometry (LC/MS) analyses were performed as previously described [29]. Agilent MassHunter Quantitative Analysis B.09.00 QqQ software was used to integrate and quantitate areas (Agilent Technologies).

### Steady-state metabolomics data analysis

Metabolite lists were trimmed to exclude metabolites that fell below the noise threshold. Remaining metabolite abundances were median centered. H3K27M isogenic cells were analyzed in pairs. All metabolites whose abundance changed by a Log2 FC of |0.15| following RT in either H3K27M or H3K27M-KO cells were selected and combined into a single list. These lists were ordered by the absolute value of difference in post-RT Log2FC between K27M and KO cells. The top 25 metabolites were selected for pathway enrichment analysis using MetaboAnalyst 5.0 where the top 10 most significantly represented pathways were selected [30]. Statistical analyses and heatmap construction were performed using GraphPad Prism 10.0.

### Stable isotope tracing

H3K27M isogenic cells (>3.0-4.0x10^6 cells/replicate) were plated in 10cm dishes in TSM and grown into small neurospheres. Approximately 30min before RT, cells were pelleted and washed and replated with TSM media with stable isotope tracer molecules replacing the naturally occurring metabolites (21mM U^13^C glucose, 2mM 2,8-deuterium hypoxanthine (2D-Hpx), 4mM ^15^N-glutamine (^15^N-Gln), and 100µM ^13^C_8_-guanine (^13^C_8_ -Gua) [Cambridge Laboratories Inc, Cat#: CLM-1396-PK, DLM-8658, NLM-557, and CLM-1019-PK, respectively]). One replicate plate from each group was set aside and given unlabeled TSM media to act as unlabeled control samples. Untreated and irradiated cells (4Gy) were incubated at 37°C for 3hrs after RT at which point metabolites were extracted.

LC/MS Analysis was performed on an Agilent system consisting of an Infinity Lab II UPLC with a 10-port valve coupled with a 6545 QToF mass spectrometer (Agilent Technologies) using a JetStream ESI source in negative mode. Source parameters: Gas Temp: 250 °C, Gas Flow: 13 L/min, Nebulizer: 35 psi, Sheath Gas Temp: 325 °C, Sheath Gas Flow: 12 L/min, Capillary: 3500 V, Nozzle Voltage: 1500 V.

Chromatographic separation was performed on an Agilent ZORBAX RRHD Extend 80Å C18, 2.1 × 150 mm, 1.8μm column with an Agilent ZORBAX SB-C8, 2.1 mm × 30 mm, 3.5μm guard column. The column temperature was 35 °C. Mobile phase A consisted of 97:3 water/ methanol and mobile phase B was 100% methanol; both A and B contained tributylamine and glacial acetic acid at concentrations of 10mM and 15mM, respectively. The column was backflushed with mobile phase C (100% acetonitrile, no additives) between injections for column cleaning.

The LC gradient was as follows: 0-2.5 min, 0% B; 2.5-7.5 min, linear ramp to 20% B min, 7.5-13 min, linear ramp to 45% B; 13-21 min linear ramp to 99% B and held at 99% B until 25 min. At 25 minutes, the 10-port valve was switched to reverse flow (back-flush) through the column, and the solvent composition changed to 95% C and held there for 3 min. From 28 to 28.5 min, the flow rate was ramped to 0.8 mL/min, held until 32.5 min, then reduced to 0.6mL/min. From 32.5 to 33.25 the solvent was ramped from 99% to 0% C while flow was simultaneously ramped down from 0.6-0.4mL/min and held until 39.9 min., at which point flow rate was returned to starting conditions at 0.25mL/min. The 10-port valve was returned to restore forward flow through the column at 40 min. An isocratic pump was used to introduce reference mass solution through the reference nebulizer for dynamic mass correction. Total run time was 30 min. The injection volume was 5μL. Data was analyzed using MassHunter ProFinder 8.0 software (Agilent Technologies).

### Tumor expression data

mRNA-seq Z-score expression and patient survival data from Mackay et al. (2017) was obtained using PedCBioPortal (Children’s Hospital of Philadelphia Research Initiative) [2, 31, 32]. Data was filtered for patients who had confirmed WT Histone H3 or H3K27M mutant (*H3F3A* or *HIST1H3B*) and the Z-score for each gene of interest was extracted and sorted based on mutational status. Statistical analyses were performed using a two-tailed t-test in GraphPad Prism 10.0.

### Long-term neurosphere assay and live cell imaging

H3K27M cells (3x10^5-4x10^5) were cultured in 6-well dishes for 48hrs until they formed small spheres which were then treated with mycophenolic acid (MPA) for 6hrs prior to irradiation. Approximately 18hrs later, spheres were dissociated and replated at low density (500 cells/well) in 96-well plates. Guanine rescue experiments were performed by culturing cells in TSM media supplemented with 10µM-100µM guanine (as indicated), added at the time of MPA treatment and continued through the duration of the experiment. Plates were incubated for 5-10 days in a cell culture incubator for CellTiter-Glo 3D (Promega) analysis or in a BioSpa incubator (Agilent Technologies) where they were imaged every 24hrs in a Cytation 5 plate reader with the 4x objective. Gen5 software (Agilent Technologies) was used to analyze the data. A 2D area threshold of 2600um^2 was used to pick spheres, approximating the 50-colony threshold used in standard clonogenic survival assays. End-point sphere numbers were used to calculate the surviving fraction of cells. The Dmid of the 0μM MPA control was divided by the Dmid of the MPA-treated groups to calculate the Enhancement Ratio (ER) to display changes in RT efficacy.

### Stereotactic orthotopic implantation

Rag1-KO C57BL/6 mice were anesthetized and provided carprofen analgesic before removing the scalp fur and sterilizing the incision site. An incision was made along the midline of the scalp and a small hole in the skull was made using an electric burr hole drill or handheld drill bit at coordinates 2-3mm lateral, 0.5-1mm rostral or caudal (depending on the injection rig used) from the bregma. Approximately 1.5x10^5-2.0x10^5 cells (~5.0x10^4 cells/μL) in a volume of 3μL were implanted in the cortex at depth of approximately 2.5mm using 10μL Hamilton syringes. After injections, wounds were sutured and were given triple antibiotic ointment. Atipamezole was given reverse the anesthetic in heated cages. Mice were given diet gel supplement and monitored for 10 days, including a second dose of carprofen the day following the surgery. For some experiments, a PhD Ultra multi-syringe pump (Harvard Apparatus) was utilized to facilitate simultaneous implantation of up to 8 mice at once. A minimum of 6 mice were used per treatment group in subsequent in vivo experiments.

### Bioluminescent imaging (BLI) and mouse treatment

Mice were administered sterile-filtered 30mg/mL luciferin solution (Syd Laboratories) via IP injection. Ten minutes post-injection, BLI signal was measured using an IVIS Spectrum imaging station and Luminescent Flux Values were obtained for each tumor. BLI flux values were used to randomize mice into four treatment groups (10 mice/group) based on BLI flux average signal. BLI was repeated 1-2x weekly during efficacy studies. Treatment groups included: Vehicle Control (0.5% w/v methylcellulose, 0.1% Tween-80 in ddH2O, mycophenolate mofetil (MMF) alone, RT alone, and MMF+RT. MMF (150mg/kg) was administered by oral gavage for 11 consecutive days starting between 13-18 days post-implantation. Mice were sedated using 2.5% isoflurane and RT was administered in 2Gy fractions on days 2, 4-5, 8-9, and 11 of MMF treatment using an Orthovoltage irradiator. Mice were euthanized upon development of neurological symptoms and survival data was analyzed using the Kaplan-Meier method using GraphPad Prism 10.0.

## Results

### The H3K27M mutation confers global changes to cellular metabolism

**Fig. 1:**
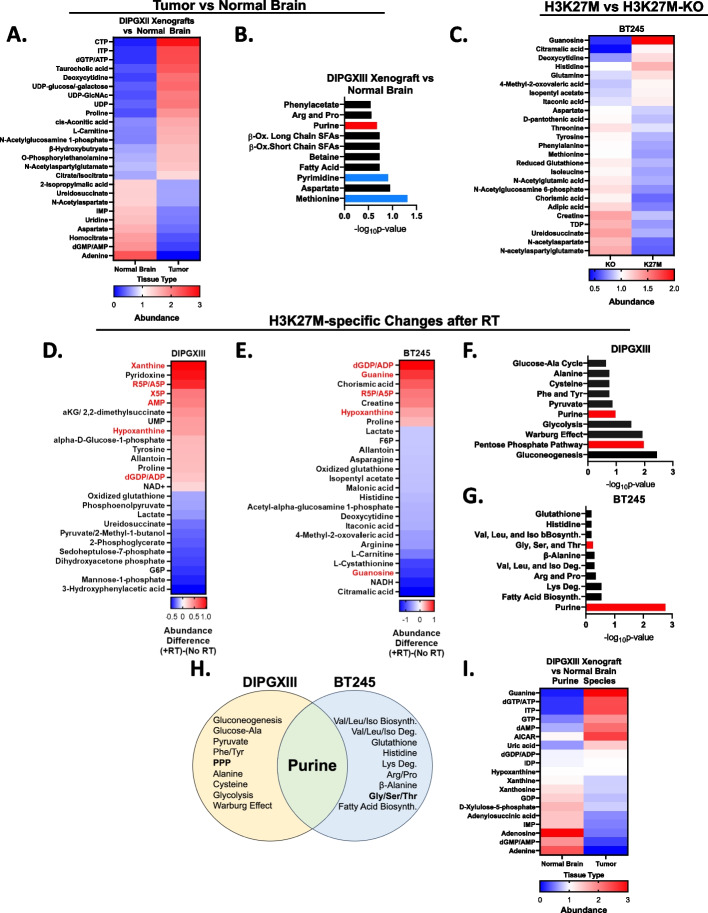
The H3K27M mutation influences metabolic phenotypes and the metabolic response to radiation. **A** Untreated intracranial DIPGXIII xenografts and contralateral normal brain issue (*n*=2 mice/group) were harvested, and their metabolites were collected by methanol extraction and measured using LC/MS. Data are presented as the top 25 significantly different metabolites between Tumor and NB and are ordered by difference in average median centered abundance between H3K27M and H3K27M-KO cells. **B** Metabolite set enrichment analysis of all metabolites whose abundance were significantly different between Tumor and normal brain (83 total). Blue bars indicate pathways important for DMG-H3K27M biology as reported in the literature. Red bars indicate pathways important for purine metabolism. **C** Metabolite levels in untreated BT245 isogenic cell lines as measured using LC/MS. Data represent the top 25 significantly different metabolites between H3K27M-KO and H3K27M cells. Data are ordered by difference in median centered abundance in descending order. Metabolite extractions were performed in triplicate. **D** and **E** H3K27M-isogenic cell lines DIPGXIII (**D**) and BT245 (**E**) were treated with or without 4Gy RT and harvested for metabolite quantification 2hrs later. Metabolites meeting the FC threshold were selected. Data represent the top 25 largest absolute post-RT abundance differences between H3K27M and H3K27M-KO cells in descending order. Red indicates abundances over a post-RT FC value of 0.0. Blue indicates abundances under a post-RT FC value of 0.0. **F** and **G** Metabolic set enrichment analysis was performed on the metabolite lists for DIPGXIII (**D**) and BT245 (**E**) and ordered based on –log_10_ *p*-value. Red bars indicate pathways important for purine metabolism. **H** Venn Diagram depicting the metabolic pathways enriched in RT-treated DMG-H3K27M isogenic cell lines. Bold-faced font indicates metabolic pathways important for purine metabolism and synthesis. **I** Purine metabolites from DIPGXIII xenograft tumors and contralateral normal brain from **A**) were compared and ordered by difference in average median centered abundance between tumor and normal brain

Altered cellular metabolism is a hallmark of cancer [33]. To begin to understand how the H3K27M mutation alters metabolism and facilitates treatment resistance, we quantified how metabolite levels differed between normal brain and DMG-H3K27M xenograft tumor tissue. Using fluorescence-guided microdissection, we separated GFP and luciferase-expressing DIPGXIII (DIPGXIII-GFP/LUC) orthotopic tumors from surrounding normal brain and quantified their metabolites. The brain-specific metabolite N-acetylaspartate was higher in cortex compared to DMG tumors, indicating that our fluorescent-based separation was successful (Fig. S1A). Numerous other metabolites including dGTP/ATP, citrate/isocitrate, and UDPglcNAC were elevated in DMG while adenine, ureidosuccinate, and aspartate were lower indicating that DMG tumor tissue possesses a distinct metabolome compared to normal brain (Fig. 1A). To understand the biology of these dysregulated metabolites, we performed metabolic pathway enrichment analysis and found enrichment of pyrimidine and methionine metabolism in DMG-H3K27M tumors (blue bars), consistent with recent reports of the importance of these pathways in H3K27M tumors, as well as purine metabolism (Fig. 1B) [25, 26, 30].

Some of these metabolic alterations might be caused specifically by the H3K27M mutation [24] while others might be related to alternative mutations or oncogenic transformation in general. To understand how the H3K27M mutation specifically affected metabolism, we used isogenic patient-derived DMG-H3K27M cell line pairs (DIPGXIII and BT245) in which the H3K27M mutation has been removed from the parental cell line using CRISPR/Cas9 (DIPGXIII-KO and BT245-KO) [20]. We confirmed the presence of the mutation in parental cells and the corresponding lack of H3K27me3 (Fig. S1B). Interestingly, proliferation was similar between unirradiated H3K27M expressing and H3K27M-KO models suggesting that intrinsic differences in metabolism are due to the mutation itself rather than differences in proliferation (Fig. S1C, D). We quantified metabolites in these isogenic pairs using LC/MS and found that H3K27M-expressing cells have an altered metabolome compared to H3K27M-KO counterparts (Fig. 1C, S2, and Supplemental Table 1). These findings are consistent with prior studies and confirm that the oncohistone plays an active role in altering DMG metabolism [24].

### RT induces changes in purine metabolism in H3K27M cells

We hypothesized that the H3K27M mutation influences the metabolic response to RT, thereby conferring RT resistance [34–36]. Using our H3K27M-isogenic models, we observed reduced viability in H3K27M-KO cells treated with RT compared to their H3K27M-expressing counterparts, suggesting that H3K27M-induced metabolic rewiring could be protective against RT (Fig. S1E, F).

We performed steady-state metabolomics on untreated and RT-treated H3K27M-isogenic cells two hours after RT to capture early shifts in cellular metabolism that might mediate RT resistance (Fig. 1D, E). We identified numerous metabolites that changed following RT in either the H3K27M or H3K27M-KO cells (Fig. 1D, E and Supplemental Table 2). Metabolites like glutamine in the DIPGXIII cells and aspartate in the BT245 model had similar responses to RT in both H3K27M and H3K27M-KO cells (Fig. S3A, B). Other metabolites like xanthine in the DIPGXIII model and dGDP/ADP in the BT245 model had responses to RT that varied depending on the presence of the H3K27M mutation (Fig. S3C, D, Supplemental Table 2).

To better understand which metabolic changes were H3K27M-specific, we calculated the differences in fold-change (FC) following RT between H3K27M and H3K27M-KO cell lines for each metabolite (Fig. 1D, E and Supplemental Table 2). We found that numerous purine species including xanthine, hypoxanthine, guanine, AMP, and ADP/dGDP responded differently to RT in H3K27M mutant cells compared to KO controls (Fig. 1D, E, Supplemental Table 2). Metabolite set enrichment analysis of the top 25 metabolites highlighted purine metabolism in both H3K27M-isogenic cell line pairs (Fig. 1F, G, H). Other purine metabolism-related pathways found included the pentose phosphate pathway (DIPGXIII) (Fig. 1F) that creates the ribose-5-phosphate (R5P) sugars needed for all nucleotides, and glycine/serine/threonine metabolism (BT245) that generates metabolites needed for purine ring construction (Fig. 1G). We reanalyzed DIPGXIII-GFP/LUC tumor data to focus on purine species and found that tumor tissue has an inverse purine metabolic phenotype to that of the normal tissue (Fig. 1I). Interestingly, our group has shown that purine metabolism mediates treatment resistance in adult glioblastoma (GBM), another form of astrocytoma [16, 37]. Together, these observations suggest that purine metabolism changes following RT in an H3K27M-specific fashion.

### Purine metabolic activity and enzyme expression are influenced by the H3K27M mutation

**Fig. 2:**
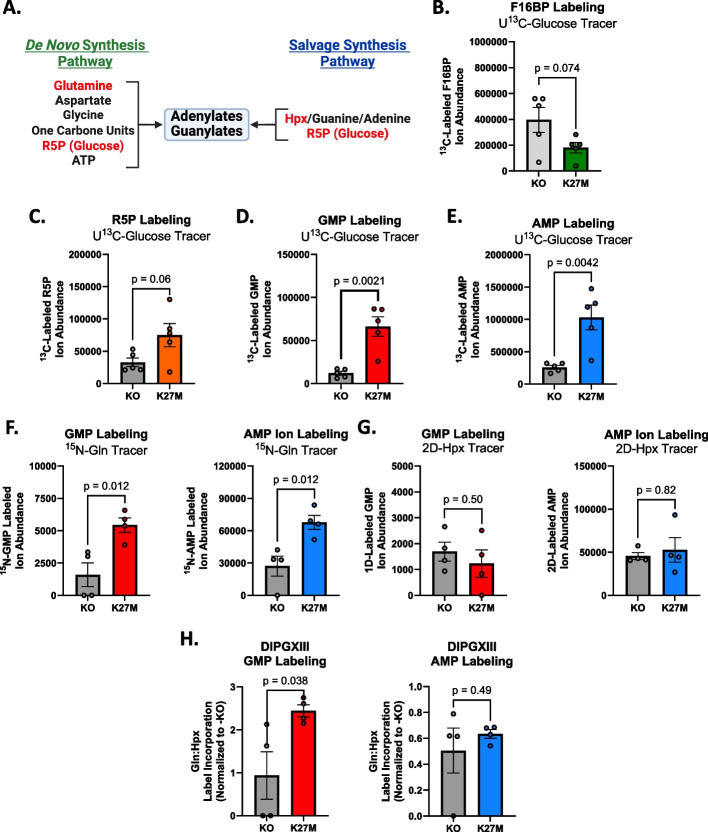
H3K27M cells have highly active purine synthesis. **A** Schematic of purine synthetic metabolic pathways showing where the purine *de novo* and salvage pathways converge to form mature purines. Schematic was created using BioRender.com. **B**-**E** Total abundance of ^13^C-labeled (**B**) F16BP, (**C**) R5P, (**D**) GMP, and (**E**) AMP in H3K27M cells when normalized to H3K27M-KO cells. **F** Total abundance of ^15^N-Gln-labeled GMP *(left)* and AMP *(right)* in H3K27M-isogenic cells. **H** Total abundance of deuterium-labeled GMP *(left)* and AMP *(right)* in H3K27M-isogenic cells. **H** Ratio of ^15^N-Gln:2D-Hpx labeled relative abundance of GMP *(left)* and AMP *(right)* in untreated DIPGXIII H3K27M-isogenic cell lines. Statistical analyses were performed using two-tailed t-tests in GraphPad Prism 10.0

We and others have found that purines, especially guanylates, contribute to treatment resistance in brain tumors [16, 37, 38]. We reasoned that targeting purine synthesis could increase RT efficacy in DMG-H3K27M. Purine nucleotides are produced through either the *de novo* synthesis (DNS) or salvage synthesis pathways (Fig. 2A, S4A). To determine which of these pathways DMG-H3K27M tumors may rely on, we performed stable isotope tracing to measure their baseline flux. This technique utilizes nutrients or metabolites carrying one or more nonradioactive atoms that are heavier than the naturally occurring form, allowing for their detection by mass spectrometry. Heavy isotope labeling patterns in metabolites formed from the tracer molecule then allows us to track how cells change their metabolic fluxes in response to RT via the measurement of labeled and unlabeled ions of a given metabolite, which reflects the volume of a given metabolic pathway, or through the relative percent abundance of labeled metabolite present which measures how much of a given metabolite pool is being synthesized from the tracer molecule of interest.

Purine synthesis, in most instances, requires glucose derived R5P upon which the purine ring is attached (Fig. 2A, S4A). Using universally labeled U^13^C-glucose tracer (Fig. S4B), we wanted to determine if H3K27M-expression was associated with increased glucose uptake and PPP activity as measured by tracer incorporation into fructose 1,6-bisphosphate (F16BP) and R5P, respectively. Here, we found that H3K27M and H3K27M-KO cells had similar relative percent abundances of labeled glucose-derived carbons in both metabolites (Fig. S4C, D). Interestingly, we found that H3K27M cells had significantly lower total F16BP and higher R5P ion abundances (Fig. 2B, C). Glucose metabolism starts with glucose being phosphorylated to create glucose-6-phosphate (G6P), which can then either proceed through glycolysis, or be shunted into the PPP where it forms the purine nucleotide building block R5P. These data suggest that while relative tracer abundances are similar between H3K27M and -KO cells, the total volume of glucose being shunted into R5P and purine production is higher in H3K27M cells. In support of this, we observed higher total purine synthesis in H3K27M cells characterized by greater ^13^C labeled ion abundance in AMP and GMP in H3K27M cells (Fig. 2D, E). However, the relative percent abundance of ^13^C tracer was higher in GMP and lower in AMP in H3K27M cells than in H3K27M-KO cells (Fig. S4E, F), suggesting that H3K27M cells prefer to use glucose-derived carbons for GMP synthesis. It cannot be discerned whether these labels are from newly synthesized purines or from labeled, salvaged ^13^C-R5P, as R5P is used in both DNS and salvage pathways. Together, these data suggest that H3K27M cells have a higher demand for total purine synthesis.

Next, we tracked both DNS and purine salvage, specifically, to determine pathway-specific differences in purine metabolism between H3K27M isogenic cell line models. We first measured DNS using ^15^N-glutamine as it is critical for the formation of newly synthesized purine rings (Fig. 2A, S5A). Purine salvage was separately measured using 2D-Hpx which is converted into the common purine precursor, IMP, which can then be converted into 1D-GMP or 2D-AMP (Fig. S5B). Prior to analysis, we observed identical label enrichment of glutamine and hypoxanthine in the respective tracer experiments in both H3K27M-isogenic lines (Fig. S5C, D). Here, we observed that H3K27M-expressing cells had significantly higher total abundance of ^15^N-labeled GMP and AMP than H3K27M-KO cells (Fig. 2F). This led to a trend towards higher relative abundance of higher *de novo* synthesis (DNS)-derived ^15^N label present in GMP, but not AMP in the mutant cells (Fig. S5E). Further, we observed roughly equal abundances of deuterium-labeled GMP and AMP between H3K27M-isogenic cells (Fig. 2G). However, H3K27M cells exhibited a trend towards lower relative abundance of 1D-labeled GMP than the knockout cells, suggesting that H3K27M-expressing cells prefer to create their GMP pools from DNS and not from salvage synthesis (Fig. S5F). We confirmed this using the ratio of the relative percent abundances of ^15^N-Gln- and 2D-Hpx-labeled GMP and AMP in the isogenic cell lines and found that H3K27M cells had a significantly higher ratio of ^15^N-Gln:2D-Hpx incorporation in GMP, but not AMP, than H3K27M-KO cells (Fig. 2H). Together, these results suggest that H3K27M cells activate purine synthesis and may prefer *de novo* synthesis rather than hypoxanthine salvage to form guanylates.

### De novo guanylate synthesis inhibition increases RT efficacy in H3K27M models

**Fig. 3:**
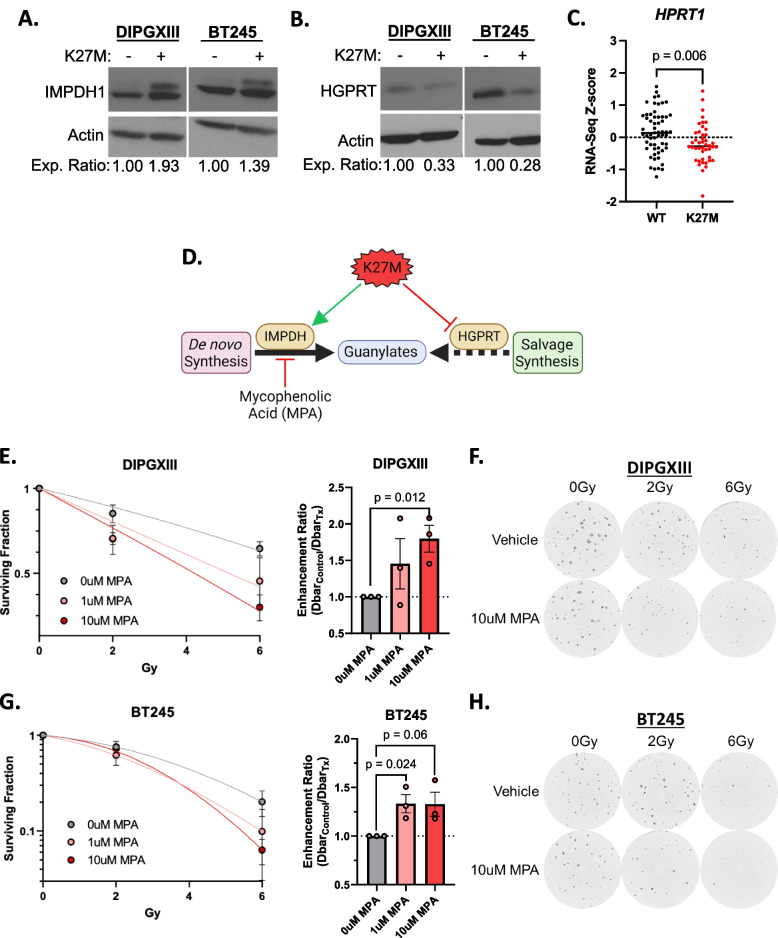
De novo purine synthesis inhibition radiosensitizes K27M cells. **A** and **B** Immunoblots of (**A**) IMPDH1 and (**B**) HGPRT expression in DMG-H3K27M isogenic paired cell lines. Densitometric values were calculated using ImageJ software and expression values were normalized to H3K27M-KO cells. Expression ratios are listed below the blot images. **C** Publicly available RNAseq Z-score data for *HPRT1* transcript expression from pediatric high-grade gliomas (pHGG) was obtained through PedCBioPortal and filtered to include only samples with known H3 mutational status (for both *H3F3A* and *HIST1H3B*) to include all known H3WT (*n*=59) and combined H3K27M (H3F3A-mut+*HIST1H3B-*mut) samples (*n*=44)*.* **D** Schematic depicting the hypothesis that the K27M mutation induces defective guanylate purine salvage through HGPRT suppression, leaving K27M cells vulnerable to *de novo* guanylate synthesis using IMPDH inhibition using MPA. Schematic was created using BioRender.com. **E** And **G** Long-term neurosphere growth assays for (**E**) DIPGXIII and (**G**) BT245 H3K27M-expressing cell lines treated with increasing doses of RT (0, 2, 6Gy) with or without 1-10μM MPA *(left)* and corresponding enhancement ratio (Dbar_control_/Dbar_Tx_) for each concentration of MPA *(right)* administered with RT. Each long-term neurosphere assay was performed 3x per cell line. Statistical analyses were performed using two-tailed t-tests in GraphPad Prism 10.0. **F** and **H** Live cell imaging analysis of (**F**) DIPGXIII and (**H**) BT245 neurospheres treated with 0-6Gy +/- 10μM MPA. Images taken 11 days (DIPGXIII) and 9 days (BT245) after replating in 96-well plates. Images acquired using a Cytation 5 plate reader and attached BioSpa incubator (Agilent Technologies)

Given the H3K27M-specific differences in GMP synthesis, we assessed the expression of the rate limiting enzymes in both *de novo* guanylate synthesis (IMPDH1 and IMPDH2) and guanylate salvage (HGPRT). We observed increased total expression of IMPDH1 protein (Fig. 3A) and decreased total expression of HGPRT (Fig. 3B) in H3K27M cells compared to H3K27M-KO. Using publicly available RNAseq data from patients bearing pediatric high-grade gliomas (pHGG) [2], we found that H3K27M pHGG tumors expressed less *HPRT1* transcript, which encodes HGPRT (Fig. 3C). We found no difference in *IMPDH1* or *IMPDH2* expression between H3WT or H3K27M pHGG tumors (Fig. S6A, B). We also did not observe a difference in the expression of *GMPS*, the gene-encoding the enzyme downstream of *IMPDH1/2* (Fig. S6C). Though there were some differences in the expression of enzymes used in adenylate synthesis in patient tumor samples, they do not correlate with our stable isotope tracing results (Fig. S6D, E). Lastly, we did not observe any discernable patterns in the expression of enzymes of the shared, upstream steps of DNS (Fig. S6F).

Increased reliance on *de novo* guanylate synthesis in H3K27M expressing cells suggests that inhibition of this pathway may have utility as monotherapy [39] or in combination with RT (Fig. 3D). Inhibition of *de novo* guanylate synthesis using the IMPDH inhibitor mycophenolic acid (MPA) radiosensitizes adult GBM brain tumors and is being evaluated in a Phase 0/1 clinical trial (NCT04477200) in human GBM patients [16, 40]. To explore a similar combination strategy in H3K27M cells, we treated DIPGXIII and BT245 patient-derived H3K27M-expressing cells with 0-10uM MPA in combination with increasing doses of RT (0-6Gy) to determine its effect on radiosensitivity. By measuring neurosphere formation over time, we observed dose-dependent decreases in the surviving fraction of DIPGXIII and BT245 neurospheres following RT which was augmented upon addition of MPA at either concentration (1μM and 10μM), leading to increased RT enhancement ratios (DIPGXIII: 1.45 and 1.78, BT245: 1.33 and 1.33) (Fig. 3E and G). This can be observed visually where we see that combined RT and MPA reduces neurosphere size and number in both of our H3K27M-expressing models, even at a low dosage of radiation (Fig. 3F and H). Others have observed single agent efficacy of MPA in DMG-H3K27M cells [41]. While we observed a significant reduction in total neurosphere number at endpoint in DIPGXIII cells at the highest concentration of MPA (Fig. S7A, B), we do not observe the same effect in BT245 cells (Fig. S7C, D), which could be due to shorter MPA exposure time reported here compared to previously reported findings [39]. Taken together, these results show that IMPDH inhibition might help overcome RT resistance in H3K27M-expressing cells.

**Fig. 4:**
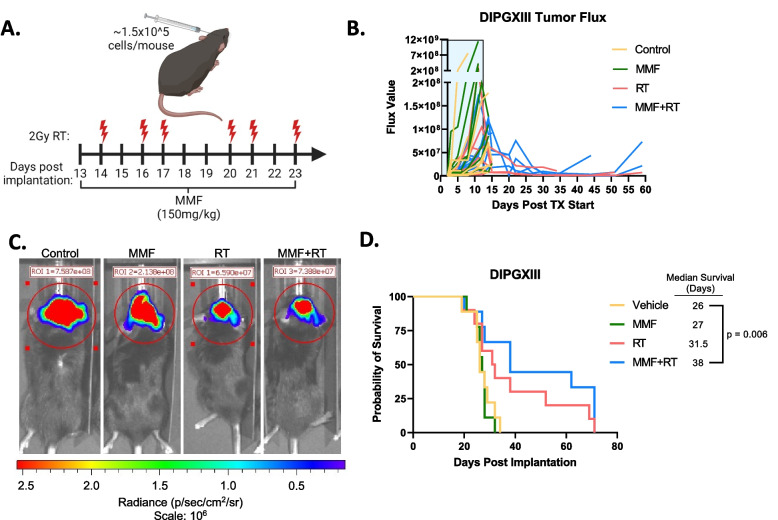
Combination RT and *de novo* purine synthesis inhibition reduces DMG-H3K27M tumor size and extends mouse survival in vivo. **A** Schematic of treatment schedule administered to DIPGXIII-bearing Rag1-KO mice. DIPGXIII cells expressing GFP and Luciferase (LUC) (DIPGXIII-GFP/LUC) were orthotopically implanted into the cortex and mice were administered 150mg/kg MMF for 11 days, with 6 intermittent doses of 2Gy RT (red bolts). Schematic was created using BioRender.com. **B** Spider plot of bioluminescent signal flux values for individual DIPGXIII-GFP/LUC mouse tumors. Blue box indicates the treatment period. **C** Representative bioluminescent signal images for one mouse per treatment group 24 days after implantation. **D** Kaplan-Meier survival analysis of Rag1-KO mice bearing treated and untreated DIPGXIII-GFP/LUC orthotopic xenograft tumors (*n*=10 mice/group). Statistical analysis was performed by comparing individual survival curves of individual treatment groups in GraphPad Prism 10.0

With these promising in vitro results, we next wanted to test the in vivo efficacy of RT in combination with IMPDH inhibition in H3K27M expressing tumors. Previous work from our group and others has shown that mycophenolate mofetil (MMF), the pro-drug of MPA, has efficacy in intracranial adult GBM models [16] and we sought to employ a similar strategy here. DIPGXIII-GFP/LUC cells were orthotopically implanted into the cortex and monitored by bioluminescent imaging (BLI). Once tumors were detectable, mice were randomized and treated with RT alone, MMF alone, combined RT and MMF, or vehicle control (Fig. 4A). Mouse weight was largely unaffected by the treatment course (Fig. S8). Unlike our in vitro experiments, MMF alone had a no effect on tumor size measured by BLI (Fig. 4B). Both RT and MMF+RT decreased tumor bioluminescence, but tumors eventually regrew, and we did not observe marked difference between the two groups (Fig. 4B, C). MMF alone had no effect on median survival versus vehicle controls (27d vs 26d, respectively, *p*=0.6). RT alone increased mouse survival over vehicle control (31.5d), but not in a statistically significant manner. Combination MMF+RT significantly extended survival over vehicle controls (38d vs 26d, *p*=0.006), but did not cure tumors (Fig. 4D). These findings suggest that while combination MMF+RT treatment extends survival, there may be resistance mechanisms employed by DMG-H3K27M tumors to evade MMF+RT treatment.

### Radiation therapy induces limited changes to purine metabolism in H3K27M cells

**Fig. 5:**
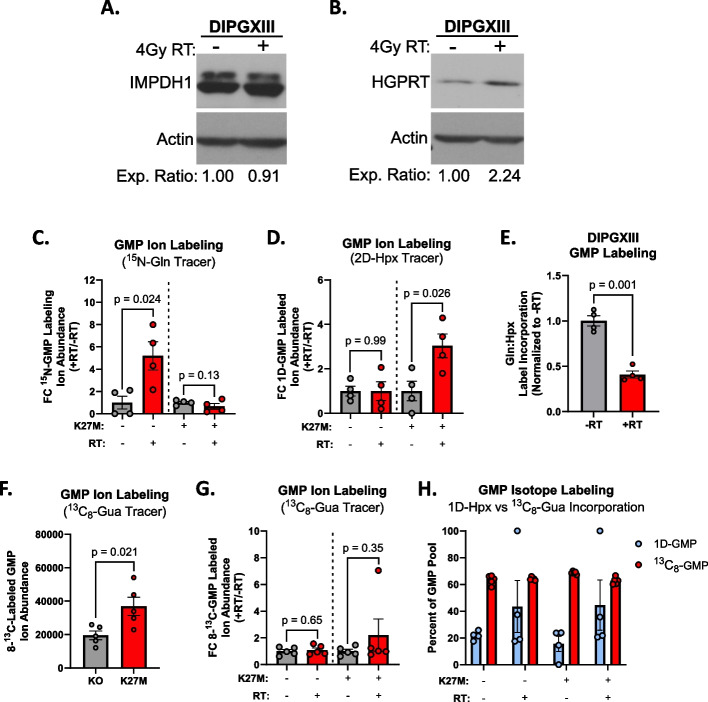
H3K27M-expressing cells increase hypoxanthine-derived salvage and maintain high guanine salvage following RT. **A** and **B** Immunoblots of (**A**) IMPDH1 and (**B**) HGPRT expression before and after 4Gy RT in DIPGXIII cells. Densitometric analysis was performed using ImageJ software and expression values were normalized to No-RT controls. Values are listed below the blot images. **C** FC in the ion abundances of ^15^N-labeled GMP in DIPGXIII H3K27M-isogenic cell lines before (gray bars) and 3hrs after 4Gy RT (red bars). Data are normalized to untreated control samples. **D** FC in the ion abundances of 1D-labeled GMP in DIPGXIII H3K27M-isogenic cell lines before (gray bars) and 3hrs after 4Gy RT (red bars). Data are normalized to untreated control samples. **E** Ratio of ^15^N-Gln:2D-Hpx label incorporation into GMP after 3 hours in DIPGXIII cells that were either untreated or treated with 4Gy RT. Data is normalized to untreated control. **F** Total abundance of ^13^C_8_-labeled GMP in H3K27M-isogenic cells. **G** FC in the ion abundances of ^13^C_8_-labeled GMP in DIPGXIII H3K27M-isogenic cell lines before (gray bars) and 3hrs after 4Gy RT (red bars). **H** Percent relative abundances of 1D-labeled GMP (light blue bars) and ^13^C_8_-labeled GMP (red bars) in H3K27M-isogenic cells before and after 3hrs 4Gy RT. Statistical analyses were performed using two-tailed t-tests in GraphPad Prism 10.0

To understand the discrepancy observed between strong in vitro results using MPA+RT and the limited efficacy of MMF+RT in vivo, we first examined the expression of the rate limiting enzymes in the guanylate synthetic pathways as potential mechanisms of adaptation. Here, H3K27M cells did not show increased IMPDH1 protein expression following RT (Fig. 5A). However, RT increased HGPRT expression in H3K27M-expressing DIPGXIII cells (Fig. 5B). This could mean that while H3K27M cells appear to rely on MPA-sensitive DNS in the unperturbed state, they upregulate MPA-resistant purine salvage synthesis following RT, which may mediate resistance to DNS inhibitors like MMF in vivo*.*

To evaluate this, we employed stable isotope tracing to measure the activity of total, *de novo*, and salvage purine synthesis following RT using U^13^C-glucose, ^15^N-Gln, and 2D-Hpx as previously described (Fig. 5, S9-10). Using U^13^C-glucose, we first determined that glucose uptake and PPP activity did not change following RT as measured by both fold change (FC) in relative abundance (Fig. S10A, C) and FC in ion abundance compared to unirradiated controls for both unlabeled and labeled F16BP and R5P ions (Fig. S10B, D). These data suggest that both H3K27M and H3K27M-KO cells maintain similar demand for glucose and PPP-derived R5P following RT.

Next, we examined the effect of RT on *de novo* synthesis (DNS) using a ^15^N-glutamine tracer. We again observe equal enrichment of the ^15^N-Gln and 2D-Hpx tracer molecules in both H3K27M and H3K27M-KO cells (Fig. S10A, G). We showed previously that despite high total abundances of ^15^N label in both purines in H3K27M cells, mutant cells converted a higher relative amount of glutamine-derived ^15^N label into GMP, but not AMP, than H3K27M-KO cells in unirradiated samples (Fig. 2E, S5E). Following RT, H3K27M-KO cells increased glutamine-derived ^15^N labeling in both total ion and relative percent abundances of GMP, while similar increases were not seen in H3K27M expressing cells (Fig. 5C, S10B). Further, there was a trend towards a lower relative percent abundance of glutamine-derived ^15^N labeling of GMP in irradiated H3K27M cells compared to knockout counterparts (Fig. S10C). These findings suggest that cells lacking the H3K27M mutation upregulate *de novo* guanylate synthesis following RT, but cells expressing the H3K27M mutation do not. These changes were guanylate specific, as glutamine-driven AMP synthesis did not increase in either H3K27M expressing or H3K27M KO cells (Fig. S10D-F).

We utilized a 2D-hypoxanthine (2D-Hpx) tracer to interrogate purine salvage after RT. Unexpectedly, H3K27M cells increased the abundance of 2D-Hpx-labeled GMP following RT while H3K27M-KO cells do not (Fig. 5D). Both cell lines show trends towards increased relative abundance of 2D-Hpx salvage into GMP post-RT to a similar degree (Fig. S10H, I). These changes suggest increased hypoxanthine-driven guanylate salvage following RT in H3K27M expressing cells, which when combined with increased glutamine-driven GMP synthesis following RT in H3K27M-KO cells, suggests a decreased reliance on *de novo* GMP synthesis following RT in H3K27M cells (Fig. 5E). There was no meaningful change in hypoxanthine-derived AMP ion abundance or percentage labeling in either H3K27M or H3K27M-KO cells (Fig. S10 J-L).

While increased hypoxanthine salvage after RT in H3K27M expressing cells would be interesting, it would be unlikely to account for MMF resistance, as hypoxanthine-mediated GMP synthesis still requires the activity of the MMF-target IMPDH. GMP can also be formed from the IMPDH-independent salvage of guanine, which would bypass the effect of MPA/MMF. To assess the activity of guanine salvage, we traced irradiated and unirradiated H3K27M and H3K27M-KO cells with ^13^C_8_-guanine to determine if GMP cells could salvage free guanine bases into GMP (Fig. S11A). Here, we observed that H3K27M-expressing and -KO cells had similar relative enrichment of guanine tracer into GMP prior to irradiation (Fig. S11B). In unirradiated samples, H3K27M-expressing cells had nearly double the number of ^13^C_8_-labeled GMP ions, suggesting that H3K27M cells have higher total volume of guanine salvage into GMP than H3K27M-KO cells (5F). Interestingly, the total ion abundances do not appear to increase following irradiation, and we see that both isogenic lines have similar relative abundances of ^13^C_8_-GMP following RT (Fig. 5G, S11C). Interestingly, ^13^C_8_-GMP relative abundance remained high following RT in both cell lines (>60% in all conditions), in contrast to 2D-Hpx label incorporation (~20% before RT, ~40% after RT) that we previously observed (Fig. 5H). This suggested that free guanine could contribute a significant fraction of GMP in DMG cells and in an IMPDH-independent fashion and that RT-induced increases in hypoxanthine salvage may serve as a supplementary source of GMP to meet cellular demands.

### Guanine salvage bypasses IMPDH inhibition and reverses radiosensitization

**Fig. 6:**
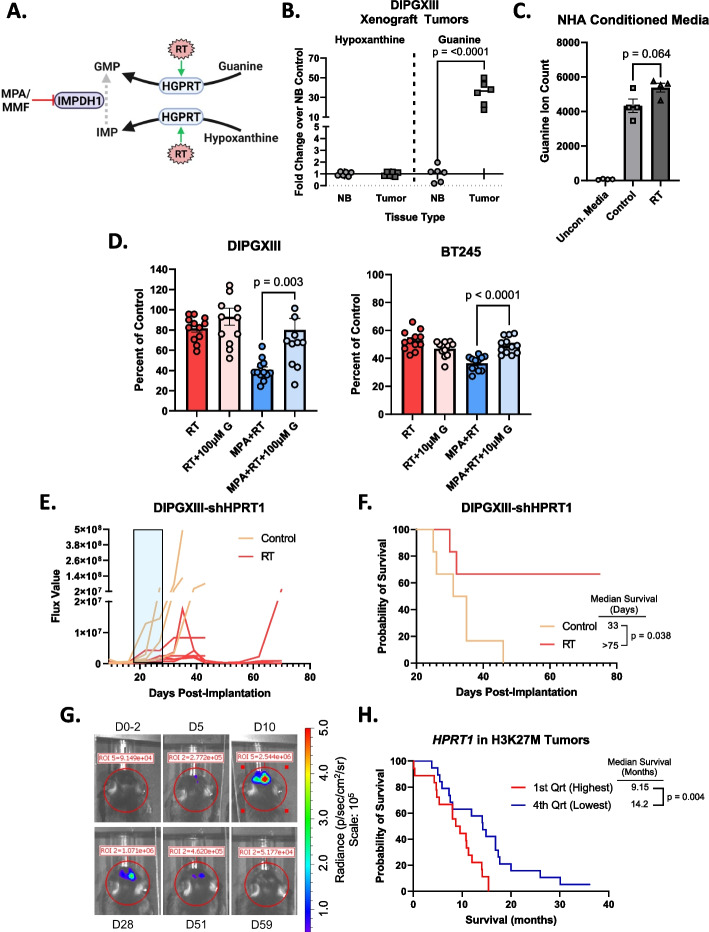
HGPRT-mediated guanine salvage in H3K27M-expressing tumors protects against the effects of RT. **A** Schematic showing mechanism of in vivo resistance to MMF via increased intratumoral guanine abundance. Schematic was created using BioRender.com. **B** Hypoxanthine and guanine abundances in orthotopic DIPGXIII-GFP/LUC xenograft tumors and contralateral normal brain tissue. Tissues were harvested by GFP fluorescence-guided mechanical resection, and their metabolites collected via methanol extraction before LC/MS. Data is normalized to normal brain (NB). **C** Guanine ion abundance in NHA-conditioned media collected 24hrs after a single 4Gy dose of RT. **D** Endpoint neurosphere counts of DIPGXIII *(left)* and BT245 *(right)* treated with RT or MPA+RT in TSM media with or without supplemented guanine (+G). DIPGXIII cells were given 100µM guanine. BT245 cells were given 10µM guanine. **E** Spider plot of bioluminescent signal flux values for individual DIPGXIII-GFP/LUC-shHPRT1 mouse tumors. Blue box indicates the treatment period. **F** Kaplan-Meier survival analysis of Rag1-KO mice bearing DIPGXIII-GFP/LUC-shHPRT1 orthotopic xenograft tumors given vehicle control or RT (*n*=7 mice/group). Statistical analysis was performed by comparing individual survival curves using GraphPad Prism 10.0 software. **G** Representative bioluminescent imaging of an RT-treated mouse bearing an orthotopic DIPGXIII-GFP/LUC-shHPRT1 xenograft tumor that experienced a complete response following treatment. D# indicates the day at which the image was taken with respect to the end of the treatment regimen. The first image was taken two days prior to the completion of the treatment regimen (D0-2). **H** Kaplan-Meier survival analysis of patient H3K27M-expressing tumors based on *HPRT1* expression where the 1^st^ quartile represents the highest *HPRT1* expression, and the 4^th^ quartile represents the lowest *HPRT1* expression

HGPRT can salvage both hypoxanthine and guanine (Fig. 6A). Salvaged hypoxanthine is converted to IMP whose conversion into GMP is blocked by MPA/MMF. Salvaged guanine, by contrast, directly forms GMP and bypasses IMPDH inhibition, potentially leading to MPA/MMF resistance in our models.

Our initial neurosphere assays (Fig. 3) were performed in TSM media where hypoxanthine is the dominant purine base available [42]. However, the mouse tumor microenvironment (TME) is vastly different than the cell culture dish regarding metabolite availability. To determine which bases were available for guanylate salvage in DMG-H3K27M tumors, we analyzed both guanine and hypoxanthine levels in orthotopic DIPGXIII-GFP/LUC tumors and contralateral cortex. While DIPGXIII-GFP/LUC tumors and normal brain contained roughly the same abundance of hypoxanthine, tumor tissue possessed a 34-fold higher abundance of guanine (Fig. 6B).

Others have found that normal human astrocytes (NHAs) secrete salvageable guanine bases into the cell culture media [39, 43] and our studies agree (Fig. 6C). Interestingly, we observed a strong trend towards increased guanine secretion following RT (Fig. 6C). This suggests that these cells can support the DMG-H3K27M cells following treatment, potentially promoting RT resistance. To test this, we repeated our long-term NSAs in H3K27M cells cultured in TSM media supplemented with extracellular guanine. We observed that the addition of guanine reduced the effect of combination MPA+RT, suggesting that guanine salvage can be utilized by DMG-H3K27M to resist DNS inhibition and RT (Fig. 6D).

Lastly, to determine if the H3K27M mutation affects the cellular capacity for purine uptake from the environment, we examined the expression of several equilibrative nucleoside transporters (ENTs) in our H3K27M-isogenic models. These transport proteins facilitate the uptake of several purine species, including adenine and guanine. Here, we observed higher expression of ENT1 and lower expression of ENT4 in both DIPGXIII and BT245 parental cells, suggesting a role for the H3K27M mutation in influencing their expression which requires further investigation (Fig. S12). We did not observe any H3K27M-specific expression patterns for ENT2, and we did not observe any RT-induced expression changes in ENT expression. Due to the promiscuous nature of ENTs, more investigation would be required to determine which of these transporters facilitates guanine uptake in H3K27M cells.

Together, these data indicate that guanine salvage substrates, perhaps from non-malignant cells in the TME, could be an important mediator of treatment resistance in DMG-H3K27M tumors.

### HPRT1 silencing extends survival in mice bearing DMG-H3K27M xenografts

We found that DMG-H3K27M cells have highly active purine salvage synthesis, particularly of guanylates, which can bypass MPA/MMF treatment in irradiated cells to promote treatment resistance. We then wanted to determine if direct HGPRT inhibition could increase RT efficacy in DMG-H3K27M xenograft tumors. No blood-brain barrier (BBB)-penetrant HGPRT inhibitors currently exist, so we utilized a pooled shRNA to knockdown *HPRT1* expression in DIPGXIII-GFP/LUC cells (DIPGXIII-GFP/LUC-shHPRT1) (Fig. S13A). These cells were then implanted into the cortices of Rag1-KO mice. Tumor-bearing mice were RT-treated as previously described (Fig. 4A). Mouse weight was largely unaffected by the treatment regimen (Fig. S13B). RT alone greatly reduced BLI signal in mice bearing DIPGXIII-GFP/LUC-shHPRT1 tumors (Fig. 6E). Median survival in DIPGXIII-GFP/LUC-shHPRT1 tumors was similar to vehicle control mice in our first experiment (33d vs 26d), suggesting that purine salvage is dispensable for initial tumor growth (Figs. 4D and 6F). However, irradiation of HGPRT-deficient tumors significantly extended survival (>75d) and led to multiple complete responses (Fig. 6E-G). Using publicly available patient tumor data, we found that H3K27M tumors had an inverse correlation between patient outcome and *HPRT1* expression (Fig. 6G) [2]. We did not observe an inverse correlation between *IMPDH1* expression and median survival but did for *IMPDH2* (Fig. S14). Interestingly, *IMPDH1* has been shown to be ubiquitously expressed in many different tissues, while *IMPDH2* is upregulated in proliferating tissues, which could include DMG tumor tissue [44]. Together, these findings indicate that purine salvage through HGPRT may mediate RT resistance in DMG-H3K27M.

## Discussion

In this study, we have defined new metabolic strategies to overcome treatment resistance in devastating DMG-H3K27M tumors. Using steady-state and stable isotope tracing metabolomics in patient-derived H3K27M-isogenic cell lines, we found that H3K27M cells preferentially rely on the *de novo* synthesis (DNS) of guanylates. This activity is likely due to low HGPRT and high IMPDH1 expression in H3K27M cells. Inhibiting DNS of guanylates potentiated the effects of RT on H3K27M models in vitro and in vivo but did not cure tumors. We found that H3K27M cells increase hypoxanthine-derived guanylate salvage to a limited degree in response to RT, which is blocked by IMPDH inhibition. Interestingly, we found that both H3K27M and H3K27M-KO cells, regardless of treatment status, maintain high levels of guanine salvage. Further, we found that DMG-H3K27M xenografts had abundant intratumoral guanine that is likely secreted, in part, by normal astrocytic cells in the TME. This IMPDH-independent guanine salvage, fueled by an abundance of free guanine bases, bypasses the mechanism of action of MPA/MMF and promotes RT resistance. These data suggested that guanylate salvage activity may be a key mechanism of RT resistance in DMG. Consistent with this model, inhibition of guanylate salvage in DMG-H3K27M tumors overcame RT resistance and led to complete responses in several mice. Together, our findings suggest that while DMG-H3K27M tumors rely on both *de novo* and salvage guanylate synthesis, salvage synthesis of guanylates preferentially contributes to treatment resistance.

Our findings add to the growing body of work showing that altered metabolism is a targetable vulnerability in DMG. These tumors cells preferentially rely on *de novo* pyrimidine synthesis and methionine consumption [25, 26]. Like others [41], we have found that DMG-H3K27M tumors also rely on *de novo* guanylate synthesis for survival. However, none of these seminal findings have considered how H3K27M-mediated metabolic changes are related to RT resistance. Here, we unexpectedly discovered that the highly active guanylate salvage pathway in DMG-H3K27M cells may be a key mechanism to resist RT. Thus, our work suggests that while strategies targeting methionine, pyrimidine, or *de novo* guanylate synthetic metabolism may be effective as monotherapies, targeting guanylate salvage in combination with RT may be an effective combination strategy to combat DMG-H3K27M tumors.

Our discoveries have important implications for the treatment of DMG-H3K27M patients. Nearly every patient with DMG-H3K27M receives RT, so a drug that overcomes DMG-H3K27M RT resistance by inhibiting guanylate salvage could immediately be evaluated in combination with RT in patients. In this study, we utilized a genetic approach to inhibit guanylate salvage, which cannot readily be translated to patients. 6-mercaptopurine (6-MP) can compete with HGPRT-mediated guanine and hypoxanthine salvage and has clinical and preclinical efficacy in a variety of cancers [43, 45]. However, 6-MP has poor BBB penetrance in preclinical studies, suggesting it will be ineffective for gliomas [45]. Acyclic nucleoside phosphonates (ANPs) are being investigated as antimalarial compounds that target *Plasmodium spp.* HGPRT/HG(X)PRT [46]. These ANPs complex with human HGPRT, however it is unknown if they can cross the blood-brain barrier. Preclinical studies to assess the pharmacokinetics of molecules like ANPs and drug discovery directed at improving the BBB penetrance of other inhibitors like 6-MP or development of new molecules could yield new strategies to target guanylate salvage in DMG-H3K27M tumors.

These studies present lingering questions that still need to be answered. While we know that RT induces HGPRT expression, we do not know the molecular mechanism behind this upregulation. The rapid change in HGPRT expression (<3hrs) suggests an epigenetic mechanism that could be related to the underlying H3K27M mutation*.* Additionally, we do not know if the ratio of DNS to either form of salvage synthesis changes further as time progresses. We also need to confirm the source of the abundant guanine in our xenograft model, although our in vitro studies suggest non-cancerous cells such as astrocytes may be a source [41, 47]. Lastly, we do not know if the therapeutic window for inhibiting guanylate salvage will be large enough between normal brain and DMG-H3K27M tissue to improve treatment efficacy. The normal brain salvages purines, and HGPRT loss underlies Lesch-Nyan disease. It is possible that temporary inhibition of HGPRT in normal tissues may be well-tolerated when combined with radiation [48].

## Conclusions

Together, our studies suggest that multiple routes of purine synthesis are important for DMG growth and treatment resistance. Combining *de novo* purine synthesis inhibition with RT, an approach that we are utilizing clinically for adult patients with GBM [40], may be less promising in DMG-H3K27M due to high rates of purine salvage. Further preclinical study is needed to determine if combining small molecule inhibition of purine salvage with radiation can improve RT efficacy and be translated to clinical trials to improve DMG-H3K27M patient outcomes.

### Supplementary Information

**Additional file 1:** **Supplemental Figure 1.** Patient-derived DMG-H3K27M isogenic cell lines and tumors represent the appropriate biology. *A.)* Quantification of N-acetylaspartate in normal brain vs DIPGXIII xenograft tumor tissue. Statistical analysis was performed using a two-tailed t-test. *B.) *Immunoblot analysis for H3K27M expression and corresponding H3K27me3 signal in patient-derived DMG-H3K27M isogenic models. *C.) and D.)* Normalized endpoint CellTiter-Glo 3D luminescence values representing the abundance of DIPGXIII *(C.)*and BT245 *(D.)* H3K27M-isogenic cells after 7 days of growth. *E.) and F.)* Radiation response CellTiter-Glo 3D viability assay curves for DIPGXIII *(E.) *and BT245 *(F.)* H3K27M-isogenice cell line pairs normalized to 0Gy control. 

**Additional file 2: Supplemental Figure 2.** The H3K27M mutation in DIPGXIII cells facilitates altered metabolism. Metabolite levels in untreated DIPGXIII H3K27M-isogenic cell lines as measured using LC/MS. Data represent the top 25 significantly different metabolites between H3K27M-KO and H3K27M cells. Data are ordered by difference in average median centered abundance in descending order. Metabolite extractions were performed in triplicate.

**Additional file 3: Supplemental Table 1.** Global median centered abundances for the top 25 metabolites at baseline between H3K27M-isogenic cell line pairs. Metabolite name and average GMCA in H3K27M-KO and H3K27M DIPGXIII and BT245 cell line pairs and difference in abundance between H3K27M and H3K27M-KO.

**Additional file 4: Supplemental Table 2.** Post-RT FC for the top 25 metabolites between H3K27M-isogenic cell line pairs. Metabolite name and average post-RT FC in H3K27M-KO and H3K27M DIPGXIII and BT245 cell line pairs and difference in abundance between H3K27M and H3K27M-KO values. Purine metabolites denoted by an asterisk (*). 

**Additional file 5: Supplemental Figure 3.** Radiation-induced metabolite abundance changes vary. *A.) and B.) *Quantification of representative metabolites glutamine in DIPGXIII cells *(A.) *and aspartate in BT245 cells (*B.)* whose RT-induced abundance changes were similar despite the presence or absence of the H3K27M mutation. *C.) and D.)* Quantification of representative metabolites xanthine in DIPGXIII cells *(C.)* and dGDP/ADP in BT245 cells *(D.)* whose RT-induced abundance varied based on the expression of the H3K27M mutation. Statistical analyses were performed using a two-tailed t-test in GraphPad Prism 10.0.

**Additional file 6: Supplemental Figure 4.** Stable isotope tracing of U^13^C-Glucose uptake and usage in purine synthesis. *A.) *Schematic of glucose metabolism through glycolysis and the Pentose Phosphate Pathway shunt towards purine synthetic pathways. Schematic was created using BioRender.com. *B.) *Relative enrichment of U^13^C-Glucose-derived label in F16BP pools in unirradiated DIPGXIII H3K27M-isogenic cells. *C.-G)* Relative abundance of^13^C-labeled *(C.) *F16BP, *(D.) *R5P, *(E.) *IMP, *(F.)*AMP, and *(G.) *GMP in DIPGXIII H3K27M-isogenic cells. Data are normalized to H3K27M-KO samples. Statistical analyses were performed using two-tailed t-tests in GraphPad Prism 10.0.

**Additional file 7: Supplemental Figure 5.** Stable isotope tracing of *de novo *and salvage purine synthesis. A*.) *and *B.) *Schematics for *(A.) *^15^N-Gln tracing and *(B.) *2D-Hpx and the respective labeling pattern of each purine metabolite. Schematics were created using BioRender.com. *C.) and D.) *Percent of tracer metabolite enrichment in *(C.)*^15^N-Gln and *(D.) *2D-Hpx pools in unirradiated DIPGXIII H3K27M-isogenic cells. *E.)* FC in relative ^15^N-labeled GMP*(left) *and AMP *(right)* abundance between irradiated H3K27M-isogenic cell lines. Data are normalized to H3K27M-KO samples. *F.) *FC in relative deuterium-labeled GMP *(left) *andAMP *(right)* abundance between irradiated H3K27M-isogenic cell lines. Data are normalized to H3K27M-KO samples. Statistical analyses were performed using two-tailed t-tests in GraphPad Prism 10.0.

**Additional file 8: Supplemental Figure 6.** Purine metabolic enzyme expression. *A-E.)* Publicly available RNAseq Z-score data for *(A.) IMPDH1, (B.) IMPDH2, (C.) GMPS, (D.) ADSS, *and *(E.) APRT *transcript expression from pediatric high-grade gliomas (pHGG) was obtained through PedCBioPortal and filtered to include only samples with known H3 mutational status (for both *H3F3A* and *HIST1H3B*) to include all known H3WT (n=59) and combined H3K27M (H3F3A-mut+*HIST1H3B-*mut) samples (n=44). *F.) *RNAseq Z-score data for all common *de novo *purine synthesis enzymes in H3WT or H3K27M-mutant pHGG tumors. Statistical analyses were performed using two-tailed t-tests in GraphPad Prism 10.0.

**Additional file 9: Supplemental Figure 7.** Single-agent mycophenolic acid has mixed efficacy *in vitro*. *A.) and C.) *Endpoint neurosphere counts of DIPGXIII *(A.)* and BT245* (C.)* cells treated with increasing concentrations of MPA (0-10mM) during the long-term neurosphere growth assays. *B.) and D.)* Long-term growth curves over time of DIPXIII *(B.)* and BT245 *(D.)* cells treated with increasing concentrations of MPA (0-10mM). Endpoint and growth curve neurosphere data was acquired using a Cytation 5 plate reader and attached BioSpa incubator (Agilent Technologies).

**Additional file 10: Supplemental Figure 8.** DIPGXIII-LUC/GFP tumor-bearing Rag1-KO mouse weights are minimally affected after treatment course. Normalized measurement of mouse weight over treatment time course for DIPGXIII-GFP/LUC xenograft tumor-bearing mice. Blue box indicates treatment period.

**Additional file 11: Supplemental Figure 9.** U^13^C-Glucose tracing of glucose uptake and PPP activity following RT. *A.) and C.) *FC in ^13^C label enrichment in *(A.) *F16BP and *(C.) *R5Pin H3K27M-isogenic cell lines 3hrs after 4Gy single dose RT. RT conditions for each cell line are normalized to the respective 0Gy control. *B.) and D.) *FC of unlabeled and ^13^C-labeled ion abundances following RT for *(B.)*F16BP and *(D.) *R5P in H3K27M-isogenic cell lines following irradiation with a single 4Gy dose of RT. Statistical analyses were performed using two-tailed t-tests in GraphPad Prism 10.0.

**Additional file 12: Supplemental Figure 10.**^15^N-Gln and 2D-Hpx Isotope tracing of purine synthesis following RT. *A.) *Percent of tracer metabolite enrichment of ^15^N-Gln in H3K27M-isogenic cells 3hrs after 4Gy RT. *B.) *FC in ^15^N label enrichment in GMP in H3K27M-isogenic cell lines 3hrs after 4Gy single dose RT. RT conditions for each cell line are normalized to the respective 0Gy control. *C.) *FC in relative ^15^N-labeled GMP abundance between irradiated H3K27M-isogenic cell lines. Data are normalized to H3K27M-KO samples. *D.) *FC in ^15^N-labeled AMP ion abundance 3hrs after 4Gy single dose RT in H3K27M-isogenic cell lines. RT-treated samples were normalized to their respective unirradiated controls. *E.) *FC in the relative abundance of ^15^N-labeled AMP in DIPGXIII H3K27M-isogenic cell lines before (gray bars) and 3hrs after 4Gy RT (blue bars). Data are normalized to untreated control samples. *F.) *FC in relative ^15^N-labeled AMP abundance between irradiated H3K27M-isogenic cell lines.Data are normalized to H3K27M-KO samples. *G.)*Percent of tracer metabolite enrichment of 2D-Hpx in H3K27M-isogenic cells 3hrs after 4Gy RT. *H.) *FC in deuterium label enrichment in GMP in H3K27M-isogenic cell lines 3hrs after 4Gy single dose RT. RT conditions for each cell line are normalized to the respective 0Gy control. *I.) *FC in relative deuterium-labeled GMP abundance between irradiated H3K27M-isogenic cell lines. Data are normalized to H3K27M-KO samples. *J.) *FC in 2D-labeled AMP ion abundance 3hrs after 4Gy single dose RT in H3K27M-isogenic cell lines. RT-treated samples were normalized to their respective unirradiated controls.* K.) *FC in the relative abundance of 2D-labeled AMP in DIPGXIII H3K27M-isogenic cell lines before (gray bars) and 3hrs after 4Gy RT (blue bars). Data are normalized to untreated control samples.* L.) *FC in relative 2D-labeled AMP abundance between irradiated H3K27M-isogenic cell lines. Data are normalized to H3K27M-KO samples. Statistical analyses were performed using two-tailed t-tests in GraphPad Prism 10.0.

**Additional file 13: Supplemental Figure 11.**^13^C_8_-guanine tracing of purine salvage before and after RT. *A.) *Percent enrichment of ^13^C_8_-guanine tracer in DIPGXIII H3K27M-isogenic cell lines before and 3hrs after 4Gy RT. *B.)*FC in relative abundance of ^13^C_8_-labeled GMP in unirradiated DIPGXIII H3K27M-isogenic cell lines. Data are normalized to H3K27M-KO samples.* C.)* FC in relative abundance of ^13^C_8_--labeled GMP between DIPGXIII H3K27M-isogenic cell lines 3hrs after 4Gy single dose RT. Data are normalized to H3K27M-KO samples. Statistical analyses were performed using two-tailed t-tests in GraphPad Prism 10.0.

**Additional file 14: Supplemental Figure 12.** RT does not induce changes in ENT expression. Immunoblot analysis of ENTs 1, 2, and 4 in DIPGXIII and BT245 H3K27M-isogenic cell lines before and 3hr after 4Gy RT.

**Additional file 15: Supplemental Figure 13.** DIPGXIII-LUC/GFP-shHPRT1 tumor-bearing Rag1-KO mouse weights are minimally affected after RT. *A.) *Immunoblot of control DIPGXIII and DIPGXIII-shHPRT1 cells probed for HGPRT expression. *B.) *Normalized measurement of mouse weight over treatment time course for DIPGXIII-GFP/LUC-shHPRT1 xenograft tumor-bearing mice. Blue box indicates treatmentperiod.

**Additional file 16: Supplemental Figure 14.***IMPDH1/2* expression in H3K27M tumors. *A.) and B.) *Kaplan-Meier survival analysis of patient H3K27M-expressing tumors based on *IMPDH1 (A.) and IMPDH2 (B.) *expression where the 1^st^ quartile represents the highest expression, and the 4^th^ quartile represents the lowest expression.

**Additional file 17.**

## Data Availability

Human tumor RNAseq Z score data and patient survival data were obtained from the Institute of Cancer Research (London, UK) using PedcBioPortal (Children’s Hospital of Philadelphia Research Initiative, https://pedcbioportal.kidsfirstdrc.org/) where it is currently available [2, 31, 32]. All other data supporting the findings of this study can be made available upon request to the authors.

## Abbreviations

DMG: Diffuse midline glioma
DIPG: Diffuse intrinsic pontine glioma
H3K27M: Histone H3, K27M mutant
RT: Radiation therapy
HGPRT: Hypoxanthine guanine phosphoribosyl transferase
TSM: Tumor stem media
GFP/LUC: Green fluorescent protein/Luciferase
IMPDH: Inosine monophosphate dehydrogenase
LC/MS: Liquid chromatography/Mass spectrometry
2D-Hpx: 2,8-deuterium hypoxanthine
MPA: Mycophenolic acid
ER: Enhancement ratio
BLI: Bioluminescent imaging
MMF: Mycophenolate mofetil
R5P: Ribose-5-phosphate
GBM: Glioblastoma
DNS: *De novo* synthesis
F16BP: Fructose 1,6-bisphosphate
AMP: Adenosine monophosphate
GMP: Guanosine monophosphate
BBB: Blood-brain barrier
NIH: National Institute of Health
NCI: National Cancer Institute
NINDS: National Institute of Neurological Disease and Stroke
