# Supplementary material for: Purine salvage promotes treatment resistance in H3K27M-mutant diffuse midline glioma
Source: Cancer Metab. 2024 Apr 9;12:11. doi: 10.1186/s40170-024-00341-7 (PMC11003124; doi:10.1186/s40170-024-00341-7)
Supplement: Supplementary file 1 — Additional file 1: Supplemental Figure 1. Patient-derived DMG-H3K27M isogenic cell lines and tumors represent the appropriate biology. A.) Quantification of N-acetylaspartate in normal brain vs DIPGXIII xenograft tumor tissue. Statistical analysis was performed using a two-tailed t-test. B.) Immunoblot analysis for H3K27M expression and corresponding H3K27me3 signal in patient-derived DMG-H3K27M isogenic models. C.) and D.) Normalized endpoint CellTiter-Glo 3D luminescence values representing the abundance of DIPGXIII (C.)and BT245 (D.) H3K27M-isogenic cells after 7 days of growth. E.) and F.) Radiation response CellTiter-Glo 3D viability assay curves for DIPGXIII (E.) and BT245 (F.) H3K27M-isogenice cell line pairs normalized to 0Gy control. [file 40170_2024_341_MOESM1_ESM.pdf]

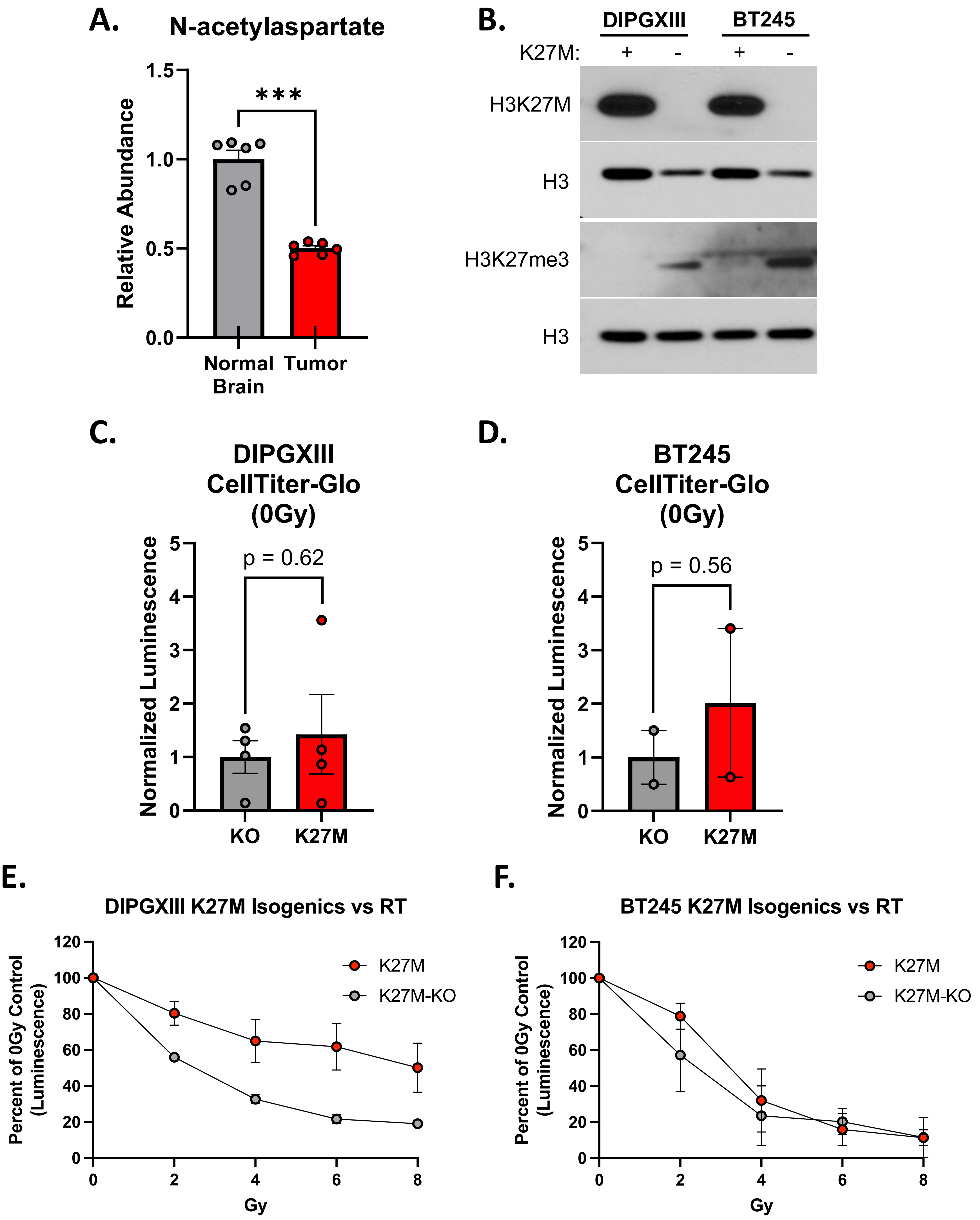
A.
N-acetylaspartate
Relative Abundance
1.5
1.0
0.5
0.0
***
Normal Brain
Tumor
B.
DIPGXIII
BT245
K27M:
+
-
+
-
H3K27M
H3
H3K27me3
H3
C.
DIPGXIII CellTiter-Glo (0Gy)
Normalized Luminescence
p = 0.62
KO
K27M
D.
BT245 CellTiter-Glo (0Gy)
Normalized Luminescence
p = 0.56
KO
K27M
E.
DIPGXIII K27M Isogenics vs RT
Percent of 0Gy Control (Luminescence)
K27M
K27M-KO
Gy
F.
BT245 K27M Isogenics vs RT
Percent of 0Gy Control (Luminescence)
K27M
K27M-KO
Gy
