# Supplementary figures and images for: Purine salvage promotes treatment resistance in H3K27M-mutant diffuse midline glioma
Source: Cancer Metab. 2024 Apr 9;12:11. doi: 10.1186/s40170-024-00341-7 (PMC11003124; doi:10.1186/s40170-024-00341-7)

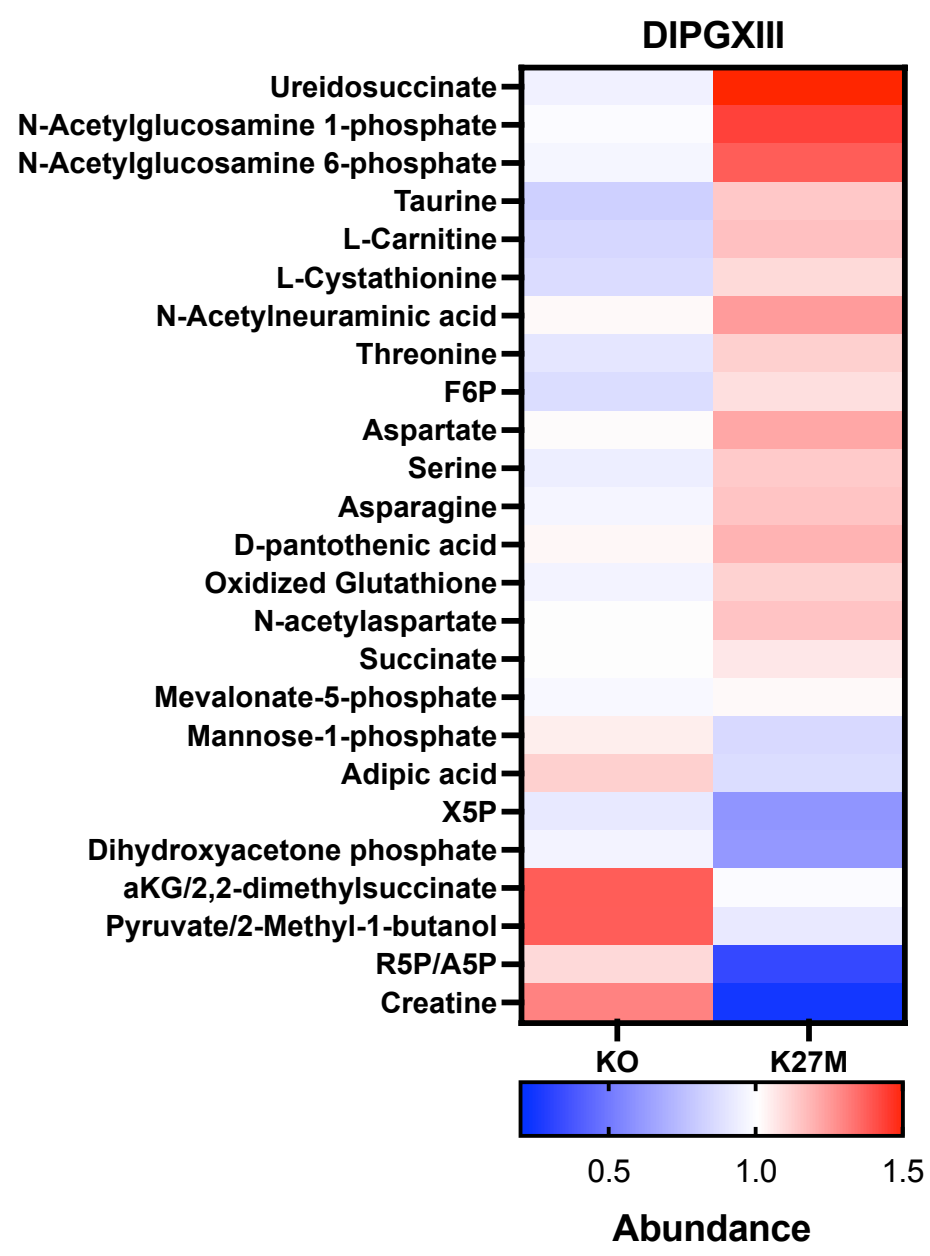

Supplement: Supplementary file 2 — Additional file 2: Supplemental Figure 2. The H3K27M mutation in DIPGXIII cells facilitates altered metabolism. Metabolite levels in untreated DIPGXIII H3K27M-isogenic cell lines as measured using LC/MS. Data represent the top 25 significantly different metabolites between H3K27M-KO and H3K27M cells. Data are ordered by difference in average median centered abundance in descending order. Metabolite extractions were performed in triplicate. [file 40170_2024_341_MOESM2_ESM.pdf]

**A.** DIPGXIII  
Glutamine

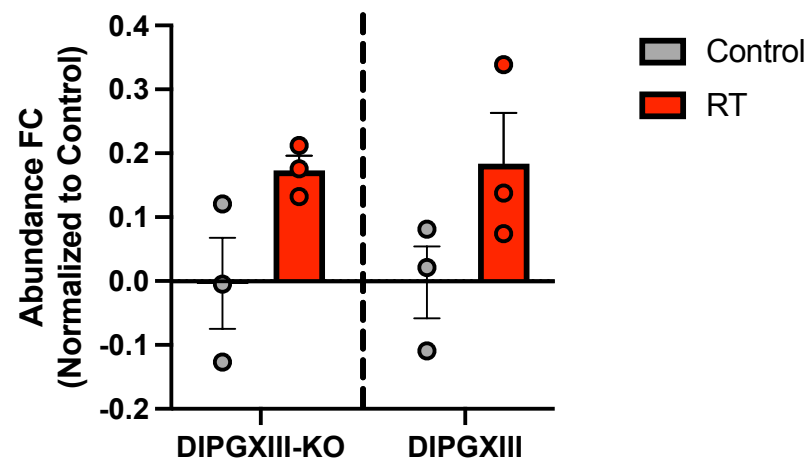

**B.** BT245  
Aspartate

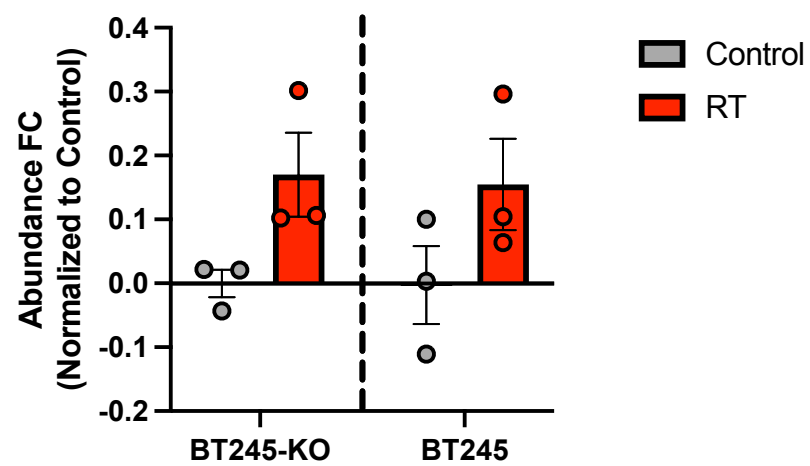

**C.** DIPGXIII  
Xanthine

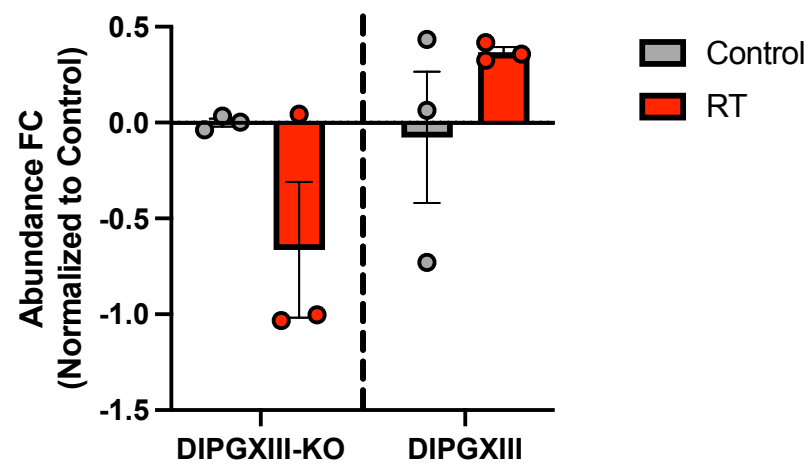

**D.** BT245  
dGDP/ADP

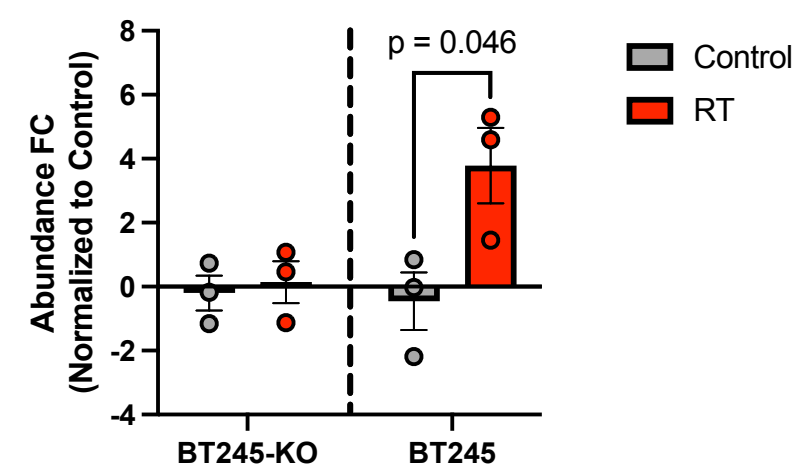

Supplement: Supplementary file 5 — Additional file 5: Supplemental Figure 3. Radiation-induced metabolite abundance changes vary. A.) and B.) Quantification of representative metabolites glutamine in DIPGXIII cells (A.) and aspartate in BT245 cells (B.) whose RT-induced abundance changes were similar despite the presence or absence of the H3K27M mutation. C.) and D.) Quantification of representative metabolites xanthine in DIPGXIII cells (C.) and dGDP/ADP in BT245 cells (D.) whose RT-induced abundance varied based on the expression of the H3K27M mutation. Statistical analyses were performed using a two-tailed t-test in GraphPad Prism 10.0. [file 40170_2024_341_MOESM5_ESM.pdf]

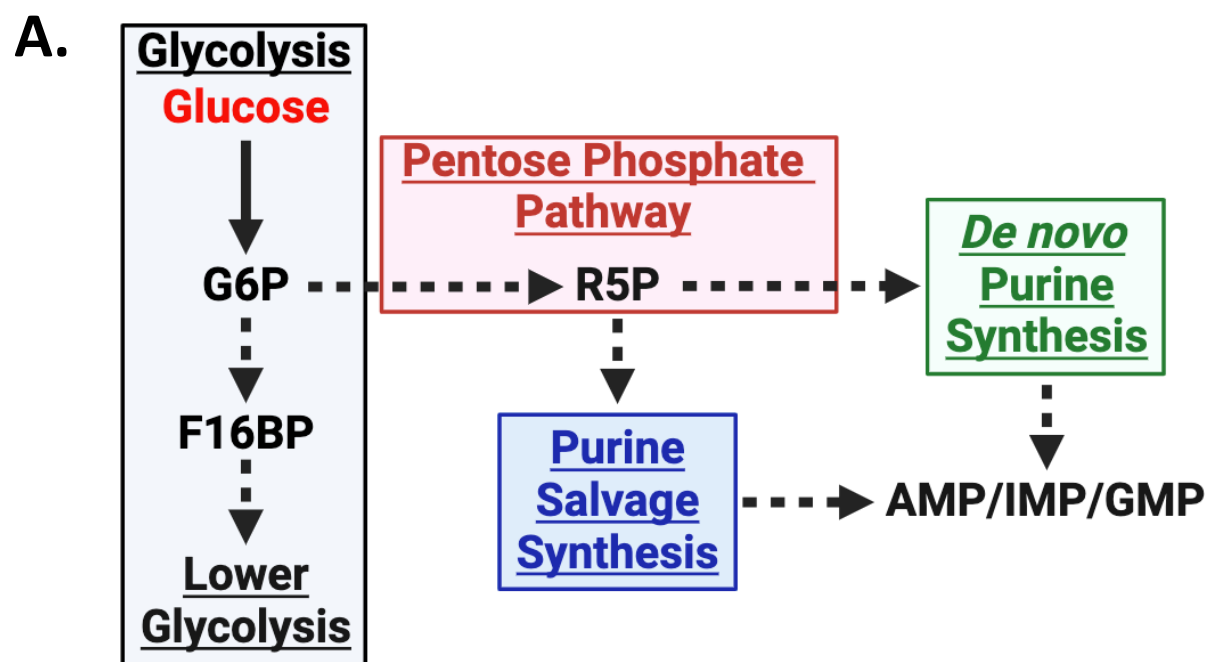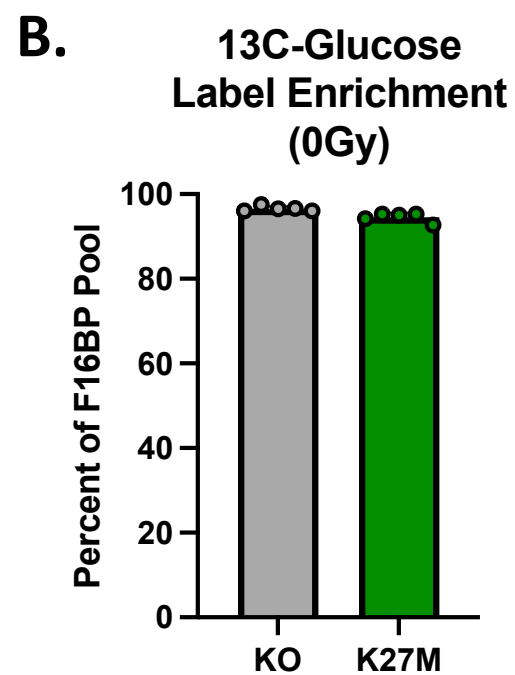

**C.**  $^{13}\text{C}$  F16BP Labeling

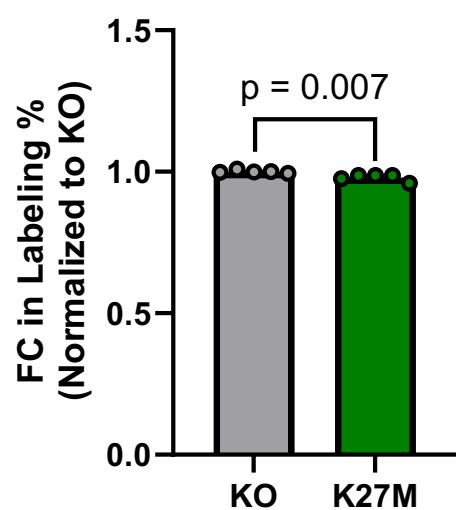

**D.**  $^{13}\text{C}$  R5P Labeling

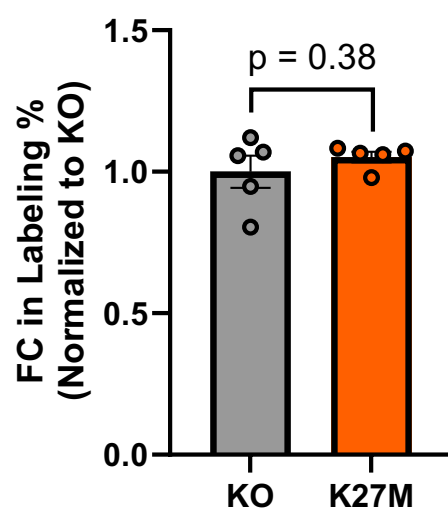

**E.**  $^{13}\text{C}$ -GMP Labeling

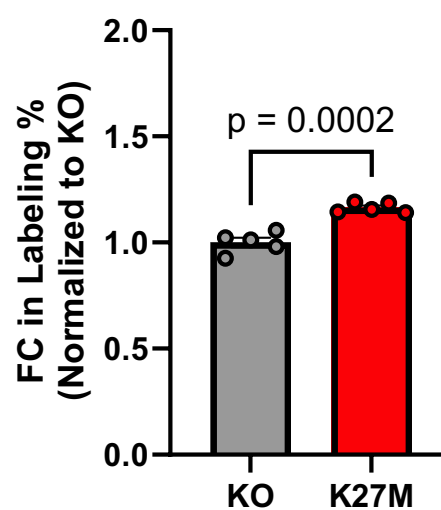

**F.**  $^{13}\text{C}$ -AMP Labeling

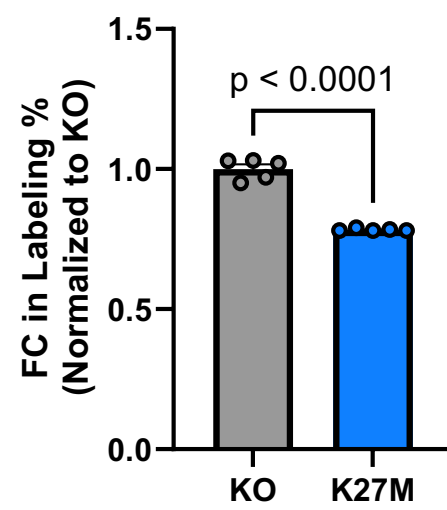

Supplement: Supplementary file 6 — Additional file 6: Supplemental Figure 4. Stable isotope tracing of U13C-Glucose uptake and usage in purine synthesis. A.) Schematic of glucose metabolism through glycolysis and the Pentose Phosphate Pathway shunt towards purine synthetic pathways. Schematic was created using BioRender.com. B.) Relative enrichment of U13C-Glucose-derived label in F16BP pools in unirradiated DIPGXIII H3K27M-isogenic cells. C.-G) Relative abundance of13C-labeled (C.) F16BP, (D.) R5P, (E.) IMP, (F.)AMP, and (G.) GMP in DIPGXIII H3K27M-isogenic cells. Data are normalized to H3K27M-KO samples. Statistical analyses were performed using two-tailed t-tests in GraphPad Prism 10.0. [file 40170_2024_341_MOESM6_ESM.pdf]

**A.**

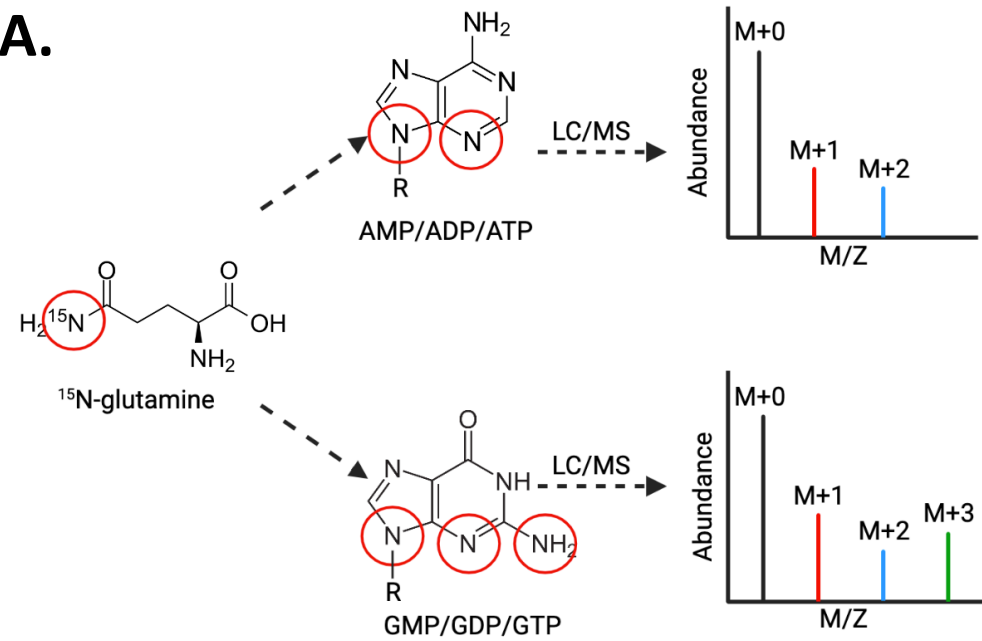

**B.**

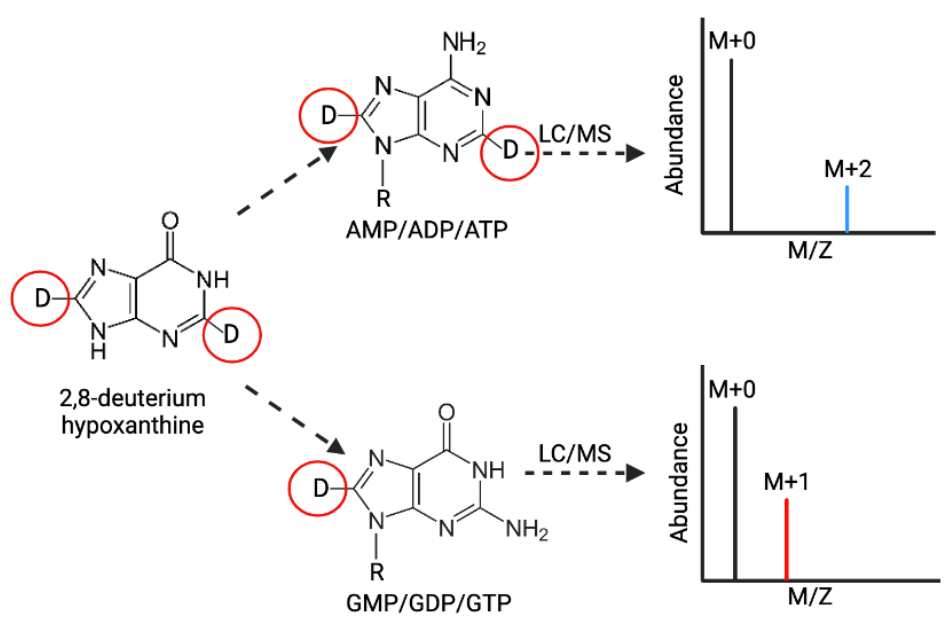

**C.**

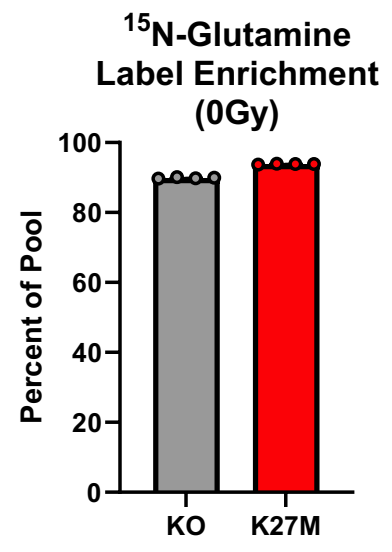

**D.**

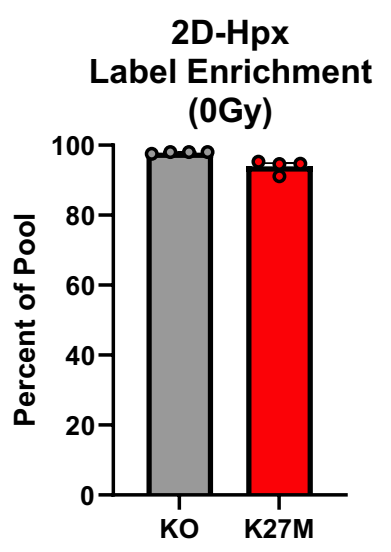

**E.**

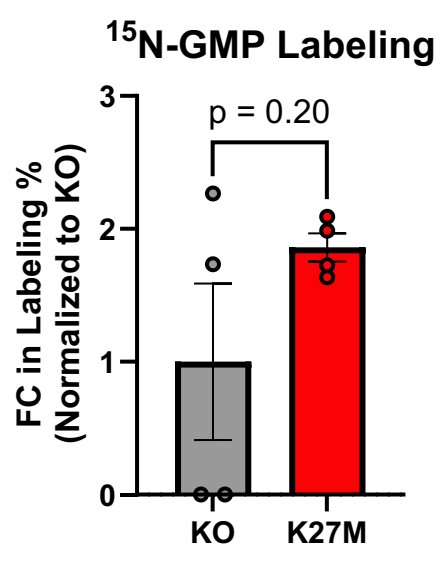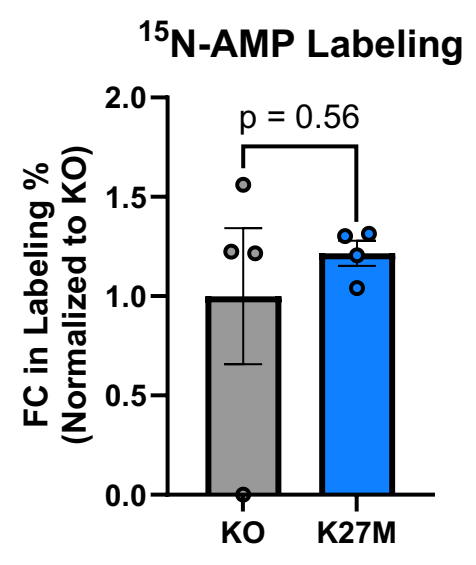

**F.**

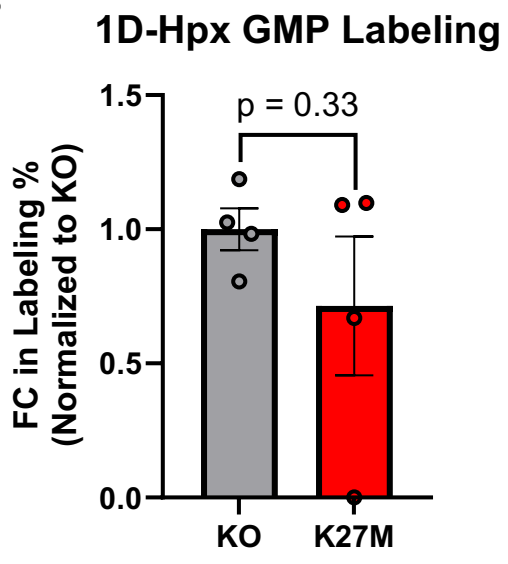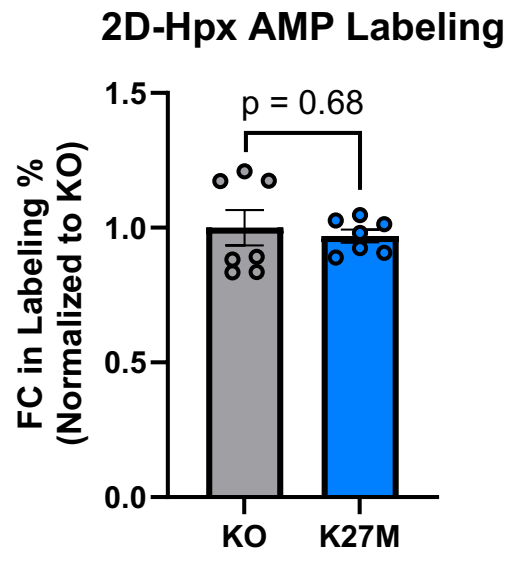

Supplement: Supplementary file 7 — Additional file 7: Supplemental Figure 5. Stable isotope tracing of de novo and salvage purine synthesis. A.) and B.) Schematics for (A.) 15N-Gln tracing and (B.) 2D-Hpx and the respective labeling pattern of each purine metabolite. Schematics were created using BioRender.com. C.) and D.) Percent of tracer metabolite enrichment in (C.)15N-Gln and (D.) 2D-Hpx pools in unirradiated DIPGXIII H3K27M-isogenic cells. E.) FC in relative 15N-labeled GMP(left) and AMP (right) abundance between irradiated H3K27M-isogenic cell lines. Data are normalized to H3K27M-KO samples. F.) FC in relative deuterium-labeled GMP (left) andAMP (right) abundance between irradiated H3K27M-isogenic cell lines. Data are normalized to H3K27M-KO samples. Statistical analyses were performed using two-tailed t-tests in GraphPad Prism 10.0. [file 40170_2024_341_MOESM7_ESM.pdf]

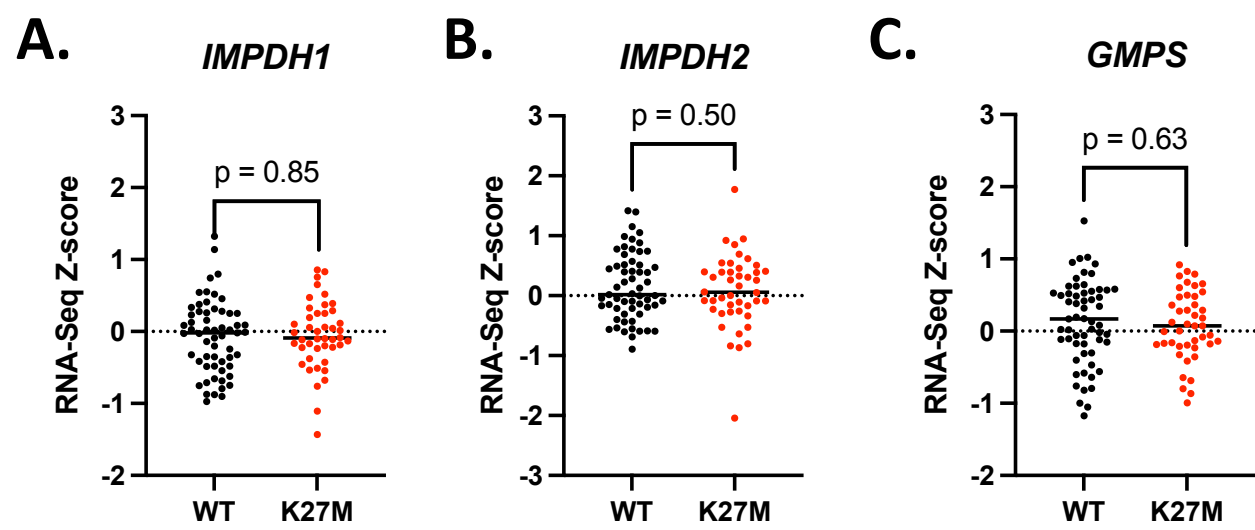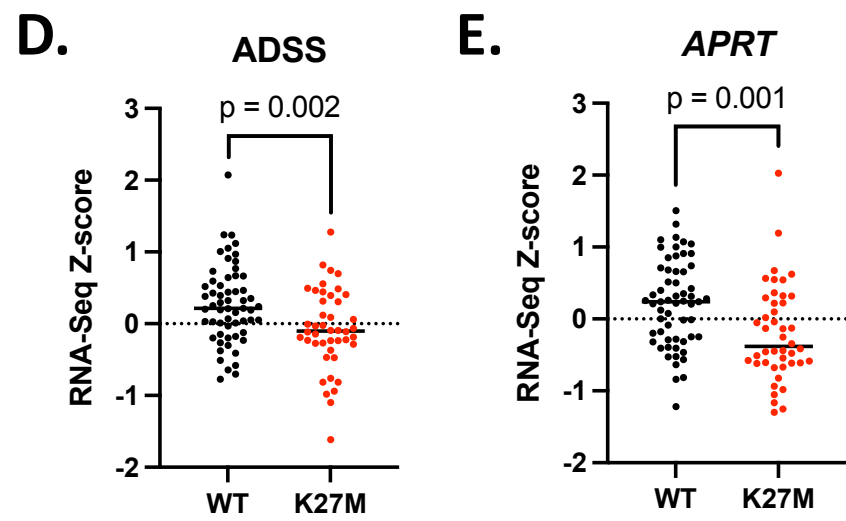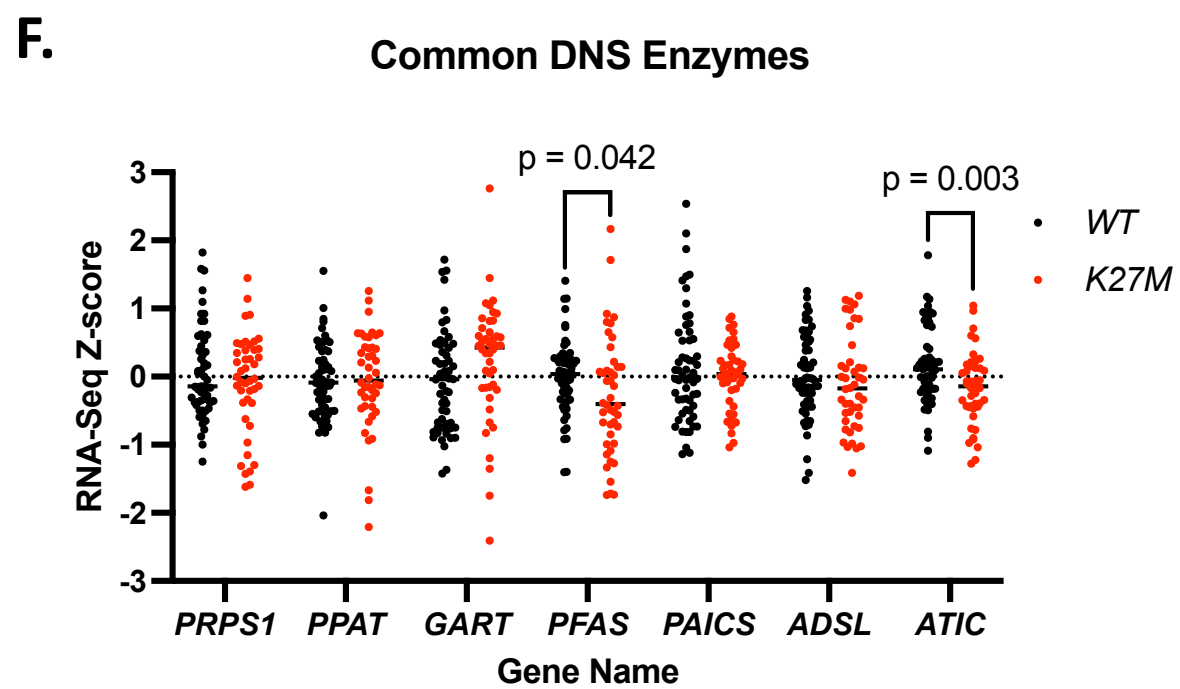

Supplement: Supplementary file 8 — Additional file 8: Supplemental Figure 6. Purine metabolic enzyme expression. A-E.) Publicly available RNAseq Z-score data for (A.) IMPDH1, (B.) IMPDH2, (C.) GMPS, (D.) ADSS, and (E.) APRT transcript expression from pediatric high-grade gliomas (pHGG) was obtained through PedCBioPortal and filtered to include only samples with known H3 mutational status (for both H3F3A and HIST1H3B) to include all known H3WT (n=59) and combined H3K27M (H3F3A-mut+HIST1H3B-mut) samples (n=44). F.) RNAseq Z-score data for all common de novo purine synthesis enzymes in H3WT or H3K27M-mutant pHGG tumors. Statistical analyses were performed using two-tailed t-tests in GraphPad Prism 10.0. [file 40170_2024_341_MOESM8_ESM.pdf]

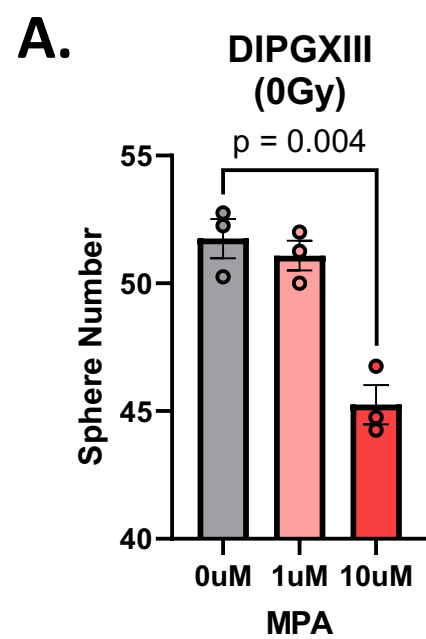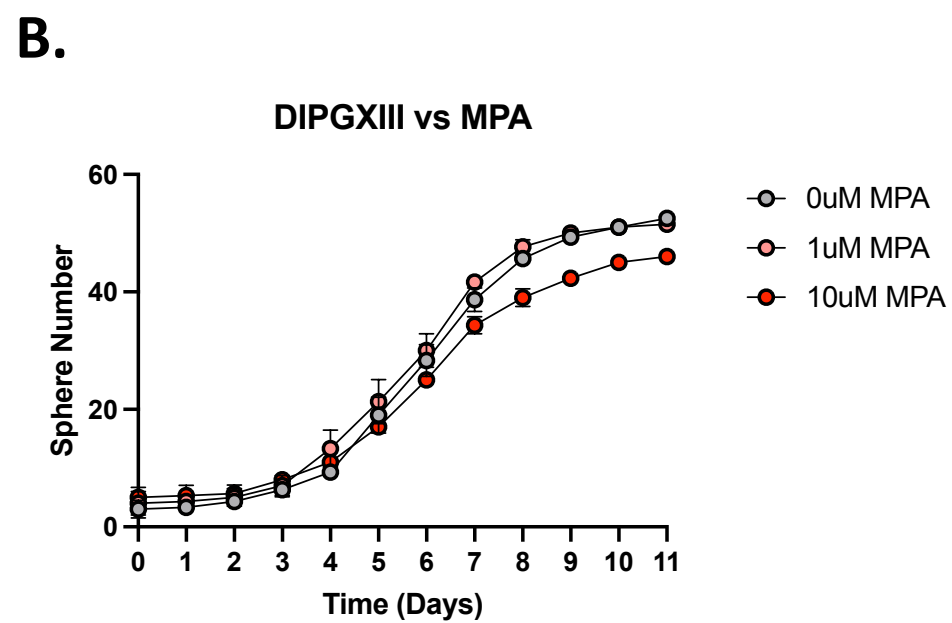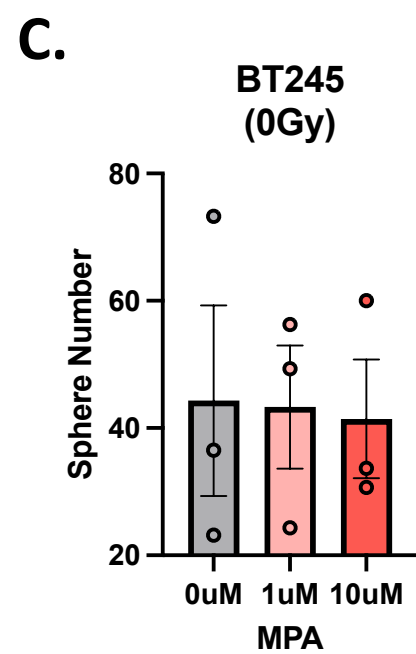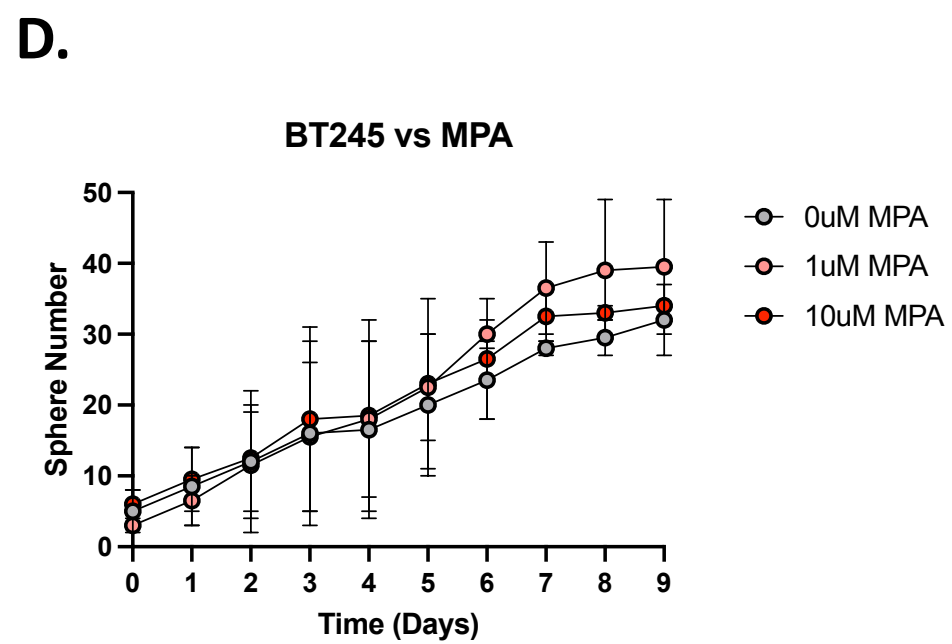

Supplement: Supplementary file 9 — Additional file 9: Supplemental Figure 7. Single-agent mycophenolic acid has mixed efficacy in vitro. A.) and C.) Endpoint neurosphere counts of DIPGXIII (A.) and BT245 (C.) cells treated with increasing concentrations of MPA (0-10mM) during the long-term neurosphere growth assays. B.) and D.) Long-term growth curves over time of DIPXIII (B.) and BT245 (D.) cells treated with increasing concentrations of MPA (0-10mM). Endpoint and growth curve neurosphere data was acquired using a Cytation 5 plate reader and attached BioSpa incubator (Agilent Technologies). [file 40170_2024_341_MOESM9_ESM.pdf]

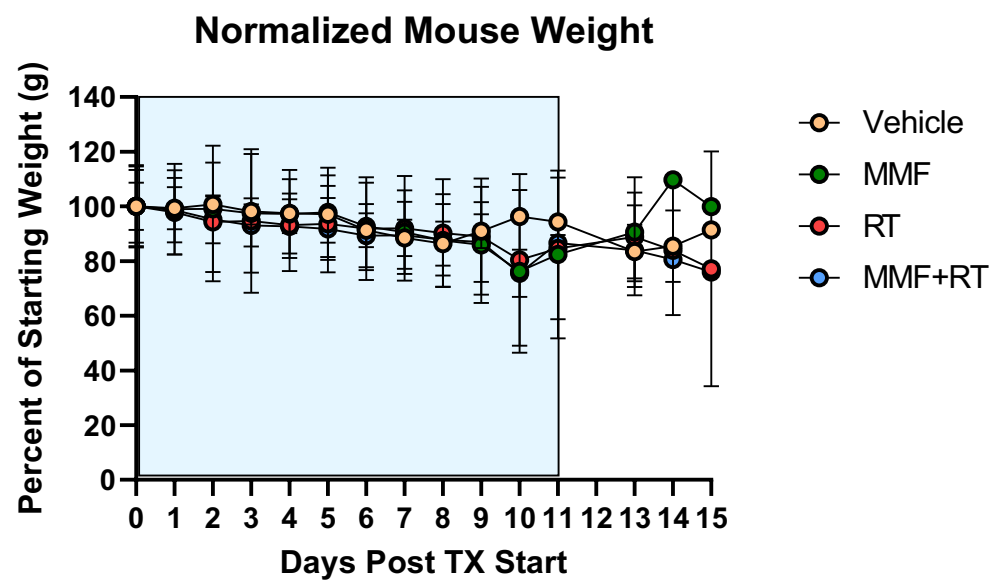

Supplement: Supplementary file 10 — Additional file 10: Supplemental Figure 8. DIPGXIII-LUC/GFP tumor-bearing Rag1-KO mouse weights are minimally affected after treatment course. Normalized measurement of mouse weight over treatment time course for DIPGXIII-GFP/LUC xenograft tumor-bearing mice. Blue box indicates treatment period. [file 40170_2024_341_MOESM10_ESM.pdf]

**A.**  $^{13}\text{C}_8$ -Guanine  
Label Enrichment

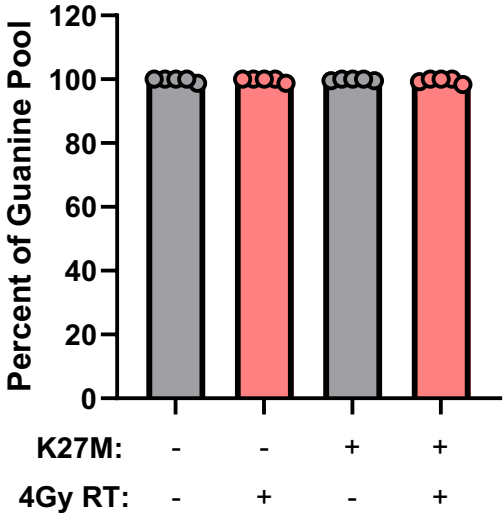

**B.**  $^{13}\text{C}_8$ -GMP Labeling

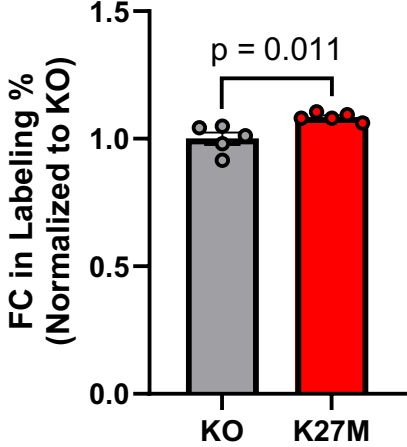

**C.**  $^{13}\text{C}_8$ -GMP Labeling  
(4Gy)

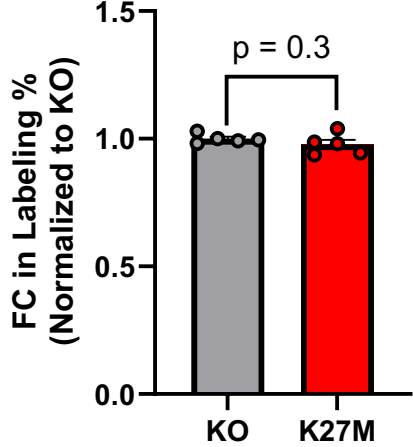

Supplement: Supplementary file 13 — Additional file 13: Supplemental Figure 11.13C8-guanine tracing of purine salvage before and after RT. A.) Percent enrichment of 13C8-guanine tracer in DIPGXIII H3K27M-isogenic cell lines before and 3hrs after 4Gy RT. B.)FC in relative abundance of 13C8-labeled GMP in unirradiated DIPGXIII H3K27M-isogenic cell lines. Data are normalized to H3K27M-KO samples. C.) FC in relative abundance of 13C8--labeled GMP between DIPGXIII H3K27M-isogenic cell lines 3hrs after 4Gy single dose RT. Data are normalized to H3K27M-KO samples. Statistical analyses were performed using two-tailed t-tests in GraphPad Prism 10.0. [file 40170_2024_341_MOESM13_ESM.pdf]

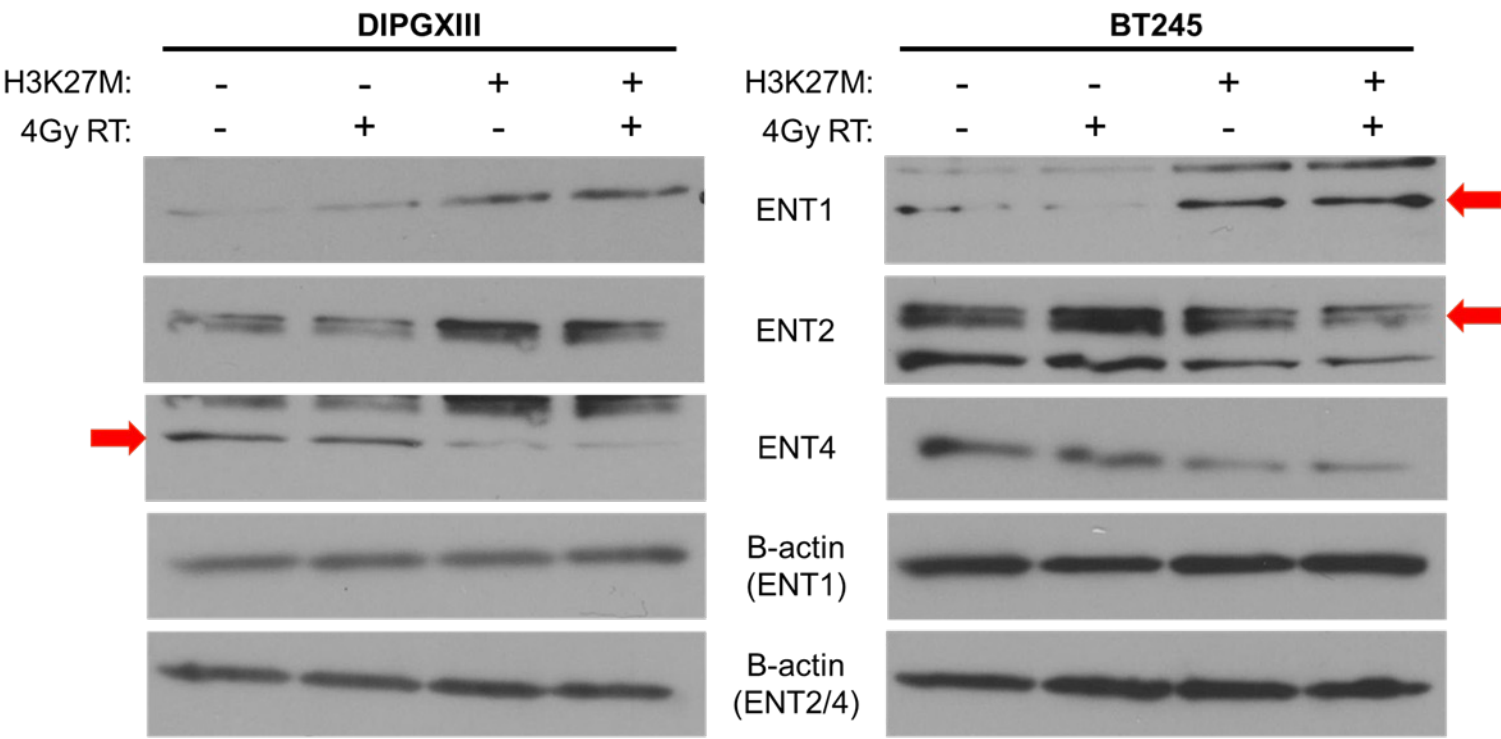

Supplement: Supplementary file 14 — Additional file 14: Supplemental Figure 12. RT does not induce changes in ENT expression. Immunoblot analysis of ENTs 1, 2, and 4 in DIPGXIII and BT245 H3K27M-isogenic cell lines before and 3hr after 4Gy RT. [file 40170_2024_341_MOESM14_ESM.pdf]

**A.**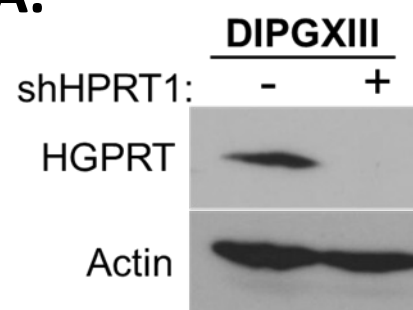**B.**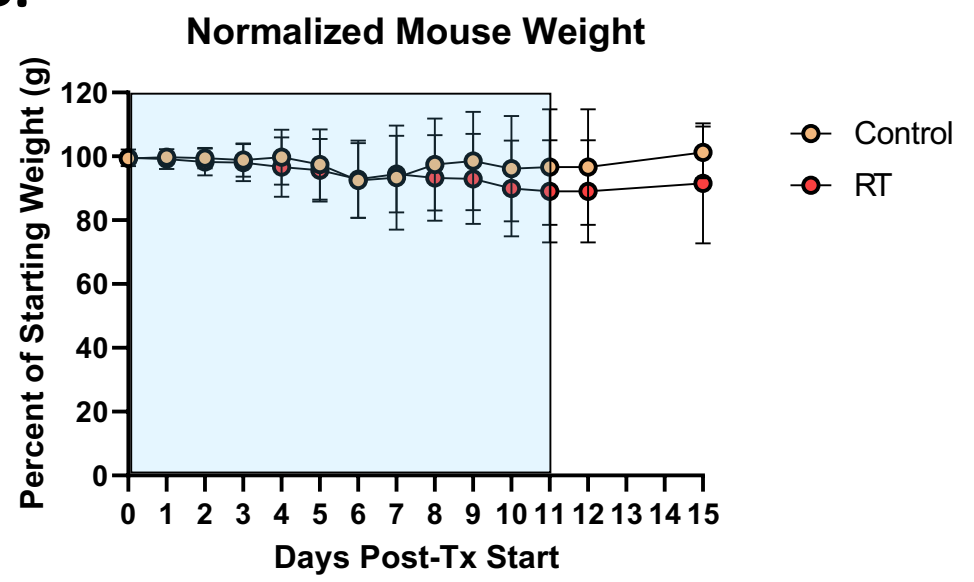

Supplement: Supplementary file 15 — Additional file 15: Supplemental Figure 13. DIPGXIII-LUC/GFP-shHPRT1 tumor-bearing Rag1-KO mouse weights are minimally affected after RT. A.) Immunoblot of control DIPGXIII and DIPGXIII-shHPRT1 cells probed for HGPRT expression. B.) Normalized measurement of mouse weight over treatment time course for DIPGXIII-GFP/LUC-shHPRT1 xenograft tumor-bearing mice. Blue box indicates treatmentperiod. [file 40170_2024_341_MOESM15_ESM.pdf]

A. *IMPDH1* in H3K27M Tumors

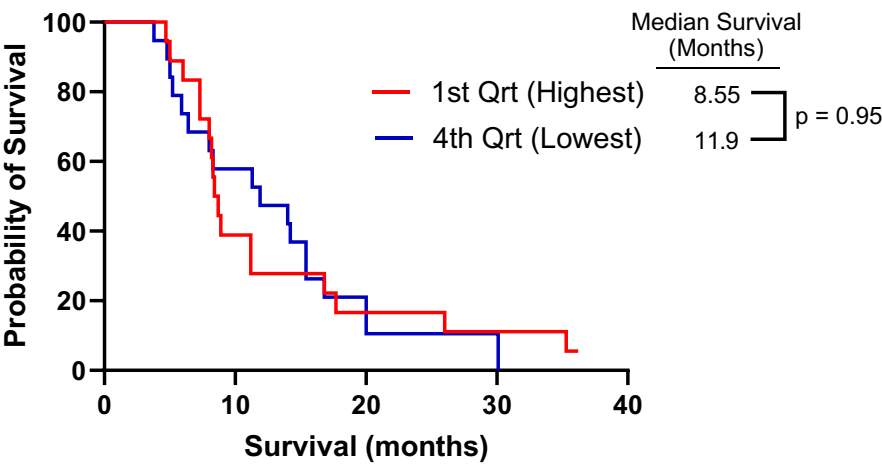

B. *IMPDH2* in H3K27M Tumors

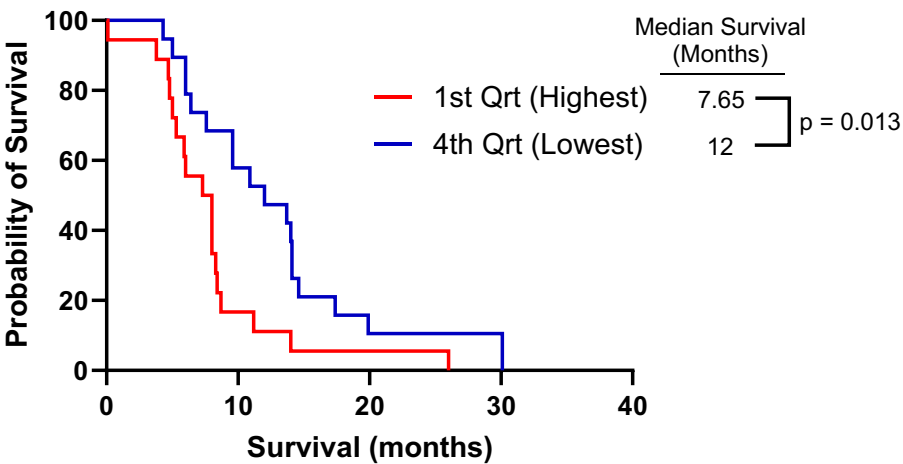

Supplement: Supplementary file 16 — Additional file 16: Supplemental Figure 14.IMPDH1/2 expression in H3K27M tumors. A.) and B.) Kaplan-Meier survival analysis of patient H3K27M-expressing tumors based on IMPDH1 (A.) and IMPDH2 (B.) expression where the 1st quartile represents the highest expression, and the 4th quartile represents the lowest expression. [file 40170_2024_341_MOESM16_ESM.pdf]
