# Supplementary material for: Purine salvage promotes treatment resistance in H3K27M-mutant diffuse midline glioma
Source: Cancer Metab. 2024 Apr 9;12:11. doi: 10.1186/s40170-024-00341-7 (PMC11003124; doi:10.1186/s40170-024-00341-7)
Supplement: Supplementary file 3 — Additional file 3: Supplemental Table 1. Global median centered abundances for the top 25 metabolites at baseline between H3K27M-isogenic cell line pairs. Metabolite name and average GMCA in H3K27M-KO and H3K27M DIPGXIII and BT245 cell line pairs and difference in abundance between H3K27M and H3K27M-KO. [file 40170_2024_341_MOESM3_ESM.pdf]

Supplemental Table 1: Baseline median-centered abundance fold changes for the top 25 metabolites between H3K27M-isogenic cells.

DIPGXIII Isogenics Baseline			
Metabolite	KO	K27M	Difference (K27M-KO)
Ureidosuccinate	0.943	1.533	0.590
N-Acetylglucosamine 1-phosphate	0.981	1.423	0.442
N-Acetylglucosamine 6-phosphate	0.962	1.368	0.406
Taurine	0.809	1.133	0.324
L-Carnitine	0.839	1.151	0.312
L-Cystathionine	0.857	1.092	0.235
N-Acetylneuraminic acid	1.016	1.238	0.222
Threonine	0.897	1.116	0.219
F6P	0.860	1.077	0.217
Aspartate	1.008	1.210	0.202
Serine	0.930	1.127	0.197
Asparagine	0.956	1.142	0.186
D-pantothenic acid	1.019	1.182	0.163
Oxidized Glutathione	0.952	1.110	0.157
N-acetylaspartate	1.001	1.144	0.143
Succinate	1.001	1.058	0.056
Mevalonate-5-phosphate	0.969	1.015	0.046
Mannose-1-phosphate	1.043	0.847	-0.196
Adipic acid	1.117	0.864	-0.252
X5P	0.908	0.594	-0.314
Dihydroxyacetone phosphate	0.953	0.604	-0.350
aKG/2,2-dimethylsuccinate	1.369	0.984	-0.385
Pyruvate/2-Methyl-1-butanol	1.366	0.915	-0.451
R5P/A5P	1.094	0.321	-0.773
Creatine	1.289	0.243	-1.046
BT245 Isogenics Baseline			
Metabolite	KO	K27M	Difference (K27M-KO)
Guanosine	0.512	2.364	1.851
Citramalic acid	0.418	1.034	0.616
Deoxycytidine	0.750	1.137	0.387
Histidine	0.996	1.300	0.304
Glutamine	0.909	1.133	0.224
4-Methyl-2-oxovaleric acid	0.860	1.039	0.179
Isopentyl acetate	0.892	1.044	0.152
Itaconic acid	0.927	1.068	0.141
Aspartate	1.008	0.883	-0.125
D-pantothenic acid	1.019	0.846	-0.173
Threonine	1.115	0.927	-0.188
Tyrosine	1.027	0.822	-0.205
Phenylalanine	1.009	0.787	-0.222
Methionine	1.007	0.765	-0.242
Reduced Glutathione	1.071	0.823	-0.248
Isoleucine	1.030	0.779	-0.251
N-Acetylglutamic acid	1.080	0.806	-0.274
N-Acetylglucosamine 6-phosphate	1.127	0.733	-0.394
Chorismic acid	1.045	0.645	-0.400
Adipic acid	1.152	0.698	-0.453
Creatine	1.363	0.850	-0.513
TDP	1.302	0.756	-0.545
Ureidosuccinate	1.381	0.810	-0.571
N-acetylaspartate	1.270	0.645	-0.625
N-acetylaspartylglutamate	1.325	0.626	-0.699
