# Supplementary material for: Purine salvage promotes treatment resistance in H3K27M-mutant diffuse midline glioma
Source: Cancer Metab. 2024 Apr 9;12:11. doi: 10.1186/s40170-024-00341-7 (PMC11003124; doi:10.1186/s40170-024-00341-7)
Supplement: Supplementary file 4 — Additional file 4: Supplemental Table 2. Post-RT FC for the top 25 metabolites between H3K27M-isogenic cell line pairs. Metabolite name and average post-RT FC in H3K27M-KO and H3K27M DIPGXIII and BT245 cell line pairs and difference in abundance between H3K27M and H3K27M-KO values. Purine metabolites denoted by an asterisk (*). [file 40170_2024_341_MOESM4_ESM.pdf]

Supplemental Table 2: Post-RT abundance fold change for the top 25 metabolites between H3K27M-isogenic cell line pairs.

DIPGXIII Isogenics post-RT			
Metabolite	KO	K27M	Difference (K27M-KO)
Xanthine*	-0.664	0.367	1.031
Pyridoxine	-0.925	0.047	0.972
R5P/A5P*	0.856	1.736	0.879
X5P*	0.446	0.985	0.539
AMP*	-0.302	0.211	0.513
aKG/ 2,2-dimethylsuccinate	-0.441	0.006	0.448
UMP	-0.089	0.309	0.398
Hypoxanthine	0.378	0.768	0.390
G1P	0.109	0.402	0.293
Tyrosine	-0.525	-0.243	0.281
Allantoin	-0.165	0.109	0.275
Proline	-0.278	-0.007	0.271
dGDP/ADP*	-0.304	-0.067	0.237
β-NAD	-0.368	-0.199	0.169
Oxidized glutathione	0.015	-0.167	-0.182
Phosphoenolpyruvate	1.728	1.540	-0.188
Lactate	0.314	0.101	-0.213
Ureidosuccinate	-0.271	-0.558	-0.286
Pyruvate/2-Methyl-1-butanol	0.183	-0.133	-0.317
2-Phosphoglycerate	2.384	2.046	-0.338
Sedoheptulose-7-phosphate	0.150	-0.217	-0.366
Dihydroxyacetone phosphate	1.012	0.629	-0.383
G6P	0.212	-0.202	-0.414
Mannose-1-phosphate	0.722	0.269	-0.452
3-Hydroxyphenylacetic acid	2.895	2.379	-0.516
BT245 Isogenics post-RT			
Metabolite	KO	K27M	Difference (K27M-KO)
dGDP/ADP*	0.139	3.784	3.644
Guanine*	-0.762	0.040	0.802
Chorismic acid	0.137	0.784	0.647
R5P/A5P*	-0.866	-0.330	0.536
Creatine	-0.349	0.156	0.504
Hypoxanthine*	-0.180	0.186	0.366
Proline	-0.169	0.119	0.288
Lactate	0.148	-0.173	-0.321
F6P	0.266	-0.056	-0.322
Allantoin	0.055	-0.275	-0.330
Asparagine	-0.064	-0.408	-0.343
Oxidized Glutathione	0.280	-0.073	-0.353
Isopentyl acetate	0.216	-0.147	-0.364
Malonic acid	0.272	-0.094	-0.365
Histidine	-0.127	-0.535	-0.408
N-Acetylglucosamine 1-phosphate	0.248	-0.195	-0.443
Deoxycytidine	0.501	0.051	-0.450
Itaconic acid	0.186	-0.276	-0.462
4-Methyl-2-oxovaleric acid	0.269	-0.293	-0.562
Arginine	-0.026	-0.606	-0.581
L-Carnitine	0.005	-0.747	-0.752
L-Cystathionine	0.167	-0.906	-1.073
Guanosine*	0.112	-1.034	-1.147
NADH	-1.479	-2.783	-1.304
Citramalic acid	1.451	0.001	-1.450
