# Supplementary material for: Purine salvage promotes treatment resistance in H3K27M-mutant diffuse midline glioma
Source: Cancer Metab. 2024 Apr 9;12:11. doi: 10.1186/s40170-024-00341-7 (PMC11003124; doi:10.1186/s40170-024-00341-7)
Supplement: Supplementary file 11 — Additional file 11: Supplemental Figure 9. U13C-Glucose tracing of glucose uptake and PPP activity following RT. A.) and C.) FC in 13C label enrichment in (A.) F16BP and (C.) R5Pin H3K27M-isogenic cell lines 3hrs after 4Gy single dose RT. RT conditions for each cell line are normalized to the respective 0Gy control. B.) and D.) FC of unlabeled and 13C-labeled ion abundances following RT for (B.)F16BP and (D.) R5P in H3K27M-isogenic cell lines following irradiation with a single 4Gy dose of RT. Statistical analyses were performed using two-tailed t-tests in GraphPad Prism 10.0. [file 40170_2024_341_MOESM11_ESM.pdf]

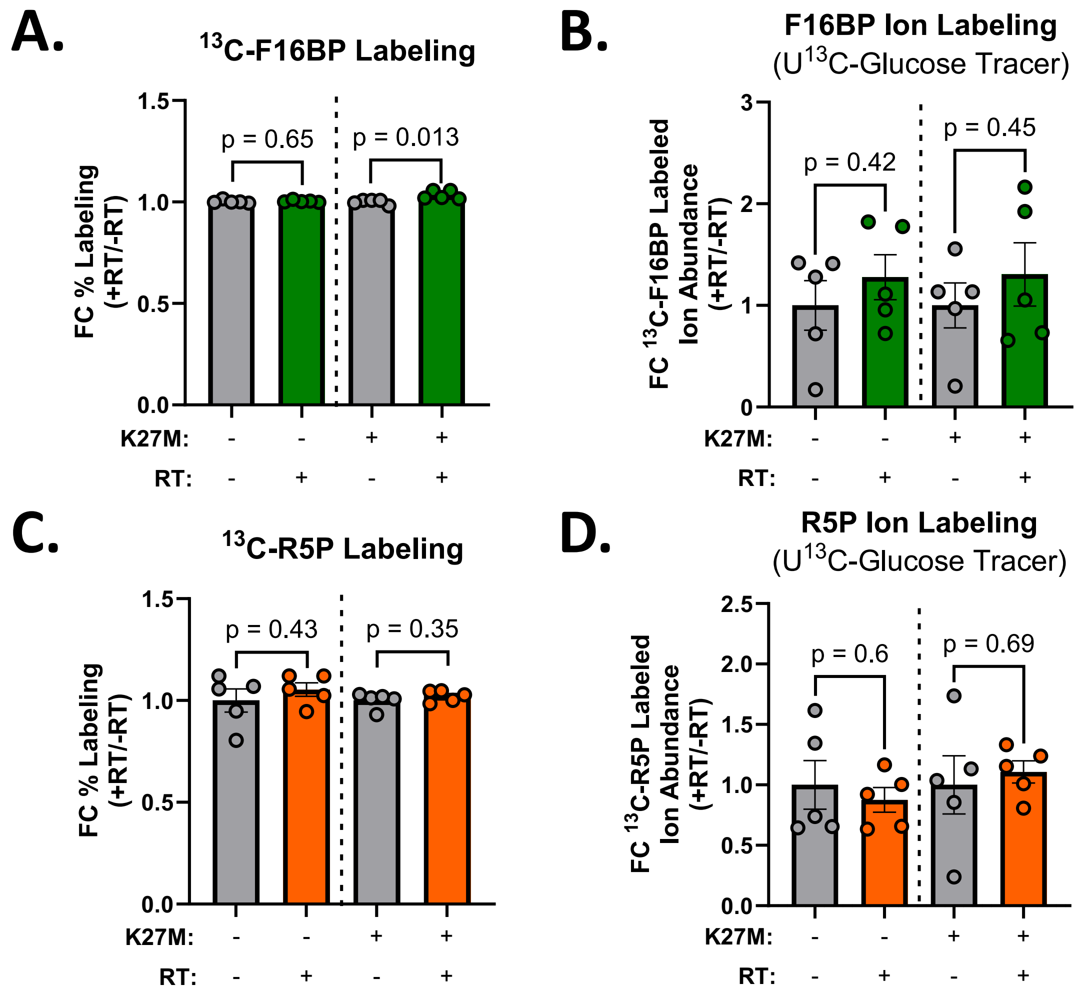

A.
13C-F16BP Labeling
FC % Labeling (+RT/-RT)
p = 0.65
p = 0.013
K27M:
RT:
B.
F16BP Ion Labeling
(U13C-Glucose Tracer)
FC 13C-F16BP Labeled Ion Abundance (+RT/-RT)
p = 0.42
p = 0.45
C.
13C-R5P Labeling
p = 0.43
p = 0.35
D.
R5P Ion Labeling
(U13C-Glucose Tracer)
FC 13C-R5P Labeled Ion Abundance (+RT/-RT)
p = 0.6
p = 0.69
