# Supplementary material for: Purine salvage promotes treatment resistance in H3K27M-mutant diffuse midline glioma
Source: Cancer Metab. 2024 Apr 9;12:11. doi: 10.1186/s40170-024-00341-7 (PMC11003124; doi:10.1186/s40170-024-00341-7)
Supplement: Supplementary file 12 — Additional file 12: Supplemental Figure 10.15N-Gln and 2D-Hpx Isotope tracing of purine synthesis following RT. A.) Percent of tracer metabolite enrichment of 15N-Gln in H3K27M-isogenic cells 3hrs after 4Gy RT. B.) FC in 15N label enrichment in GMP in H3K27M-isogenic cell lines 3hrs after 4Gy single dose RT. RT conditions for each cell line are normalized to the respective 0Gy control. C.) FC in relative 15N-labeled GMP abundance between irradiated H3K27M-isogenic cell lines. Data are normalized to H3K27M-KO samples. D.) FC in 15N-labeled AMP ion abundance 3hrs after 4Gy single dose RT in H3K27M-isogenic cell lines. RT-treated samples were normalized to their respective unirradiated controls. E.) FC in the relative abundance of 15N-labeled AMP in DIPGXIII H3K27M-isogenic cell lines before (gray bars) and 3hrs after 4Gy RT (blue bars). Data are normalized to untreated control samples. F.) FC in relative 15N-labeled AMP abundance between irradiated H3K27M-isogenic cell lines.Data are normalized to H3K27M-KO samples. G.)Percent of tracer metabolite enrichment of 2D-Hpx in H3K27M-isogenic cells 3hrs after 4Gy RT. H.) FC in deuterium label enrichment in GMP in H3K27M-isogenic cell lines 3hrs after 4Gy single dose RT. RT conditions for each cell line are normalized to the respective 0Gy control. I.) FC in relative deuterium-labeled GMP abundance between irradiated H3K27M-isogenic cell lines. Data are normalized to H3K27M-KO samples. J.) FC in 2D-labeled AMP ion abundance 3hrs after 4Gy single dose RT in H3K27M-isogenic cell lines. RT-treated samples were normalized to their respective unirradiated controls. K.) FC in the relative abundance of 2D-labeled AMP in DIPGXIII H3K27M-isogenic cell lines before (gray bars) and 3hrs after 4Gy RT (blue bars). Data are normalized to untreated control samples. L.) FC in relative 2D-labeled AMP abundance between irradiated H3K27M-isogenic cell lines. Data are normalized to H3K27M-KO samples. Statistical analyses were performed using tw [file 40170_2024_341_MOESM12_ESM.pdf]

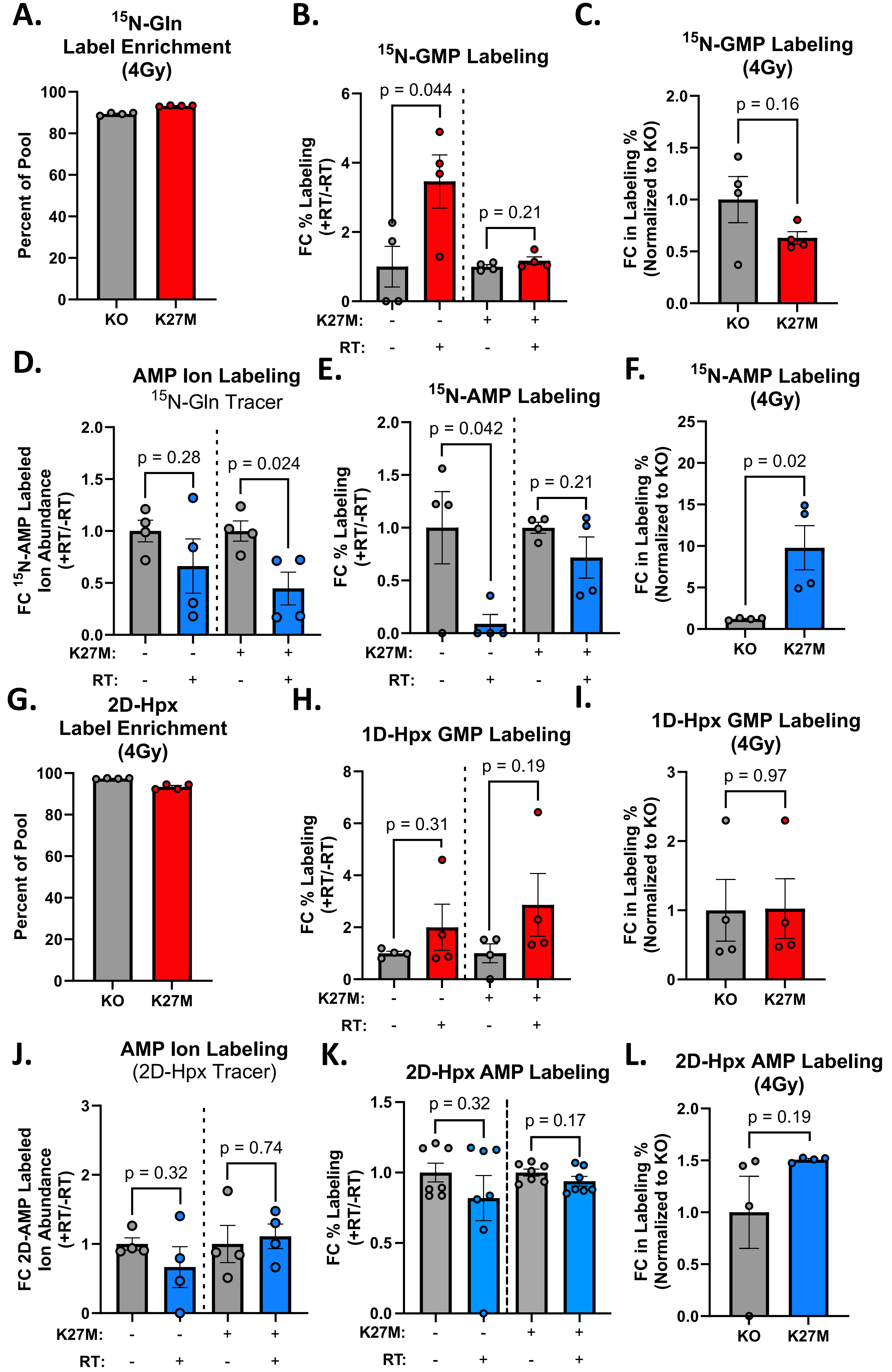

A.
15N-Gln Label Enrichment (4Gy)
Percent of Pool
KO
K27M
B.
15N-GMP Labeling
FC % Labeling (+RT/-RT)
p = 0.044
p = 0.21
K27M:
RT:
C.
15N-GMP Labeling (4Gy)
FC in Labeling % (Normalized to KO)
p = 0.16
D.
AMP Ion Labeling
15N-Gln Tracer
FC 15N-AMP Labeled Ion Abundance (+RT/-RT)
p = 0.28
p = 0.024
E.
15N-AMP Labeling
p = 0.042
p = 0.21
F.
15N-AMP Labeling (4Gy)
p = 0.02
G.
2D-Hpx Label Enrichment (4Gy)
H.
1D-Hpx GMP Labeling
p = 0.31
p = 0.19
I.
1D-Hpx GMP Labeling (4Gy)
p = 0.97
J.
AMP Ion Labeling
(2D-Hpx Tracer)
FC 2D-AMP Labeled Ion Abundance (+RT/-RT)
p = 0.32
p = 0.74
K.
2D-Hpx AMP Labeling
p = 0.32
p = 0.17
L.
2D-Hpx AMP Labeling (4Gy)
p = 0.19
