# Supplementary material for: Purine salvage promotes treatment resistance in H3K27M-mutant diffuse midline glioma
Source: Cancer Metab. 2024 Apr 9;12:11. doi: 10.1186/s40170-024-00341-7 (PMC11003124; doi:10.1186/s40170-024-00341-7)
Supplement: Supplementary file 17 — Additional file 17. [file 40170_2024_341_MOESM17_ESM.pdf]

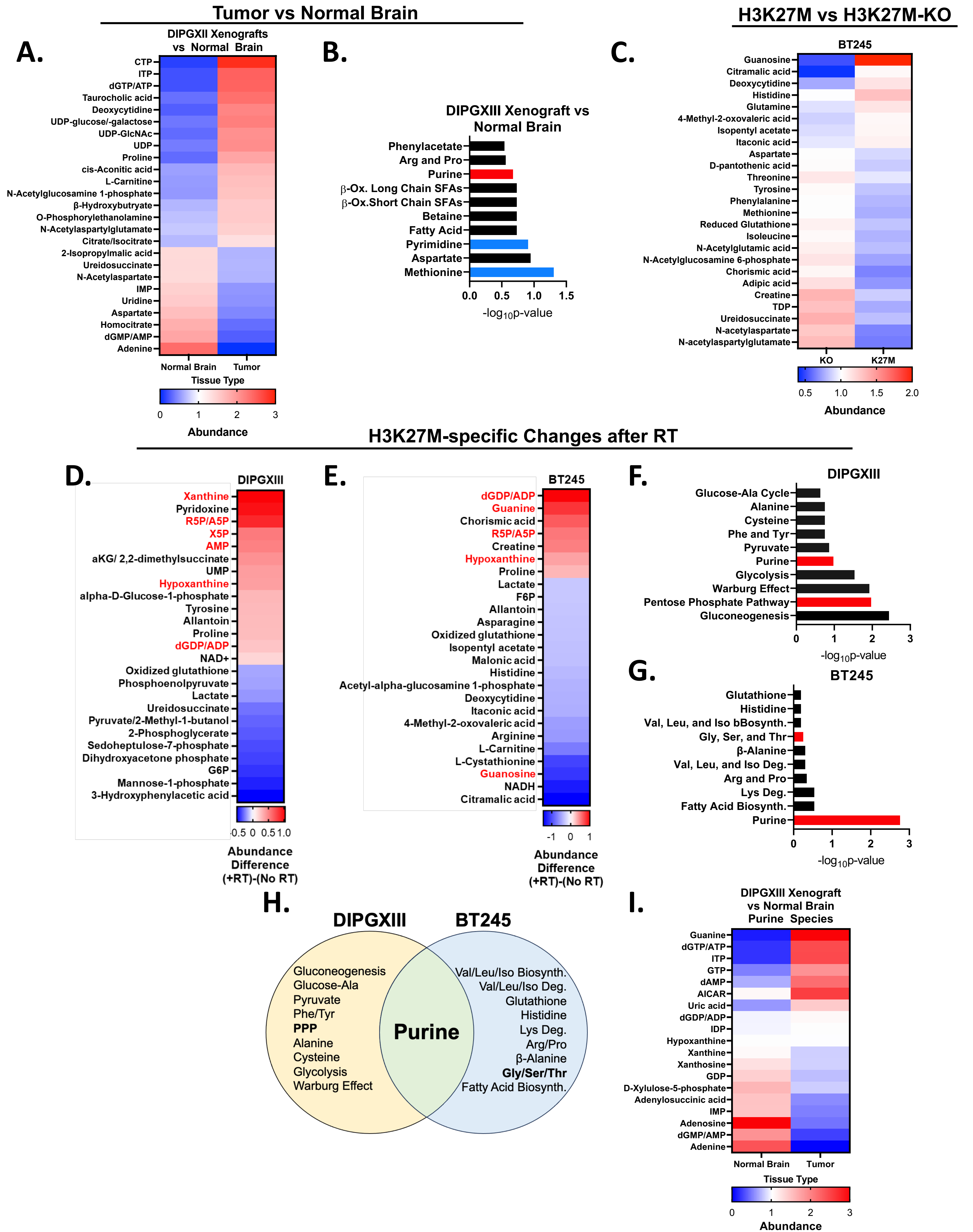

Tumor vs Normal Brain
A.
DIPGXII Xenografts vs Normal Brain
CTP
ITP
dGTP/ATP
Taurocholic acid
Deoxycytidine
UDP-glucose/-galactose
UDP-GlcNAc
UDP
Proline
cis-Aconitic acid
L-Carnitine
N-Acetylglucosamine 1-phosphate
β-Hydroxybutryate
O-Phosphorylethanolamine
N-Acetylaspartylglutamate
Citrate/Isocitrate
2-Isopropylmalic acid
Ureidosuccinate
N-Acetylaspartate
IMP
Uridine
Aspartate
Homocitrate
dGMP/AMP
Adenine
Normal Brain
Tumor
Tissue Type
0
1
2
3
Abundance
B.
DIPGXIII Xenograft vs Normal Brain
Phenylacetate
Arg and Pro
Purine
β-Ox. Long Chain SFAs
β-Ox.Short Chain SFAs
Betaine
Fatty Acid
Pyrimidine
Aspartate
Methionine
0.0
0.5
1.0
1.5
-log10p-value
H3K27M vs H3K27M-KO
C.
BT245
Guanosine
Citramalic acid
Deoxycytidine
Histidine
Glutamine
4-Methyl-2-oxovaleric acid
Isopentyl acetate
Itaconic acid
Aspartate
D-pantothenic acid
Threonine
Tyrosine
Phenylalanine
Methionine
Reduced Glutathione
Isoleucine
N-Acetylglutamic acid
N-Acetylglucosamine 6-phosphate
Chorismic acid
Adipic acid
Creatine
TDP
Ureidosuccinate
N-acetylaspartate
N-acetylaspartylglutamate
KO
K27M
0.5
1.0
1.5
2.0
Abundance
H3K27M-specific Changes after RT
D.
DIPGXIII
Xanthine
Pyridoxine
R5P/A5P
X5P
AMP
aKG/ 2,2-dimethylsuccinate
UMP
Hypoxanthine
alpha-D-Glucose-1-phosphate
Tyrosine
Allantoin
Proline
dGDP/ADP
NAD+
Oxidized glutathione
Phosphoenolpyruvate
Lactate
Ureidosuccinate
Pyruvate/2-Methyl-1-butanol
2-Phosphoglycerate
Sedoheptulose-7-phosphate
Dihydroxyacetone phosphate
G6P
Mannose-1-phosphate
3-Hydroxyphenylacetic acid
-0.5 0 0.5 1.0
Abundance Difference (+RT)-(No RT)
E.
BT245
dGDP/ADP
Guanine
Chorismic acid
R5P/A5P
Creatine
Hypoxanthine
Proline
Lactate
F6P
Allantoin
Asparagine
Oxidized glutathione
Isopentyl acetate
Malonic acid
Histidine
Acetyl-alpha-glucosamine 1-phosphate
Deoxycytidine
Itaconic acid
4-Methyl-2-oxovaleric acid
Arginine
L-Carnitine
L-Cystathionine
Guanosine
NADH
Citramalic acid
-1
0
1
Abundance Difference (+RT)-(No RT)
F.
DIPGXIII
Glucose-Ala Cycle
Alanine
Cysteine
Phe and Tyr
Pyruvate
Purine
Glycolysis
Warburg Effect
Pentose Phosphate Pathway
Gluconeogenesis
0
1
2
3
-log10p-value
G.
BT245
Glutathione
Histidine
Val, Leu, and Iso bBosynth.
Gly, Ser, and Thr
β-Alanine
Val, Leu, and Iso Deg.
Arg and Pro
Lys Deg.
Fatty Acid Biosynth.
Purine
0
1
2
3
-log10p-value
H.
DIPGXIII
BT245
Gluconeogenesis
Glucose-Ala
Pyruvate
Phe/Tyr
PPP
Alanine
Cysteine
Glycolysis
Warburg Effect
Purine
Val/Leu/Iso Biosynth.
Val/Leu/Iso Deg.
Glutathione
Histidine
Lys Deg.
Arg/Pro
β-Alanine
Gly/Ser/Thr
Fatty Acid Biosynth.
I.
DIPGXIII Xenograft vs Normal Brain Purine Species
Guanine
dGTP/ATP
ITP
GTP
dAMP
AICAR
Uric acid
dGDP/ADP
IDP
Hypoxanthine
Xanthine
Xanthosine
GDP
D-Xylulose-5-phosphate
Adenylosuccinic acid
IMP
Adenosine
dGMP/AMP
Adenine
Normal Brain
Tumor
Tissue Type
0
1
2
3
Abundance

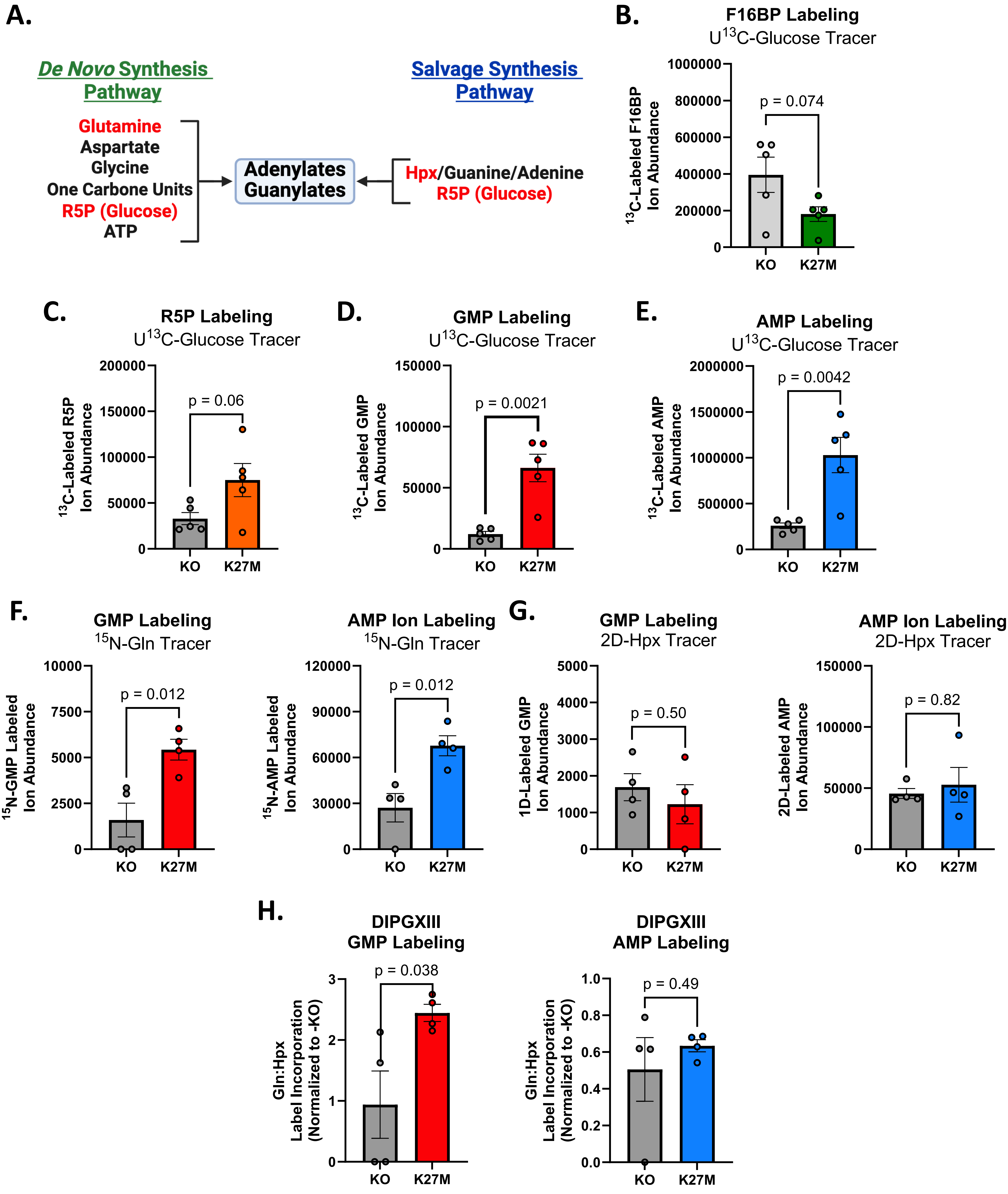

A.
De Novo Synthesis Pathway
Glutamine
Aspartate
Glycine
One Carbone Units
R5P (Glucose)
ATP
Adenylates
Guanylates
Salvage Synthesis Pathway
Hpx/Guanine/Adenine
R5P (Glucose)
B.
F16BP Labeling
U13C-Glucose Tracer
13C-Labeled F16BP Ion Abundance
p = 0.074
KO
K27M
C.
R5P Labeling
U13C-Glucose Tracer
13C-Labeled R5P Ion Abundance
p = 0.06
D.
GMP Labeling
U13C-Glucose Tracer
13C-Labeled GMP Ion Abundance
p = 0.0021
E.
AMP Labeling
U13C-Glucose Tracer
13C-Labeled AMP Ion Abundance
p = 0.0042
F.
GMP Labeling
15N-Gln Tracer
15N-GMP Labeled Ion Abundance
p = 0.012
AMP Ion Labeling
15N-Gln Tracer
15N-AMP Labeled Ion Abundance
p = 0.012
G.
GMP Labeling
2D-Hpx Tracer
1D-Labeled GMP Ion Abundance
p = 0.50
AMP Ion Labeling
2D-Hpx Tracer
2D-Labeled AMP Ion Abundance
p = 0.82
H.
DIPGXIII
GMP Labeling
Gln:Hpx Label Incorporation (Normalized to -KO)
p = 0.038
DIPGXIII
AMP Labeling
p = 0.49

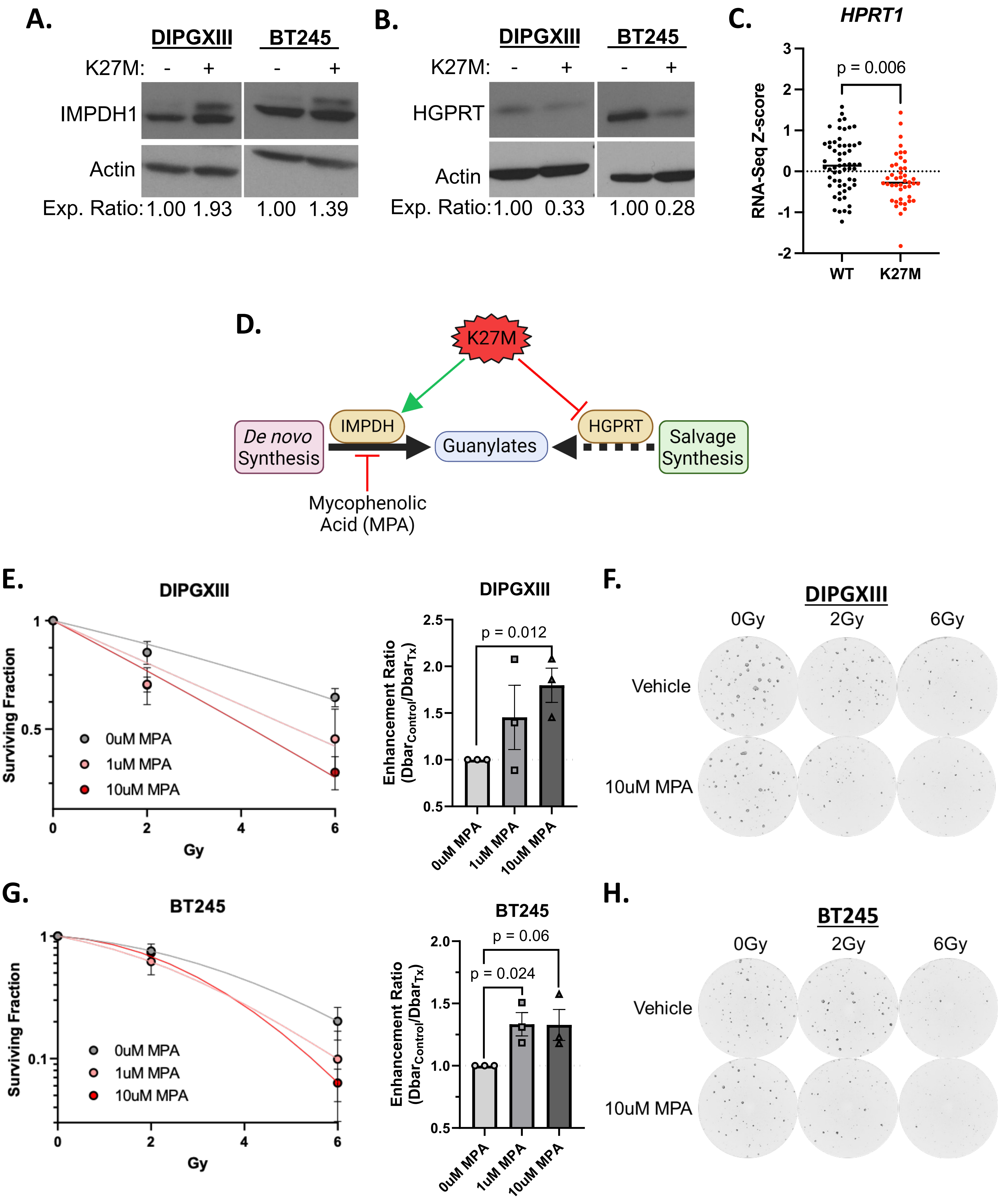

A.
DIPGXIII
BT245
K27M: - + - +
IMPDH1
Actin
Exp. Ratio: 1.00 1.93 1.00 1.39
B.
DIPGXIII
BT245
K27M: - + - +
HGPRT
Actin
Exp. Ratio: 1.00 0.33 1.00 0.28
C.
HPRT1
p = 0.006
RNA-Seq Z-score
WT
K27M
D.
K27M
IMPDH
HGPRT
De novo Synthesis
Guanylates
Salvage Synthesis
Mycophenolic Acid (MPA)
E.
DIPGXIII
Surviving Fraction
0uM MPA
1uM MPA
10uM MPA
Gy
DIPGXIII
p = 0.012
Enhancement Ratio (DbarControl/DbarTx)
0uM MPA
1uM MPA
10uM MPA
F.
DIPGXIII
0Gy
2Gy
6Gy
Vehicle
10uM MPA
G.
BT245
Surviving Fraction
0uM MPA
1uM MPA
10uM MPA
Gy
BT245
p = 0.06
p = 0.024
Enhancement Ratio (DbarControl/DbarTx)
0uM MPA
1uM MPA
10uM MPA
H.
BT245
0Gy
2Gy
6Gy
Vehicle
10uM MPA

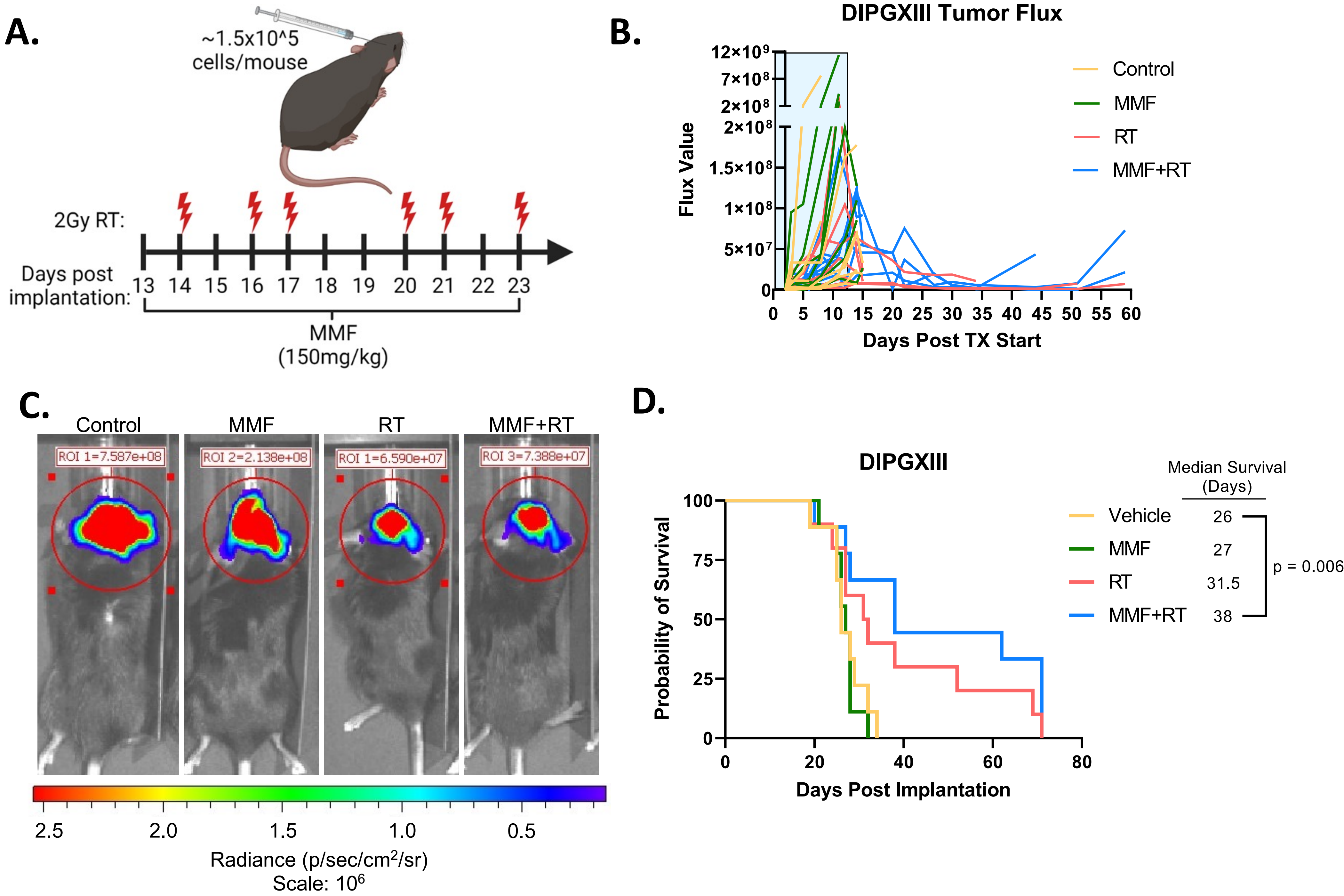

A.
~1.5x10^5 cells/mouse
2Gy RT:
Days post implantation:
13 14 15 16 17 18 19 20 21 22 23
MMF (150mg/kg)
B.
DIPGXIII Tumor Flux
Flux Value
Days Post TX Start
Control
MMF
RT
MMF+RT
C.
Control
MMF
RT
MMF+RT
ROI 1=7.587e+08
ROI 2=2.138e+08
ROI 1=6.590e+07
ROI 3=7.388e+07
2.5 2.0 1.5 1.0 0.5
Radiance (p/sec/cm²/sr)
Scale: 10⁶
D.
DIPGXIII
Probability of Survival
Days Post Implantation
Median Survival (Days)
Vehicle 26
MMF 27
RT 31.5
MMF+RT 38
p = 0.006

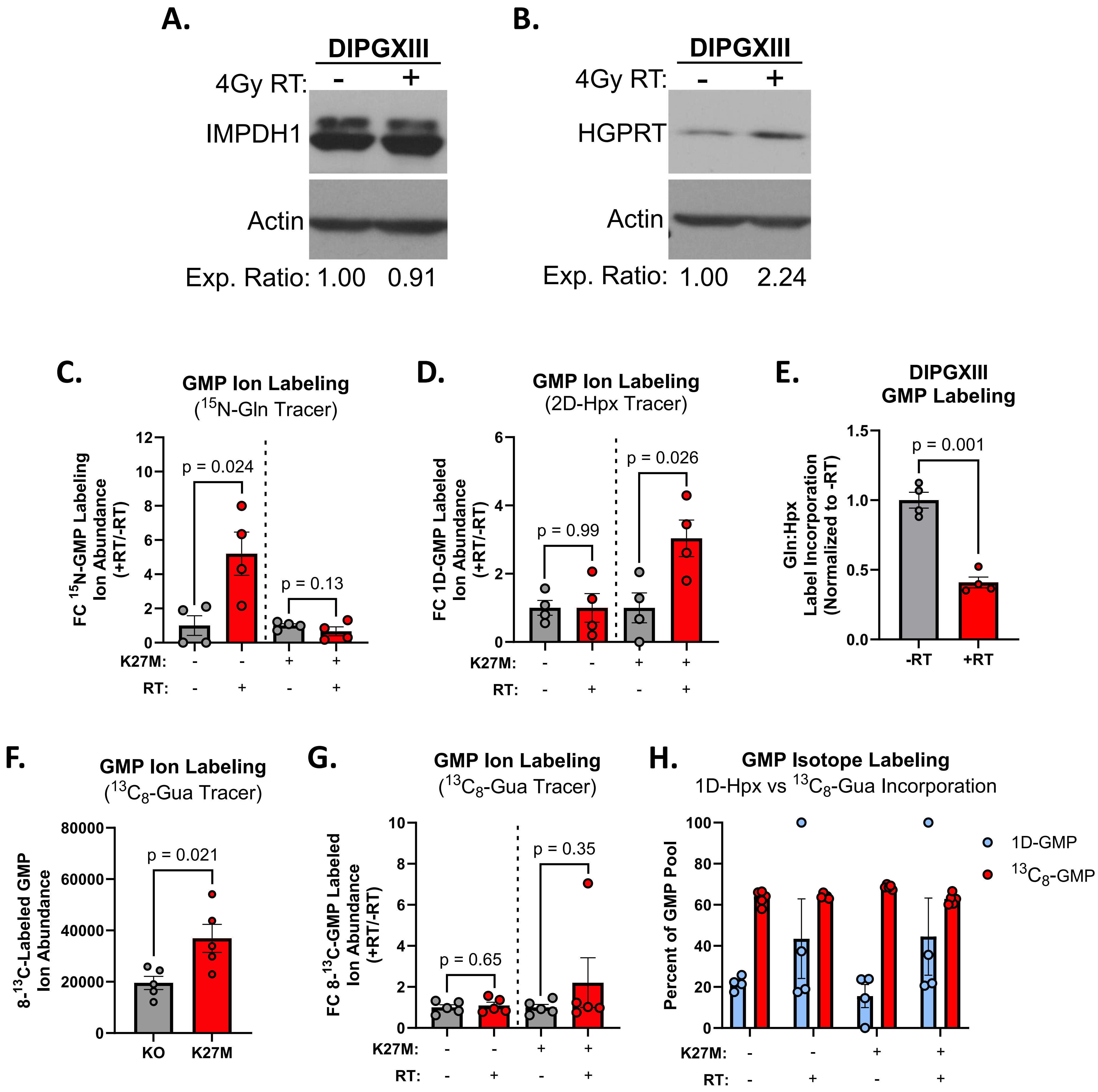

A.
DIPGXIII
4Gy RT: - +
IMPDH1
Actin
Exp. Ratio: 1.00 0.91
B.
DIPGXIII
4Gy RT: - +
HGPRT
Actin
Exp. Ratio: 1.00 2.24
C.
GMP Ion Labeling
(15N-Gln Tracer)
FC 15N-GMP Labeling Ion Abundance (+RT/-RT)
p = 0.024
p = 0.13
K27M: - - + +
RT: - + - +
D.
GMP Ion Labeling
(2D-Hpx Tracer)
FC 1D-GMP Labeled Ion Abundance (+RT/-RT)
p = 0.99
p = 0.026
K27M: - - + +
RT: - + - +
E.
DIPGXIII
GMP Labeling
Gln:Hpx Label Incorporation (Normalized to -RT)
p = 0.001
-RT +RT
F.
GMP Ion Labeling
(13C8-Gua Tracer)
8-13C-Labeled GMP Ion Abundance
p = 0.021
KO K27M
G.
GMP Ion Labeling
(13C8-Gua Tracer)
FC 8-13C-GMP Labeled Ion Abundance (+RT/-RT)
p = 0.65
p = 0.35
K27M: - - + +
RT: - + - +
H.
GMP Isotope Labeling
1D-Hpx vs 13C8-Gua Incorporation
Percent of GMP Pool
1D-GMP
13C8-GMP
K27M: - - + +
RT: - + - +

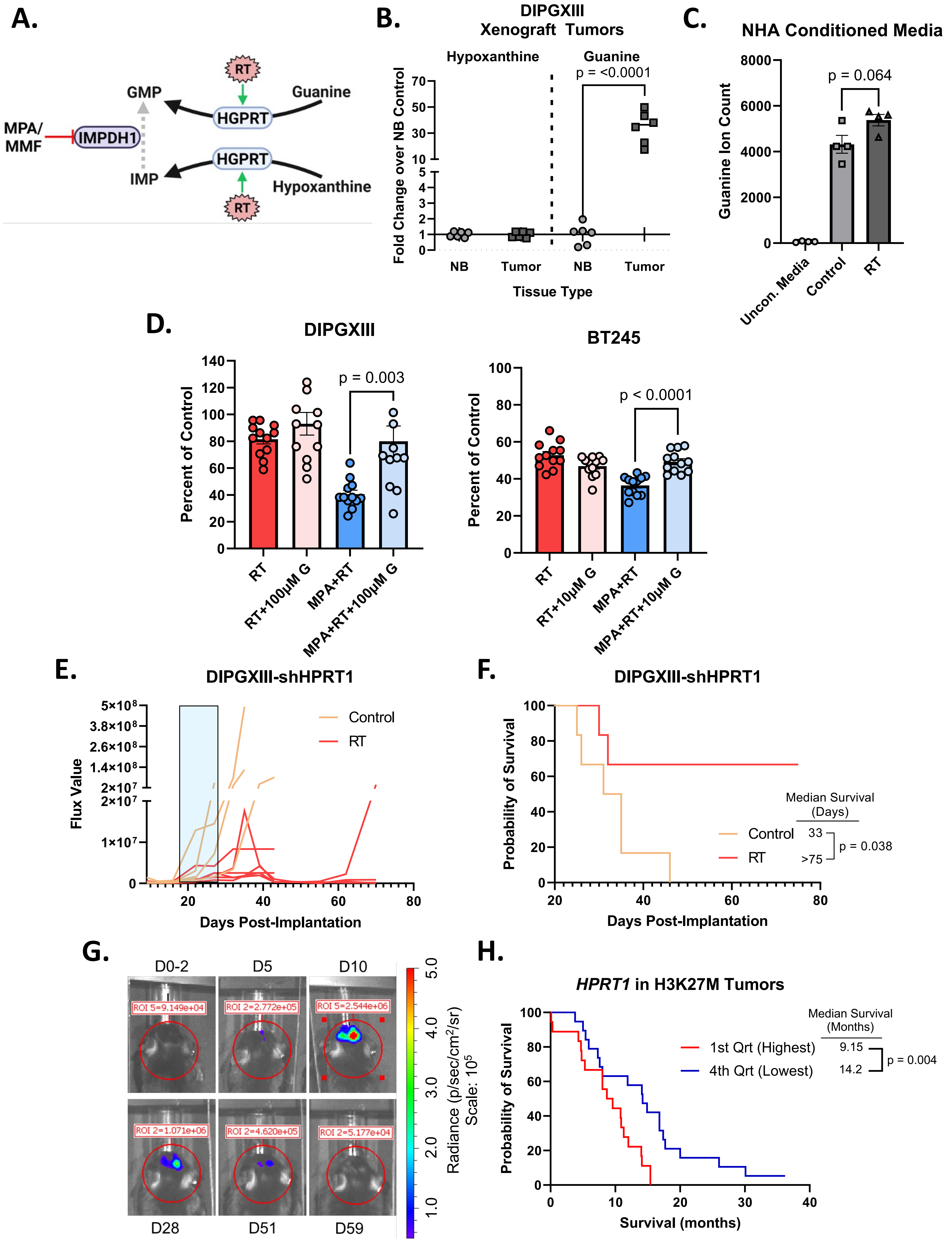
A.
RT
GMP
Guanine
HGPRT
MPA/
MMF
IMPDH1
HGPRT
IMP
Hypoxanthine
RT
B.
DIPGXIII
Xenograft Tumors
Hypoxanthine
Guanine
p = <0.0001
Fold Change over NB Control
NB
Tumor
NB
Tumor
Tissue Type
C.
NHA Conditioned Media
p = 0.064
Guanine Ion Count
Uncon. Media
Control
RT
D.
DIPGXIII
p = 0.003
Percent of Control
RT
RT+100μM G
MPA+RT
MPA+RT+100μM G
BT245
p < 0.0001
Percent of Control
RT
RT+10μM G
MPA+RT
MPA+RT+10μM G
E.
DIPGXIII-shHPRT1
Control
RT
Flux Value
Days Post-Implantation
F.
DIPGXIII-shHPRT1
Probability of Survival
Median Survival (Days)
Control 33
RT >75
p = 0.038
Days Post-Implantation
G.
D0-2
D5
D10
ROI 5=9.149e+04
ROI 2=2.772e+05
ROI 5=2.544e+06
ROI 2=1.071e+06
ROI 2=4.620e+05
ROI 2=5.177e+04
D28
D51
D59
Radiance (p/sec/cm²/sr)
Scale: 10⁵
H.
HPRT1 in H3K27M Tumors
Probability of Survival
Median Survival (Months)
1st Qrt (Highest) 9.15
4th Qrt (Lowest) 14.2
p = 0.004
Survival (months)

Supplemental Figures 1-14

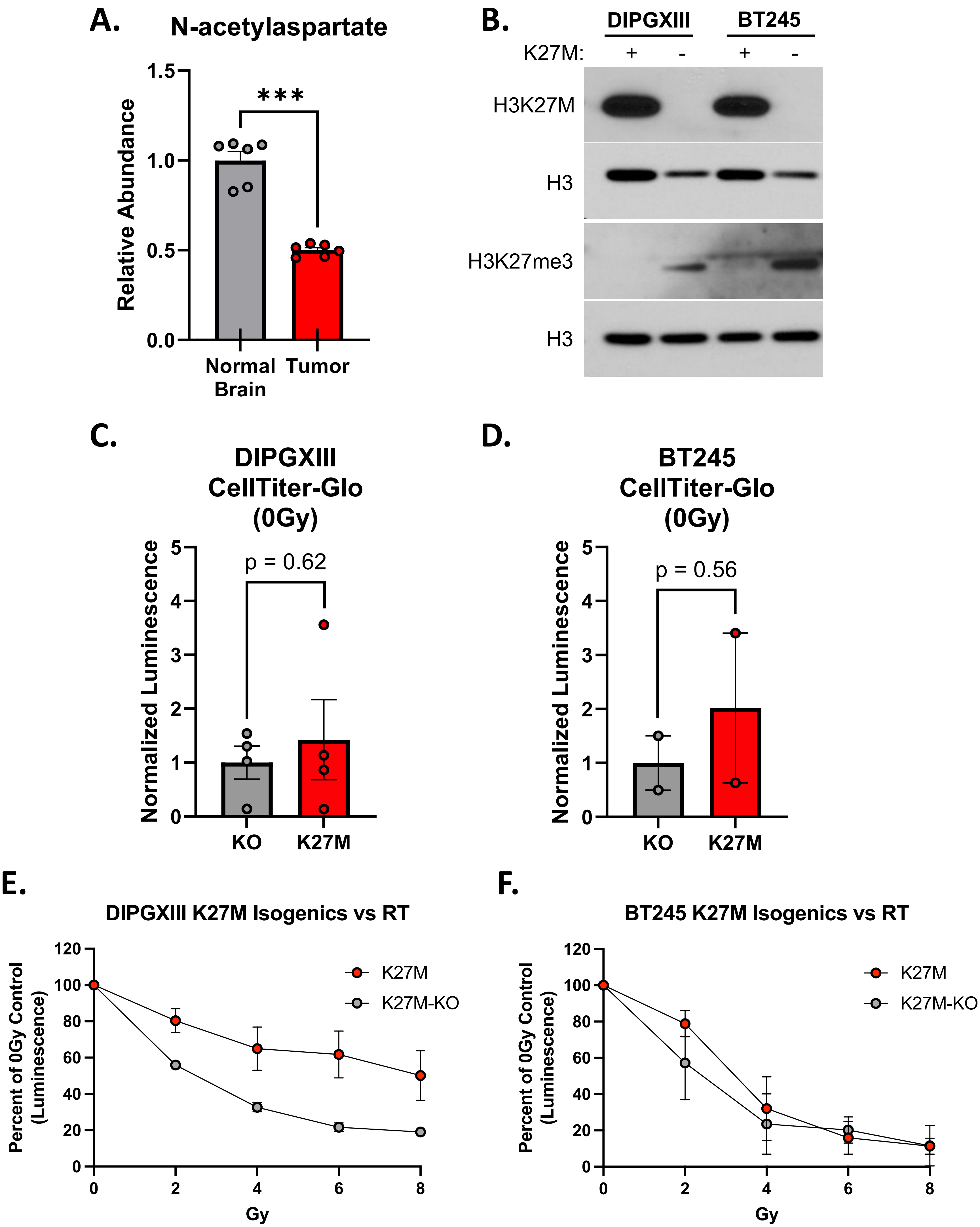

A.
N-acetylaspartate
***
Relative Abundance
1.5
1.0
0.5
0.0
Normal Brain
Tumor
B.
DIPGXIII
BT245
K27M:
+
-
+
-
H3K27M
H3
H3K27me3
H3
C.
DIPGXIII CellTiter-Glo (0Gy)
p = 0.62
Normalized Luminescence
KO
K27M
D.
BT245 CellTiter-Glo (0Gy)
p = 0.56
Normalized Luminescence
KO
K27M
E.
DIPGXIII K27M Isogenics vs RT
Percent of 0Gy Control (Luminescence)
K27M
K27M-KO
Gy
F.
BT245 K27M Isogenics vs RT
Percent of 0Gy Control (Luminescence)
K27M
K27M-KO
Gy

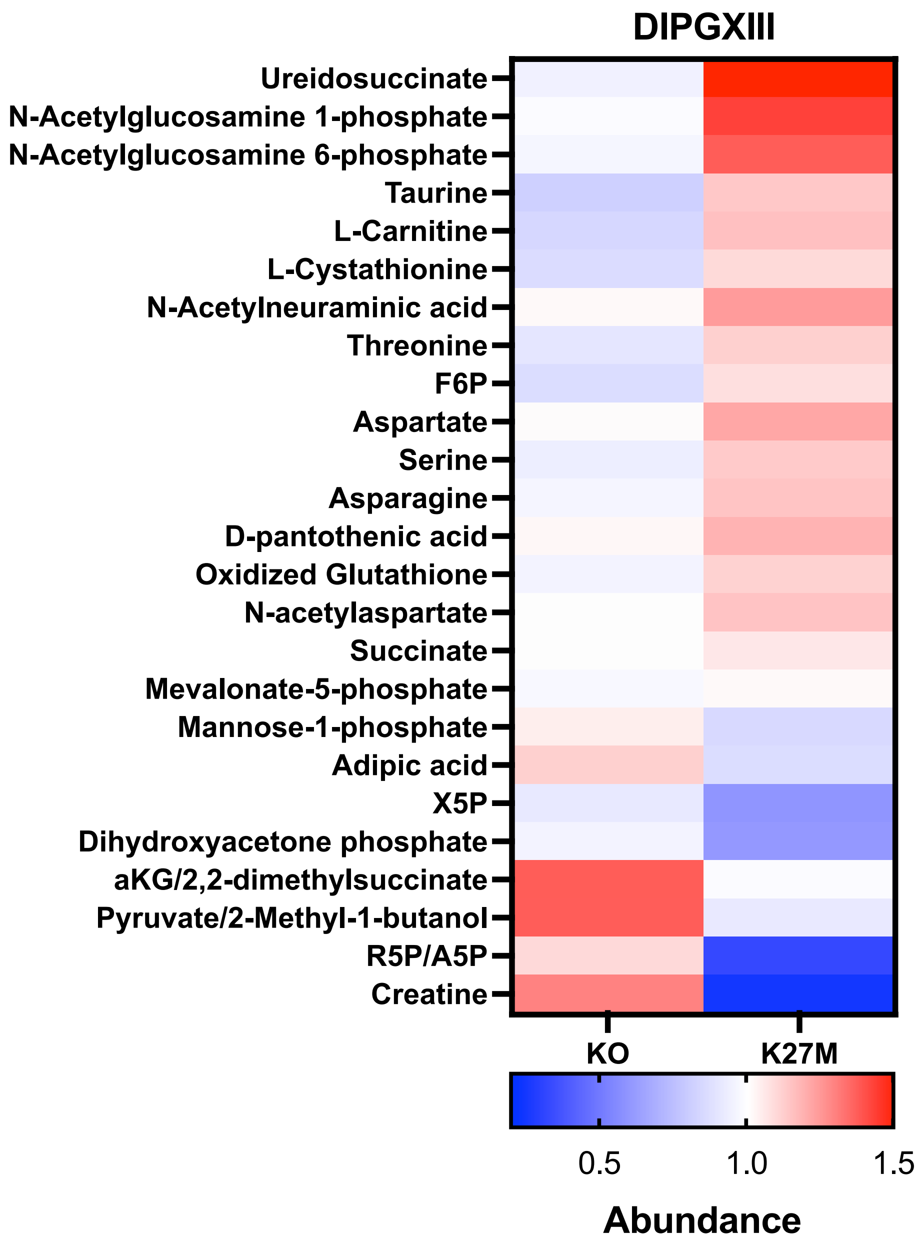
DIPGXIII
Ureidosuccinate
N-Acetylglucosamine 1-phosphate
N-Acetylglucosamine 6-phosphate
Taurine
L-Carnitine
L-Cystathionine
N-Acetylneuraminic acid
Threonine
F6P
Aspartate
Serine
Asparagine
D-pantothenic acid
Oxidized Glutathione
N-acetylaspartate
Succinate
Mevalonate-5-phosphate
Mannose-1-phosphate
Adipic acid
X5P
Dihydroxyacetone phosphate
aKG/2,2-dimethylsuccinate
Pyruvate/2-Methyl-1-butanol
R5P/A5P
Creatine
KO
K27M
0.5
1.0
1.5
Abundance

Supplemental Table 1: Baseline median-centered abundance fold changes for the top 25 metabolites between H3K27M-isogenic cells.

DIPGXIII Isogenics Baseline			
Metabolite	KO	K27M	Difference (K27M-KO)
Ureidosuccinate	0.943	1.533	0.590
N-Acetylglucosamine 1-phosphate	0.981	1.423	0.442
N-Acetylglucosamine 6-phosphate	0.962	1.368	0.406
Taurine	0.809	1.133	0.324
L-Carnitine	0.839	1.151	0.312
L-Cystathionine	0.857	1.092	0.235
N-Acetylneuraminic acid	1.016	1.238	0.222
Threonine	0.897	1.116	0.219
F6P	0.860	1.077	0.217
Aspartate	1.008	1.210	0.202
Serine	0.930	1.127	0.197
Asparagine	0.956	1.142	0.186
D-pantothenic acid	1.019	1.182	0.163
Oxidized Glutathione	0.952	1.110	0.157
N-acetylaspartate	1.001	1.144	0.143
Succinate	1.001	1.058	0.056
Mevalonate-5-phosphate	0.969	1.015	0.046
Mannose-1-phosphate	1.043	0.847	-0.196
Adipic acid	1.117	0.864	-0.252
X5P	0.908	0.594	-0.314
Dihydroxyacetone phosphate	0.953	0.604	-0.350
aKG/2,2-dimethylsuccinate	1.369	0.984	-0.385
Pyruvate/2-Methyl-1-butanol	1.366	0.915	-0.451
R5P/A5P	1.094	0.321	-0.773
Creatine	1.289	0.243	-1.046
BT245 Isogenics Baseline			
Metabolite	KO	K27M	Difference (K27M-KO)
Guanosine	0.512	2.364	1.851
Citramalic acid	0.418	1.034	0.616
Deoxycytidine	0.750	1.137	0.387
Histidine	0.996	1.300	0.304
Glutamine	0.909	1.133	0.224
4-Methyl-2-oxovaleric acid	0.860	1.039	0.179
Isopentyl acetate	0.892	1.044	0.152
Itaconic acid	0.927	1.068	0.141
Aspartate	1.008	0.883	-0.125
D-pantothenic acid	1.019	0.846	-0.173
Threonine	1.115	0.927	-0.188
Tyrosine	1.027	0.822	-0.205
Phenylalanine	1.009	0.787	-0.222
Methionine	1.007	0.765	-0.242
Reduced Glutathione	1.071	0.823	-0.248
Isoleucine	1.030	0.779	-0.251
N-Acetylglutamic acid	1.080	0.806	-0.274
N-Acetylglucosamine 6-phosphate	1.127	0.733	-0.394
Chorismic acid	1.045	0.645	-0.400
Adipic acid	1.152	0.698	-0.453
Creatine	1.363	0.850	-0.513
TDP	1.302	0.756	-0.545
Ureidosuccinate	1.381	0.810	-0.571
N-acetylaspartate	1.270	0.645	-0.625
N-acetylaspartylglutamate	1.325	0.626	-0.699

Supplemental Table 2: Post-RT abundance fold change for the top 25 metabolites between H3K27M-isogenic cell line pairs.

DIPGXIII Isogenics post-RT			
Metabolite	KO	K27M	Difference (K27M-KO)
Xanthine*	-0.664	0.367	1.031
Pyridoxine	-0.925	0.047	0.972
R5P/A5P*	0.856	1.736	0.879
X5P*	0.446	0.985	0.539
AMP*	-0.302	0.211	0.513
aKG/ 2,2-dimethylsuccinate	-0.441	0.006	0.448
UMP	-0.089	0.309	0.398
Hypoxanthine	0.378	0.768	0.390
G1P	0.109	0.402	0.293
Tyrosine	-0.525	-0.243	0.281
Allantoin	-0.165	0.109	0.275
Proline	-0.278	-0.007	0.271
dGDP/ADP*	-0.304	-0.067	0.237
β-NAD	-0.368	-0.199	0.169
Oxidized glutathione	0.015	-0.167	-0.182
Phosphoenolpyruvate	1.728	1.540	-0.188
Lactate	0.314	0.101	-0.213
Ureidosuccinate	-0.271	-0.558	-0.286
Pyruvate/2-Methyl-1-butanol	0.183	-0.133	-0.317
2-Phosphoglycerate	2.384	2.046	-0.338
Sedoheptulose-7-phosphate	0.150	-0.217	-0.366
Dihydroxyacetone phosphate	1.012	0.629	-0.383
G6P	0.212	-0.202	-0.414
Mannose-1-phosphate	0.722	0.269	-0.452
3-Hydroxyphenylacetic acid	2.895	2.379	-0.516
BT245 Isogenics post-RT			
Metabolite	KO	K27M	Difference (K27M-KO)
dGDP/ADP*	0.139	3.784	3.644
Guanine*	-0.762	0.040	0.802
Chorismic acid	0.137	0.784	0.647
R5P/A5P*	-0.866	-0.330	0.536
Creatine	-0.349	0.156	0.504
Hypoxanthine*	-0.180	0.186	0.366
Proline	-0.169	0.119	0.288
Lactate	0.148	-0.173	-0.321
F6P	0.266	-0.056	-0.322
Allantoin	0.055	-0.275	-0.330
Asparagine	-0.064	-0.408	-0.343
Oxidized Glutathione	0.280	-0.073	-0.353
Isopentyl acetate	0.216	-0.147	-0.364
Malonic acid	0.272	-0.094	-0.365
Histidine	-0.127	-0.535	-0.408
N-Acetylglucosamine 1-phosphate	0.248	-0.195	-0.443
Deoxycytidine	0.501	0.051	-0.450
Itaconic acid	0.186	-0.276	-0.462
4-Methyl-2-oxovaleric acid	0.269	-0.293	-0.562
Arginine	-0.026	-0.606	-0.581
L-Carnitine	0.005	-0.747	-0.752
L-Cystathionine	0.167	-0.906	-1.073
Guanosine*	0.112	-1.034	-1.147
NADH	-1.479	-2.783	-1.304
Citramalic acid	1.451	0.001	-1.450

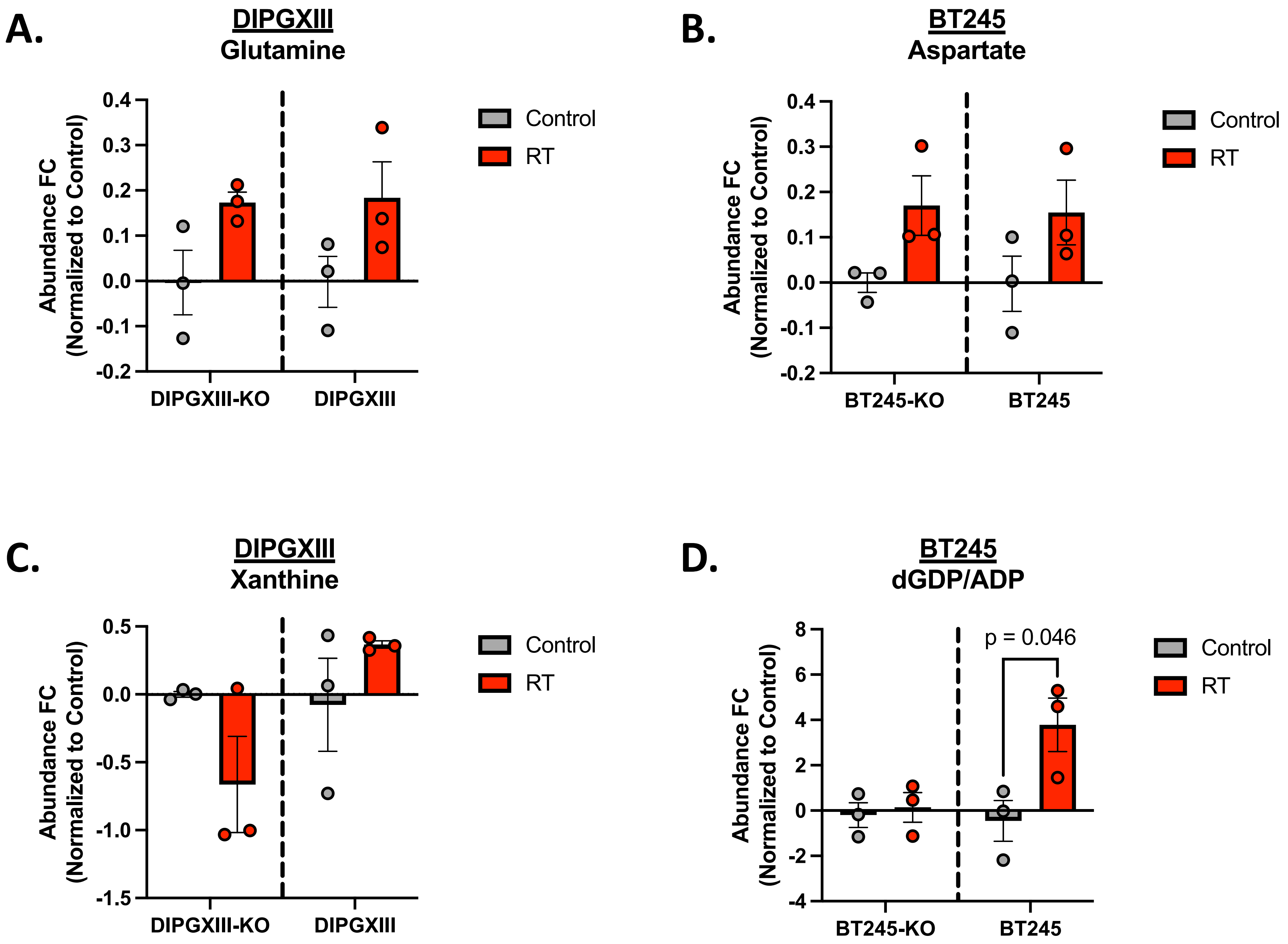
A.
DIPGXIII
Glutamine
Abundance FC
(Normalized to Control)
0.4
0.3
0.2
0.1
0.0
-0.1
-0.2
DIPGXIII-KO
DIPGXIII
Control
RT
B.
BT245
Aspartate
Abundance FC
(Normalized to Control)
0.4
0.3
0.2
0.1
0.0
-0.1
-0.2
BT245-KO
BT245
Control
RT
C.
DIPGXIII
Xanthine
Abundance FC
(Normalized to Control)
0.5
0.0
-0.5
-1.0
-1.5
DIPGXIII-KO
DIPGXIII
Control
RT
D.
BT245
dGDP/ADP
Abundance FC
(Normalized to Control)
8
6
4
2
0
-2
-4
p = 0.046
BT245-KO
BT245
Control
RT

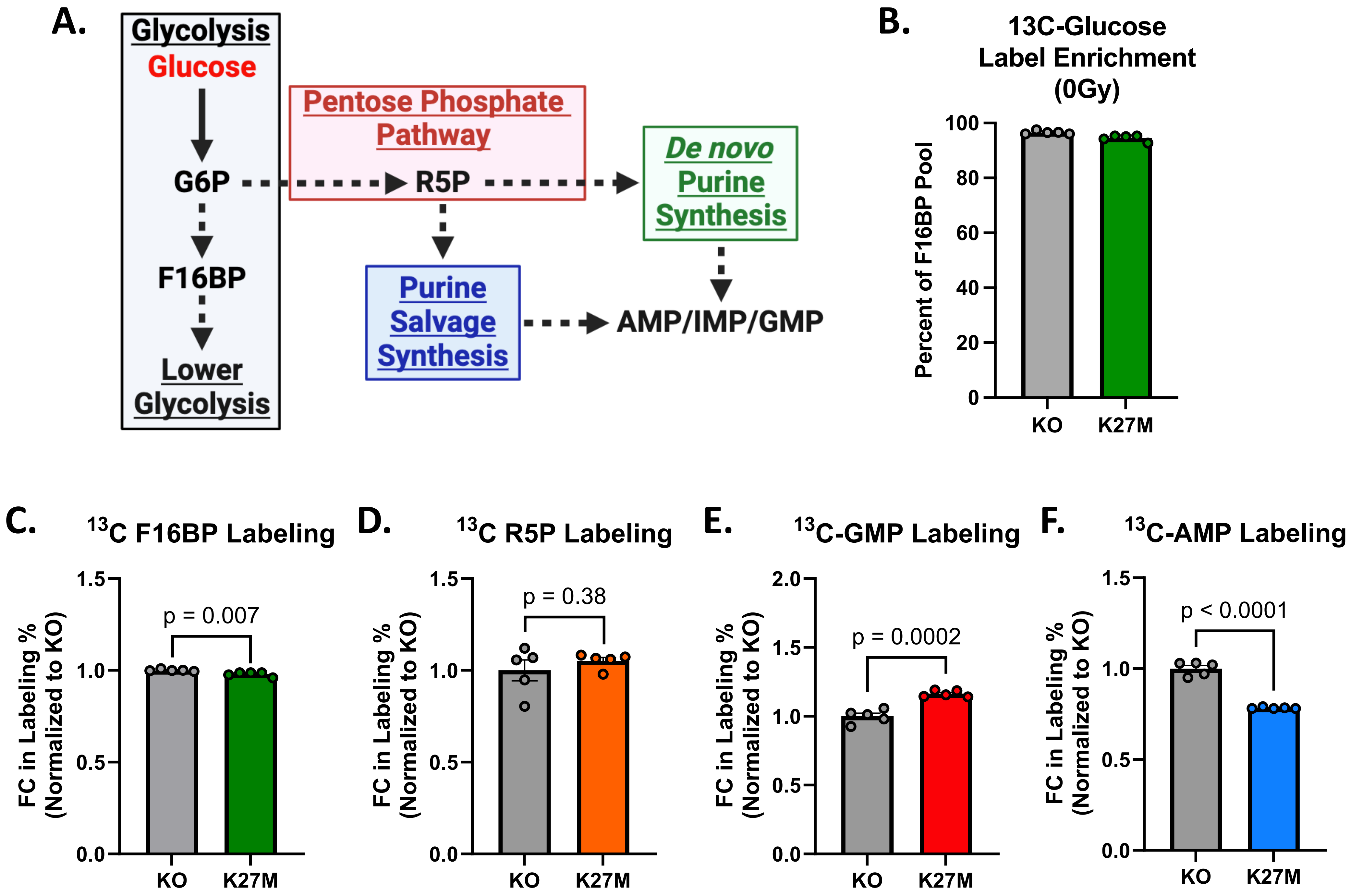
A.
Glycolysis
Glucose
G6P
F16BP
Lower Glycolysis
Pentose Phosphate Pathway
R5P
De novo Purine Synthesis
Purine Salvage Synthesis
AMP/IMP/GMP
B.
13C-Glucose Label Enrichment (0Gy)
Percent of F16BP Pool
100
80
60
40
20
0
KO
K27M
C.
13C F16BP Labeling
FC in Labeling % (Normalized to KO)
p = 0.007
D.
13C R5P Labeling
p = 0.38
E.
13C-GMP Labeling
p = 0.0002
F.
13C-AMP Labeling
p < 0.0001
KO
K27M

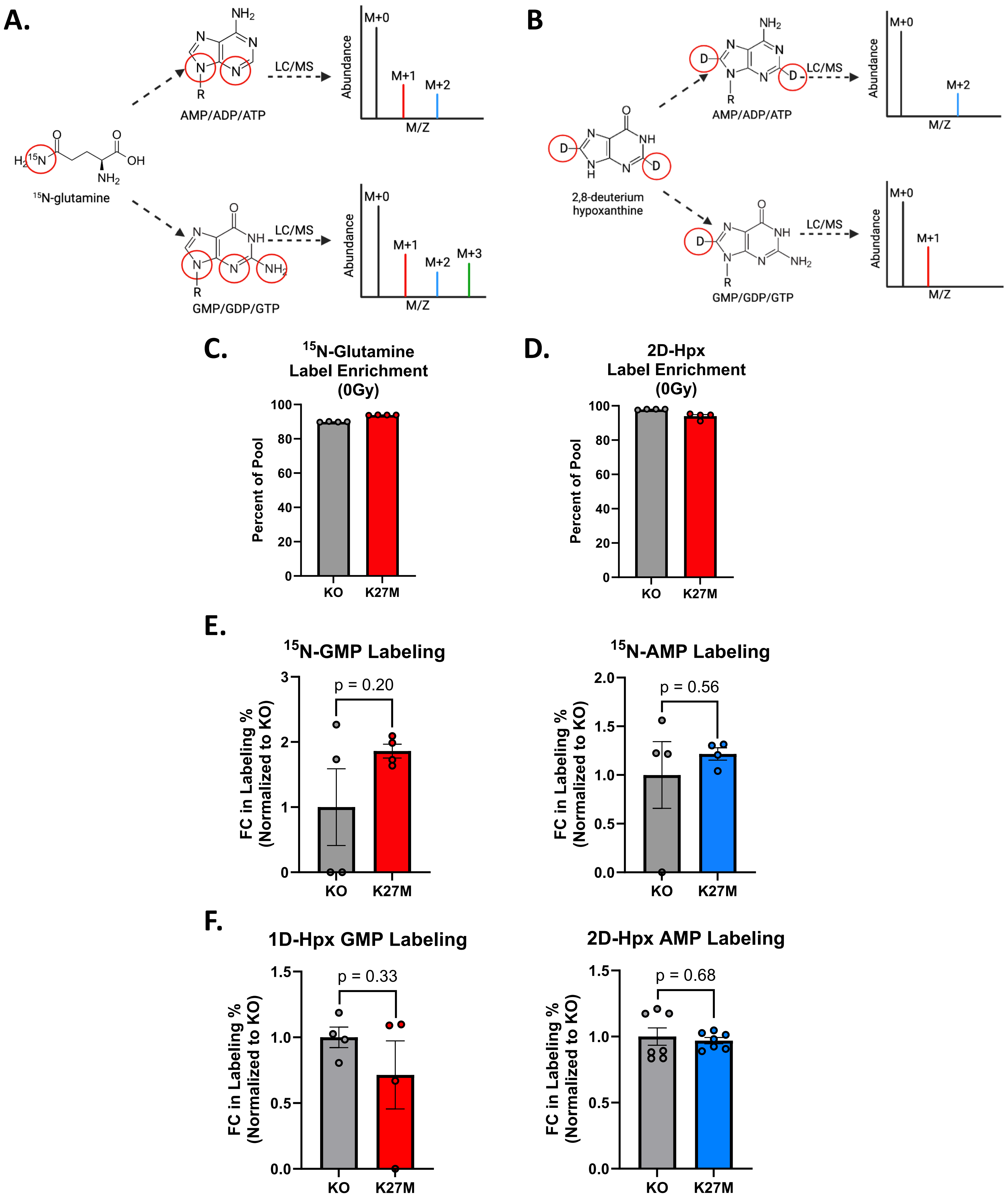
A.
NH2
N
N
N
N
R
AMP/ADP/ATP
LC/MS
M+0
M+1
M+2
Abundance
M/Z
H2 15N
O
O
OH
NH2
15N-glutamine
O
N
NH
N
N
NH2
R
GMP/GDP/GTP
LC/MS
M+0
M+1
M+2
M+3
Abundance
M/Z
B
NH2
N
N
D
N
N
D
R
AMP/ADP/ATP
LC/MS
M+0
M+2
Abundance
M/Z
O
D
N
NH
H
N
D
2,8-deuterium
hypoxanthine
O
N
NH
D
N
N
NH2
R
GMP/GDP/GTP
LC/MS
M+0
M+1
Abundance
M/Z
C.
15N-Glutamine
Label Enrichment
(0Gy)
Percent of Pool
100
80
60
40
20
0
KO
K27M
D.
2D-Hpx
Label Enrichment
(0Gy)
Percent of Pool
100
80
60
40
20
0
KO
K27M
E.
15N-GMP Labeling
p = 0.20
FC in Labeling %
(Normalized to KO)
3
2
1
0
KO
K27M
15N-AMP Labeling
p = 0.56
FC in Labeling %
(Normalized to KO)
2.0
1.5
1.0
0.5
0.0
KO
K27M
F.
1D-Hpx GMP Labeling
p = 0.33
FC in Labeling %
(Normalized to KO)
1.5
1.0
0.5
0.0
KO
K27M
2D-Hpx AMP Labeling
p = 0.68
FC in Labeling %
(Normalized to KO)
1.5
1.0
0.5
0.0
KO
K27M

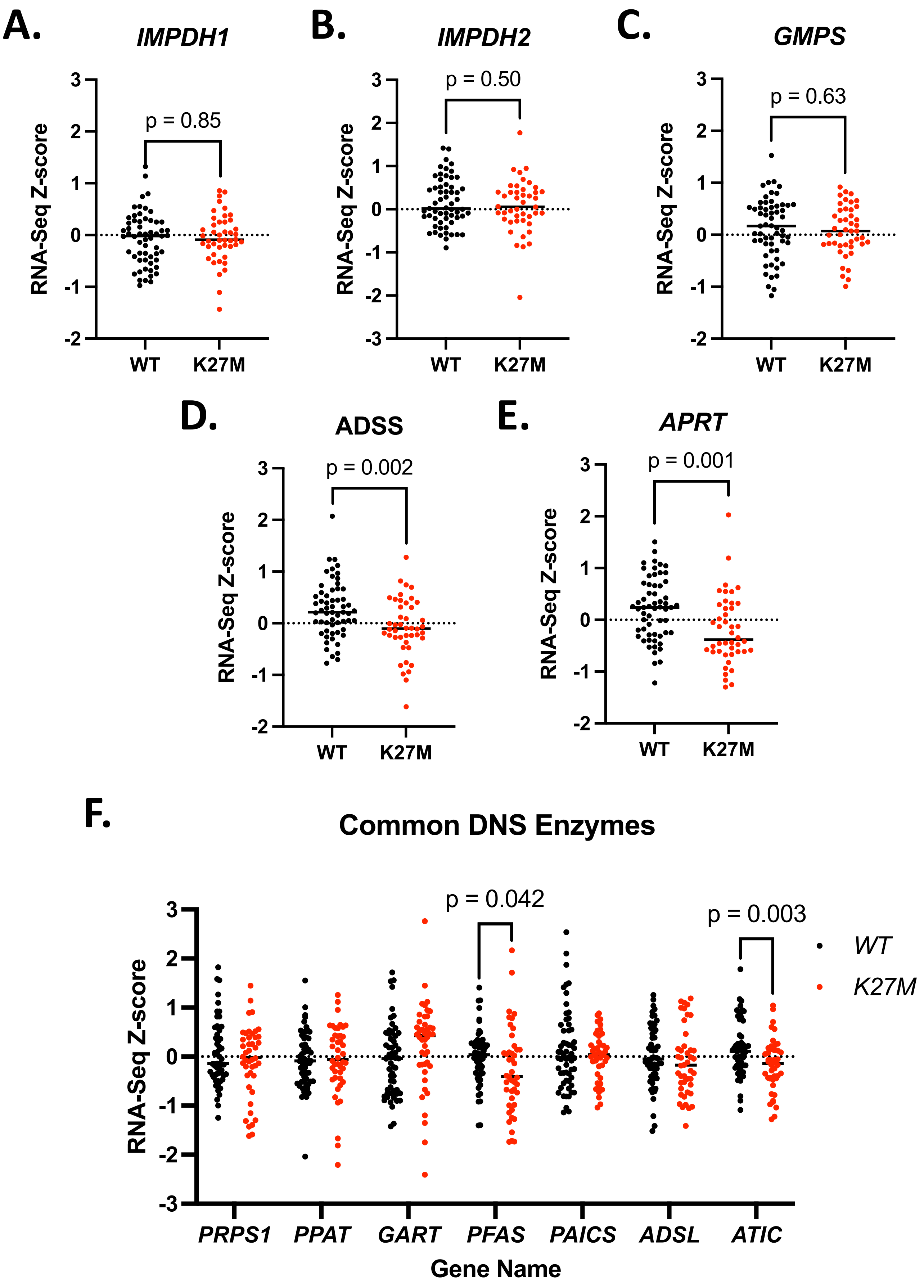
A.
IMPDH1
p = 0.85
RNA-Seq Z-score
WT
K27M
B.
IMPDH2
p = 0.50
RNA-Seq Z-score
WT
K27M
C.
GMPS
p = 0.63
RNA-Seq Z-score
WT
K27M
D.
ADSS
p = 0.002
RNA-Seq Z-score
WT
K27M
E.
APRT
p = 0.001
RNA-Seq Z-score
WT
K27M
F.
Common DNS Enzymes
p = 0.042
p = 0.003
WT
K27M
RNA-Seq Z-score
PRPS1
PPAT
GART
PFAS
PAICS
ADSL
ATIC
Gene Name

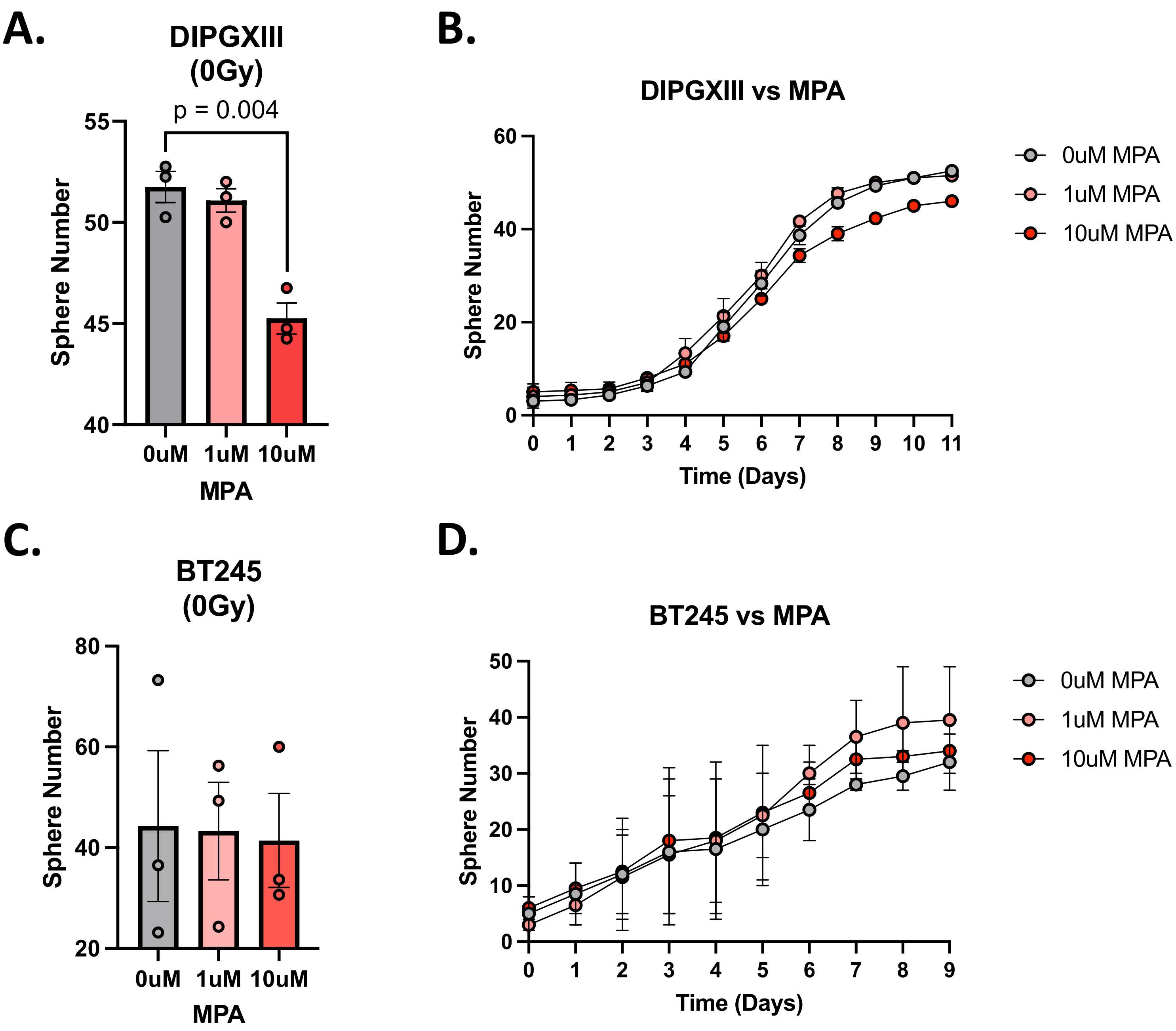
A.
DIPGXIII
(0Gy)
p = 0.004
Sphere Number
55
50
45
40
0uM
1uM
10uM
MPA
B.
DIPGXIII vs MPA
Sphere Number
60
40
20
0
0
1
2
3
4
5
6
7
8
9
10
11
Time (Days)
0uM MPA
1uM MPA
10uM MPA
C.
BT245
(0Gy)
Sphere Number
80
60
40
20
0uM
1uM
10uM
MPA
D.
BT245 vs MPA
Sphere Number
50
40
30
20
10
0
0
1
2
3
4
5
6
7
8
9
Time (Days)
0uM MPA
1uM MPA
10uM MPA

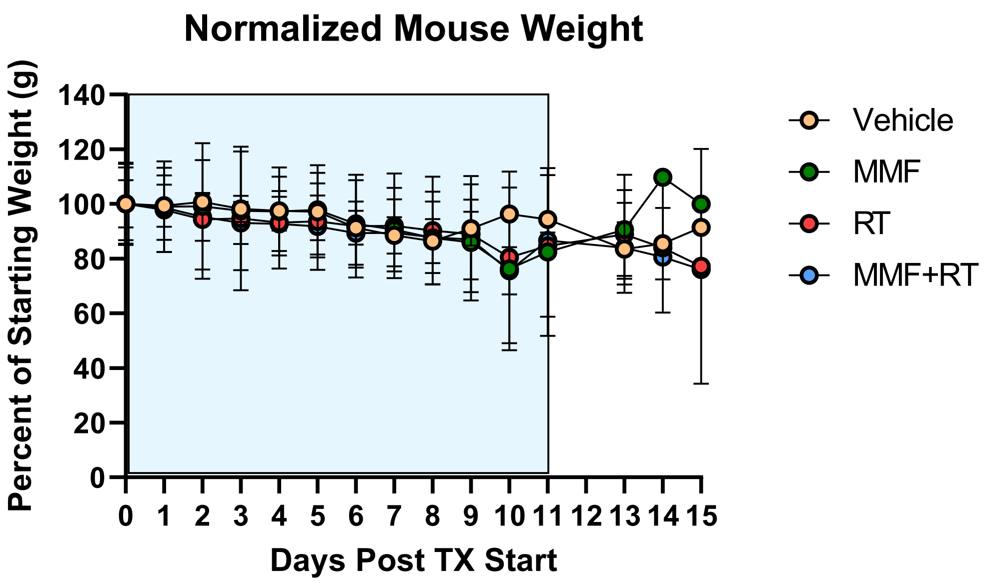

Normalized Mouse Weight
Percent of Starting Weight (g)
140
120
100
80
60
40
20
0
0 1 2 3 4 5 6 7 8 9 10 11 12 13 14 15
Days Post TX Start
Vehicle
MMF
RT
MMF+RT

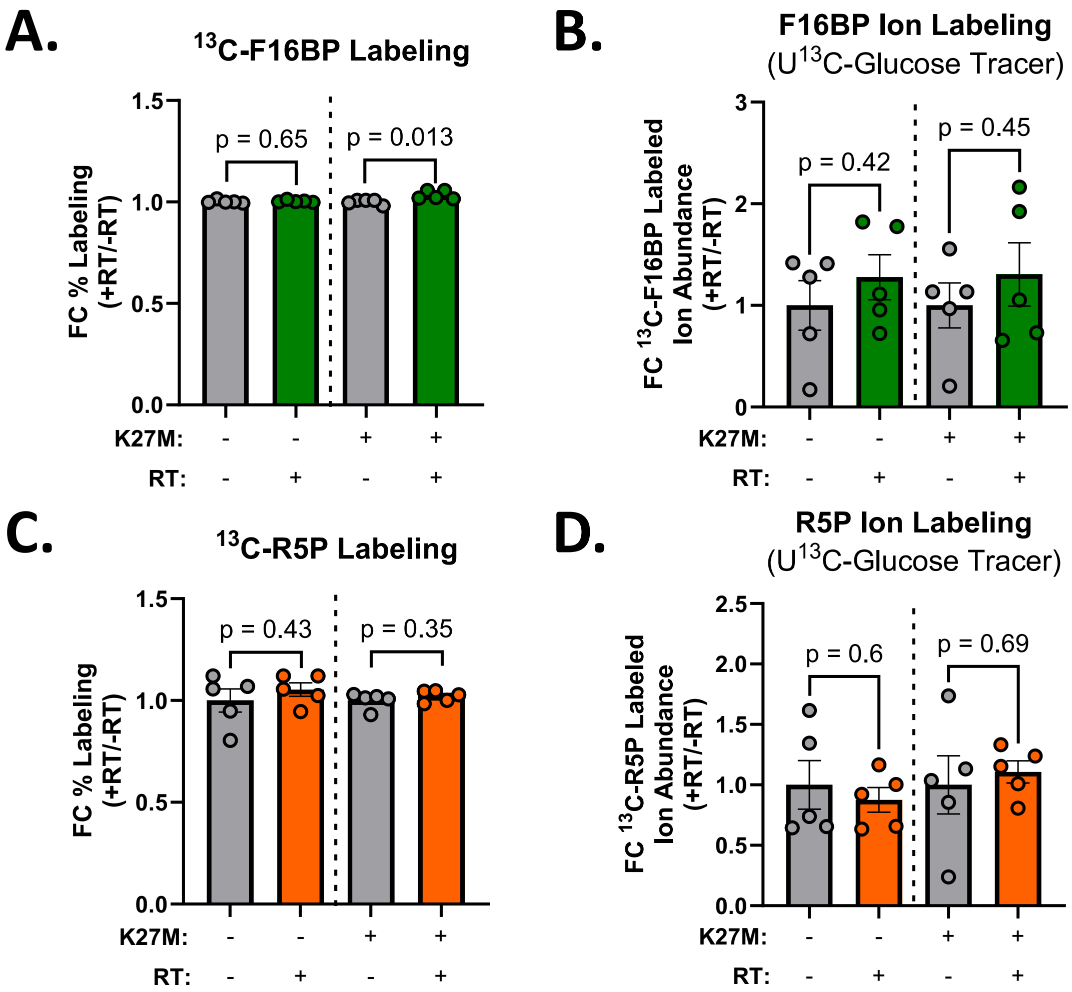
A.
13C-F16BP Labeling
FC % Labeling (+RT/-RT)
p = 0.65
p = 0.013
B.
F16BP Ion Labeling
(U13C-Glucose Tracer)
FC 13C-F16BP Labeled Ion Abundance (+RT/-RT)
p = 0.42
p = 0.45
C.
13C-R5P Labeling
FC % Labeling (+RT/-RT)
p = 0.43
p = 0.35
D.
R5P Ion Labeling
(U13C-Glucose Tracer)
FC 13C-R5P Labeled Ion Abundance (+RT/-RT)
p = 0.6
p = 0.69
K27M:
RT:

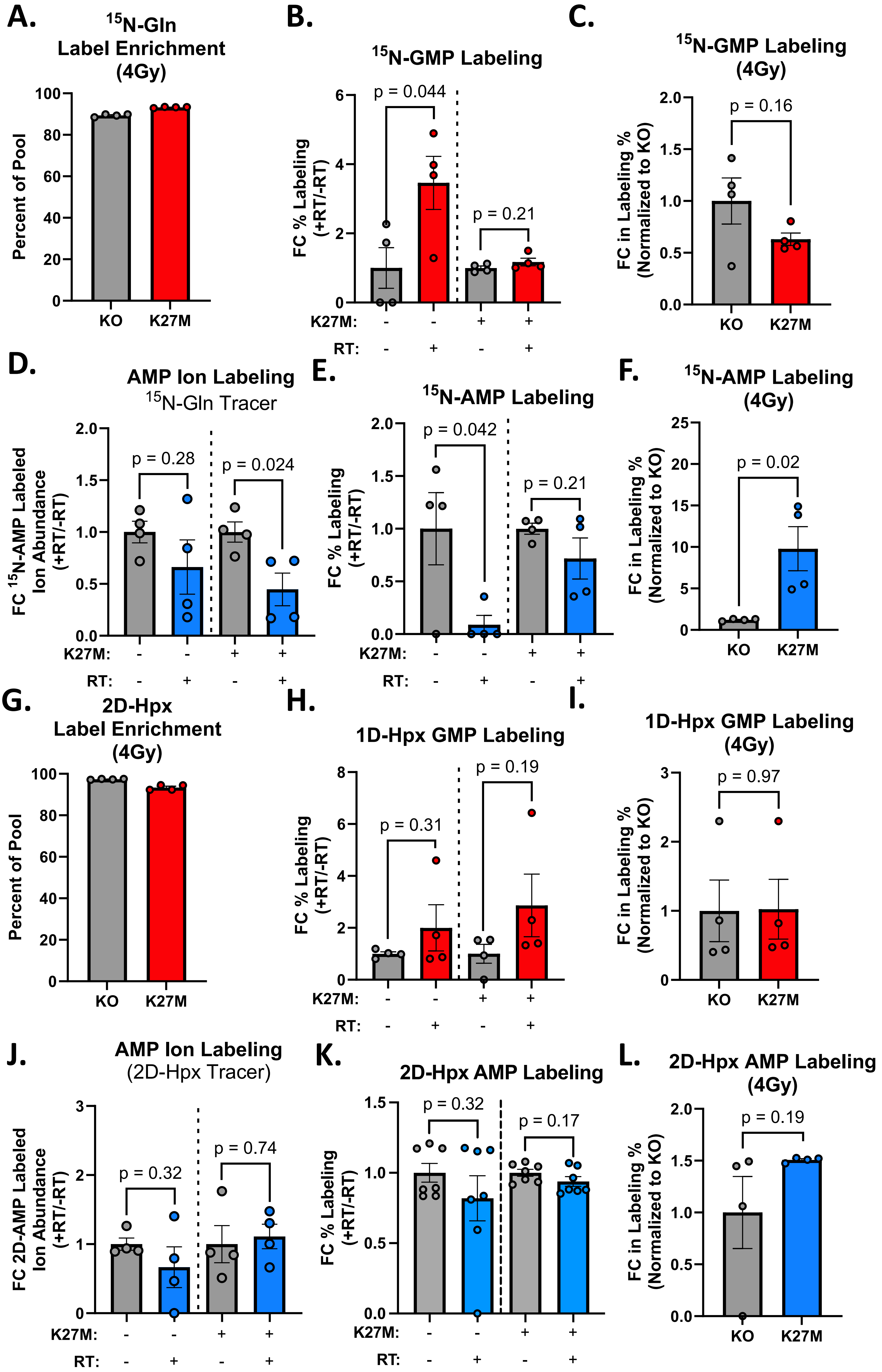
A.
^{15}N-Gln Label Enrichment (4Gy)
Percent of Pool
KO
K27M
B.
^{15}N-GMP Labeling
FC % Labeling (+RT/-RT)
p = 0.044
p = 0.21
K27M:
RT:
C.
^{15}N-GMP Labeling (4Gy)
FC in Labeling % (Normalized to KO)
p = 0.16
KO
K27M
D.
AMP Ion Labeling
^{15}N-Gln Tracer
FC ^{15}N-AMP Labeled Ion Abundance (+RT/-RT)
p = 0.28
p = 0.024
K27M:
RT:
E.
^{15}N-AMP Labeling
FC % Labeling (+RT/-RT)
p = 0.042
p = 0.21
K27M:
RT:
F.
^{15}N-AMP Labeling (4Gy)
FC in Labeling % (Normalized to KO)
p = 0.02
KO
K27M
G.
2D-Hpx Label Enrichment (4Gy)
Percent of Pool
KO
K27M
H.
1D-Hpx GMP Labeling
FC % Labeling (+RT/-RT)
p = 0.31
p = 0.19
K27M:
RT:
I.
1D-Hpx GMP Labeling (4Gy)
FC in Labeling % (Normalized to KO)
p = 0.97
KO
K27M
J.
AMP Ion Labeling
(2D-Hpx Tracer)
FC 2D-AMP Labeled Ion Abundance (+RT/-RT)
p = 0.32
p = 0.74
K27M:
RT:
K.
2D-Hpx AMP Labeling
FC % Labeling (+RT/-RT)
p = 0.32
p = 0.17
K27M:
RT:
L.
2D-Hpx AMP Labeling (4Gy)
FC in Labeling % (Normalized to KO)
p = 0.19
KO
K27M

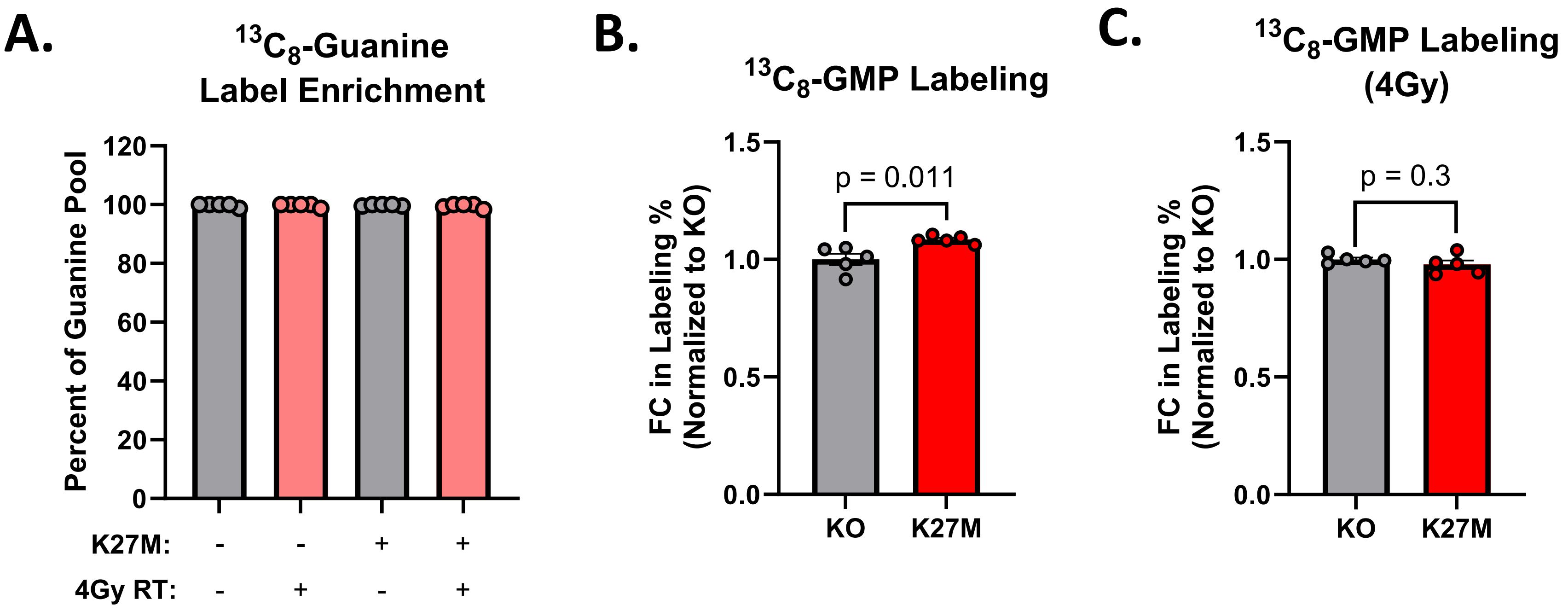
A.
13C8-Guanine
Label Enrichment
Percent of Guanine Pool
0
20
40
60
80
100
120
K27M: - - + +
4Gy RT: - + - +
B.
13C8-GMP Labeling
FC in Labeling %
(Normalized to KO)
0.0
0.5
1.0
1.5
p = 0.011
KO
K27M
C.
13C8-GMP Labeling
(4Gy)
FC in Labeling %
(Normalized to KO)
0.0
0.5
1.0
1.5
p = 0.3
KO
K27M

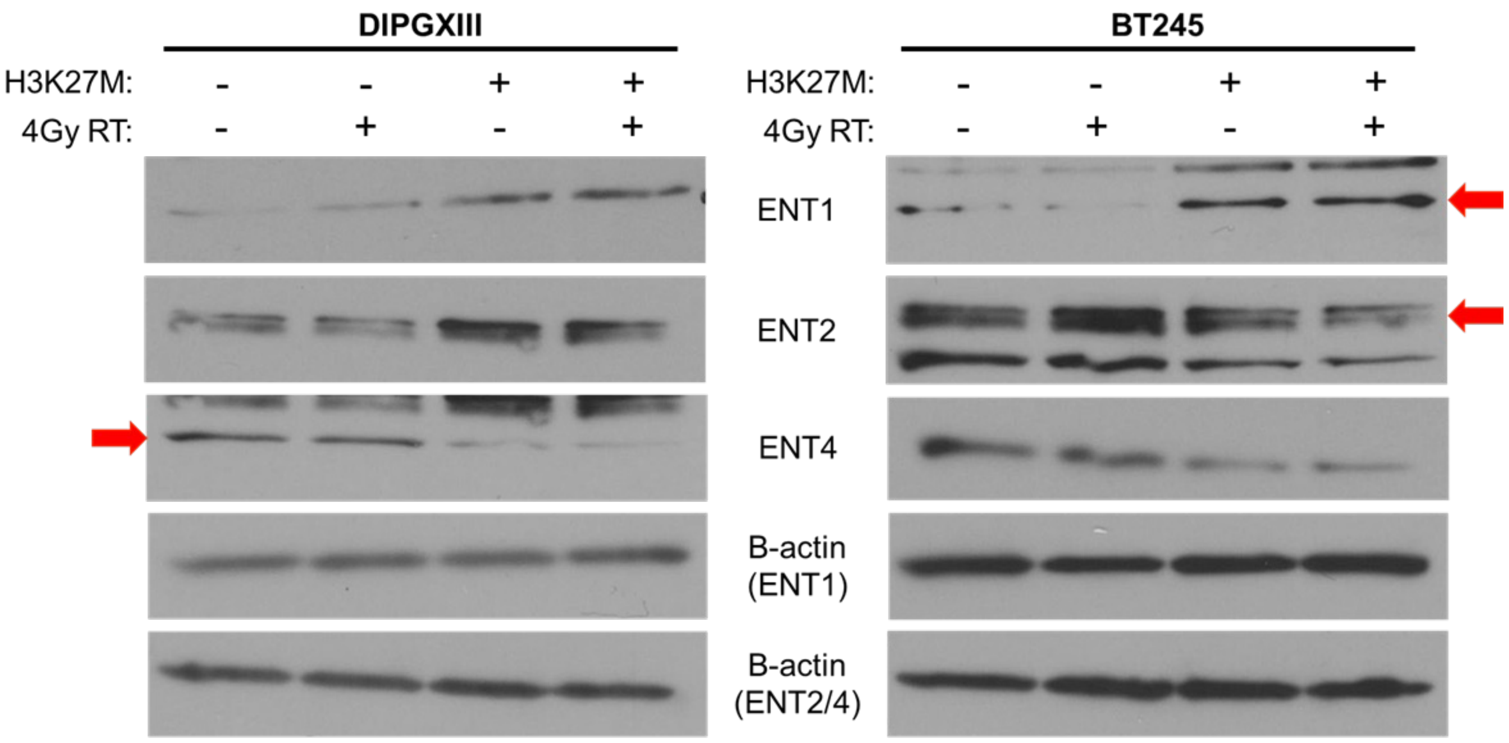
DIPGXIII
BT245
H3K27M:
4Gy RT:
ENT1
ENT2
ENT4
B-actin (ENT1)
B-actin (ENT2/4)

A.

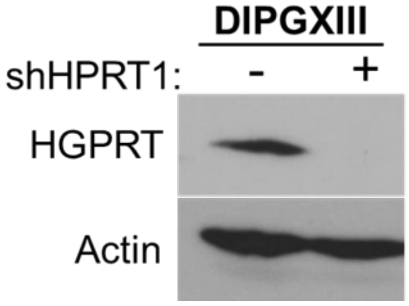

DIPGXIII
shHPRT1:
-
+
HGPRT
Actin

B.

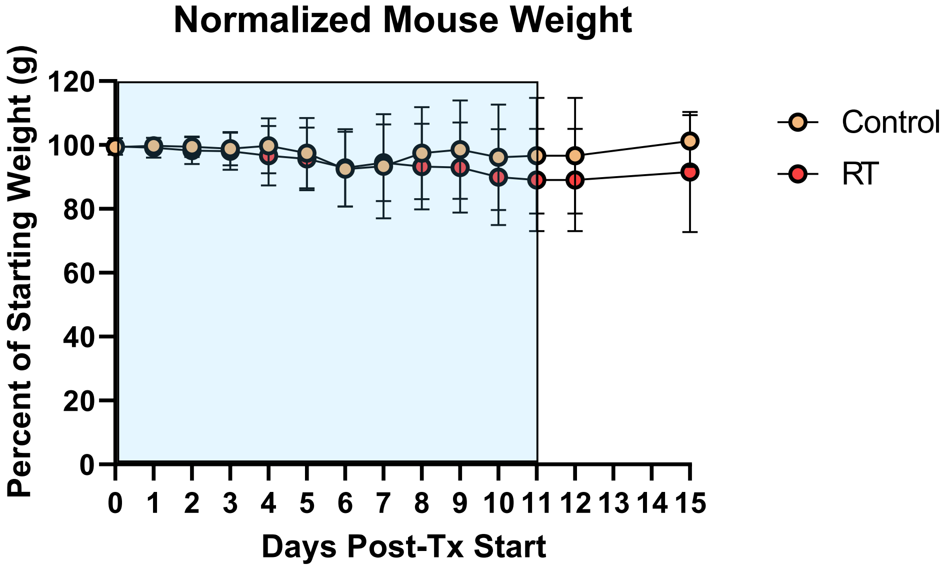

Normalized Mouse Weight
Percent of Starting Weight (g)
120
100
80
60
40
20
0
0 1 2 3 4 5 6 7 8 9 10 11 12 13 14 15
Days Post-Tx Start
Control
RT

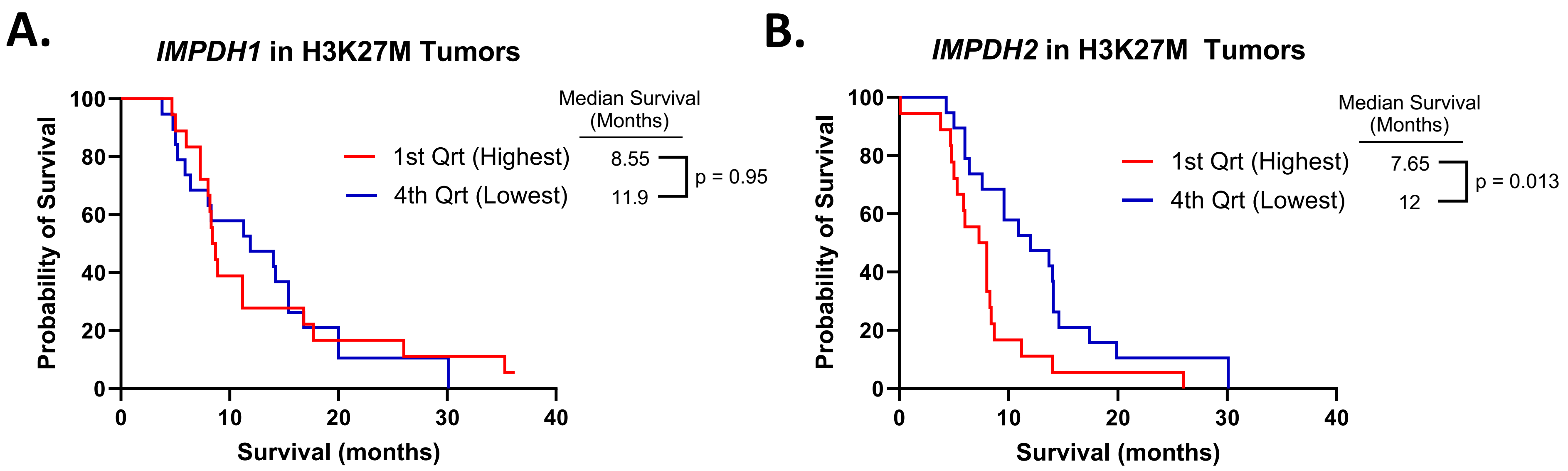
A.
IMPDH1 in H3K27M Tumors
Median Survival (Months)
1st Qrt (Highest) 8.55
4th Qrt (Lowest) 11.9
p = 0.95
Probability of Survival
Survival (months)
B.
IMPDH2 in H3K27M Tumors
Median Survival (Months)
1st Qrt (Highest) 7.65
4th Qrt (Lowest) 12
p = 0.013
Probability of Survival
Survival (months)
